# App-Based Smoking Urge Reduction Intervention for Young Adults: Protocol Combining a Microrandomized Trial and Conventional Between-Subject Randomized Trial

**DOI:** 10.2196/74388

**Published:** 2025-09-23

**Authors:** Johannes Thrul, Janardan Devkota, Joseph J C Waring, Michael R Desjardins, Josef Hamoud, Jasmin Han, Felix Naughton, Vadim Zipunnikov, Tamar Mendelson, Carl Latkin, David Epstein, Meghan Moran

**Affiliations:** 1 Department of Mental Health Bloomberg School of Public Health Johns Hopkins University Baltimore, MD United States; 2 Sidney Kimmel Comprehensive Cancer Center Johns Hopkins University Baltimore, MD United States; 3 Centre for Alcohol Policy Research La Trobe University Melbourne Australia; 4 Department of Epidemiology Bloomberg School of Public Health Johns Hopkins University Baltimore, MD United States; 5 Spatial Science for Public Health Center Bloomberg School of Public Health Johns Hopkins University Baltimore, MD United States; 6 Department of Medical Statistics Faculty of Medicine University of Göttingen Göttingen Germany; 7 Addiction Research Group University of East Anglia Norwich United Kingdom; 8 Department of Biostatistics Bloomberg School of Public Health Johns Hopkins University Baltimore, MD United States; 9 Department of Health, Behavior and Society Bloomberg School of Public Health Johns Hopkins University Baltimore, MD United States; 10 Real-World Assessment, Prediction, and Treatment Unit Intramural Research Program National Institute on Drug Abuse Baltimore, MD United States

**Keywords:** cigarette smoking, young adults, microrandomized trial, ecological momentary assessment, smartphone

## Abstract

**Background:**

Tobacco smoking is the leading preventable cause of morbidity and mortality in the United States, and young adults have high smoking rates. Although most young adult smokers are interested in quitting, they underutilize professional cessation support. Smartphones have wide reach and integration into young adults’ lives, and these devices offer great opportunities to deliver cessation interventions by delivering messages suggesting coping strategies “in the moment” when smokers need cessation support.

**Objective:**

The overall goal of this trial is to evaluate the efficacy of cognitive behavioral therapy (CBT) and mindfulness or acceptance and commitment therapy (ACT) messages for young adults targeted at specific high-risk situations for smoking.

**Methods:**

We will conduct a microrandomized trial (MRT; within-subject randomization) to test the efficacy of CBT and mindfulness or ACT compared with control messages for reducing smoking urge up to 15 minutes after message delivery, nested in a conventional between-subject randomized controlled trial (RCT). A conventional between-subject control group of participants who will complete ecological momentary assessment (EMA) only without intervention messages will allow us to test if messages reduce cigarettes per day at the end of treatment, 3-month follow-up, and 6-month follow-up. Among MRT intervention group participants, we will explore how message efficacy may be moderated by substance co-use (cannabis, alcohol, other drugs) and exposure to specific settings (home, work, bars).

**Results:**

As of June 2025, we had enrolled 58 participants of the target sample of 160, with 52% (30/58) assigned to the MRT group and 48% (28/58) assigned to the EMA-only control.

**Conclusions:**

Smoking onset is now more common among young adults than adolescents, and early cessation substantially reduces morbidity and mortality from smoking, making age-appropriate, tailored, and scalable interventions for this high-priority population even more important. Results of this trial will provide evidence on the efficacy of tailored intervention messages to help young adult smokers cope with smoking urges as an integral part of smartphone interventions. Findings will inform the field about key principles, strategies, and efficacy of situational tailoring of app-based tobacco use urge reduction messages.

**Trial Registration:**

ClinicalTrials.gov NCT05836103; https://clinicaltrials.gov/study/NCT05836103

**International Registered Report Identifier (IRRID):**

DERR1-10.2196/74388

## Introduction

### Background

Tobacco smoking is the leading preventable cause of morbidity and mortality in the United States, and young adults have high smoking rates [1]. Smoking onset is now more common among young adults than adolescents [2], and early cessation substantially reduces morbidity and mortality from smoking [3]. Although most young adult smokers are interested in quitting [4], they underutilize professional cessation support [5,6]. We need novel approaches to deliver evidence-based smoking cessation interventions to young adults [7,8]. Smartphones have wide reach and integration into young adults’ lives (98% own a smartphone) [9]. These devices offer great opportunities to deliver cessation interventions by delivering messages suggesting coping strategies “in the moment” when smokers need cessation support. However, few cessation apps deliver evidence-based intervention content [10,11] and content tailored to individual needs [12-14]. Moreover, mobile smoking cessation interventions have yet to account for the impact of substance co-use (eg, alcohol, cannabis), which is frequent among young adults [15,16], on intervention effects.

One especially promising strategy for smartphone interventions is to target situations that elicit smoking urges. In our [17,18] and others’ [19-22] prior work, these urges emerged as the most important triggers of smoking. The probability of smoking greatly increases as urge levels rise [23], especially among light smokers [24], who are common among young adults [25]. It is thus paramount for smartphone interventions to help young adults cope with these smoking urges. Clinical practice guidelines for smoking cessation [26] emphasize cognitive behavioral therapy (CBT) to help patients develop coping strategies for urges [27]. Mindfulness or acceptance and commitment therapy (ACT) offers a different approach, which teaches smokers psychological flexibility through accepting negative experiences [28]. Although there is evidence for the efficacy of both CBT and mindfulness or ACT smoking cessation interventions [29], it is unclear if these approaches are efficacious when implemented in real time and with young adults. The overall goal of this study is to evaluate the efficacy of CBT and mindfulness or ACT messages for young adults targeted at specific high-risk situations for smoking.

To evaluate the real-time impact of smartphone-based intervention messages on smoking urges, an alternative study design to traditional between-subject randomized controlled trials (RCTs) is needed. Microrandomized trials (MRTs) [30,31] offer an innovative factorial design appropriate for testing dynamic, context-sensitive interventions, including smartphone-based smoking cessation. Unlike static RCTs, MRTs use repeated within-subject randomizations, allowing intervention effects to be assessed across real-world situations experienced by participants as they go about their daily lives. By integrating geofence-triggered message delivery [32,33] (using GPS-defined virtual boundaries that activate messages when participants enter specific areas) and real-time data collection via ecological momentary assessment (EMA) [17,18,34,35], MRTs generate rich data on intervention message impact in near real time. This design supports causal inference and can help to identify the most effective strategies for tailoring messages to improve smoking cessation outcomes among young adults.

This study leverages a Health Insurance Portability and Accountability Act (HIPAA)–compliant mobile research platform designed for EMA studies, which enables real-time data collection, GPS tracking, and implementation of MRTs. This platform allows us to identify high-risk smoking situations using EMA, as well as time and location data; define geofences around these locations; and deliver CBT- and ACT-based intervention messages. By combining smartphone sensor data with within-subject randomization of message delivery, the platform enables us to test the causal impact of intervention messages on real-time smoking urges among young adults and addresses a critical gap in existing mobile health research.

Our team recently demonstrated the feasibility of determining high-risk situations for smoking and delivering tailored messages based on geofence triggers (virtual boundaries by GPS that trigger a message when a mobile device enters the area) [32]. Moreover, we conducted a recent successful pilot study that showed the feasibility of an app-based MRT using intervention messages triggered by geofence locations using GPS [36]. These feasibility studies inform this fully powered MRT to investigate message efficacy to reduce smoking urges and cigarette smoking behavior in young adults. The trial will address the specific aims described in the following sections.

### Aims and Hypotheses

#### Aim 1: To Test CBT and Mindfulness or ACT Intervention Message Efficacy for Reducing Momentary Smoking Urges (n=80)

To inform just-in-time interventions, it is crucial to test if CBT- and mindfulness or ACT-based messages can reduce momentary smoking urges. We will conduct an MRT (repeated within-subject randomizations of messages) to accomplish this [30,37]. In line with our existing protocol, participants first collect EMA data for 14 days, allowing us to determine high-risk situations for smoking. In the following intervention phase, participants receive tailored messages triggered by geofencing of participants’ high-risk locations for a total of 30 days. Tailoring is based on established predictors of smoking relapse (stress and presence of other smokers) [38-40]. The MRT tests the efficacy of CBT versus mindfulness or ACT versus control messages for reducing smoking urge up to 15 minutes after message delivery. Secondary outcomes include smoking or other tobacco use (including e-cigarettes), affect, and stress.

#### Aim 2: To Test if Exposure to Urge Reduction Messages Results in Changes in Smoking Behavior Over Time Compared With an EMA-Only Control Group (n=80)

It is important to investigate if repeated messages in the MRT impact smoking behavior over time, in contrast to repeated assessment without messages. Thus, this study includes a conventional RCT component. Parallel to the MRT group, a control group completes EMA surveys only without intervention messages. This allows us to test if messages reduce smoking behavior. The primary outcome is number of cigarettes per day at the end of treatment, a 3-month follow-up, and a 6-month follow-up. Secondary analyses explore biochemically verified 7-day point prevalence abstinence, switching to e-cigarettes, and other tobacco outcomes. Post hoc dose-response analyses investigate the long-term efficacy of CBT and mindfulness or ACT messages on smoking behavior.

#### Aim 3: Explore Moderation Effects of Substance Co-Use (Cannabis, Alcohol, Other Drugs) and Exposure to Specific Location (Home, Work, Bars) on Urge Reduction Message Efficacy

A crucial research question to inform future mobile interventions is how well intervention messages work in different situational contexts and when people are co-using other substances. Among MRT intervention group participants, we will explore how urge reduction message efficacy may be moderated by substance co-use and exposure to specific settings.

## Methods

### Overview of Design

**Figure 1:**
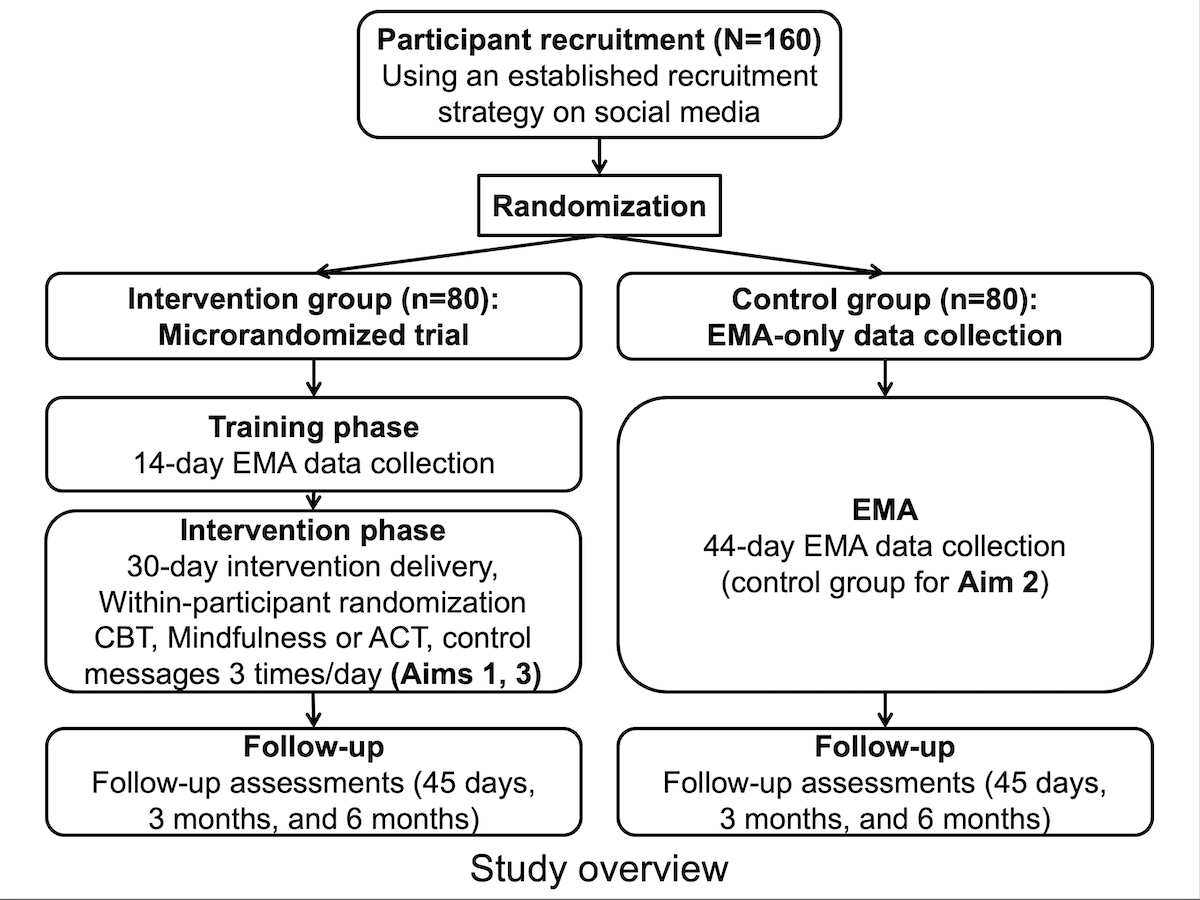
Study overview. ACT: acceptance and commitment therapy; CBT: cognitive behavioral therapy; EMA: ecological momentary assessment.

We will test tailored smartphone-based messages to support young adults with quitting smoking (study overview in Figure 1). Our study addresses 3 specific aims. For Aim 1, an MRT (within-subject randomization) with 80 young adult smokers will investigate the efficacy of smoking cessation messages based on CBT and mindfulness or ACT for reducing smoking urge 15 minutes after message delivery. In Aim 2, a built-in and conventionally randomized EMA-only control group will allow us to test if intervention messages result in changes in smoking behavior over time. The primary outcome will be self-reported number of cigarettes per day at the end of treatment, as well as at 3-month and 6-month follow-ups. Aim 3 will explore moderation effects of substance co-use (cannabis, alcohol, other drugs) and exposure to specific locations (home, work, bars) on urge reduction message efficacy among intervention group participants.

### Inclusion Criteria

Participants will be young adults who (1) live in the United States, (2) read English, (3) are between 18 years and 30 years of age, (4) own a smartphone with iOS or the Android operating system, (5) have smoked ≥100 cigarettes in their lives and currently smoke at least 3 cigarettes per day on 5 or more days of the week, and (6) are planning to quit smoking within the next 30 days. There are no exclusion criteria for this study.

### Recruitment

Participants will be recruited through social media advertisements, including Facebook, Instagram, Reddit, X (formerly Twitter), LinkedIn, and TikTok, a design and targeting strategy that we have previously used for recruiting diverse young adult smokers for EMA studies [18,41] and smoking cessation studies delivered through social media [35,42-44]. Additional recruitment efforts will use ResearchMatch, a nonprofit program funded by the National Institutes of Health that allows researchers from US institutions to reach out to and recruit volunteers interested in research studies. Finally, recruitment will be conducted through MyChart messages at Johns Hopkins. Eligible patients will be identified via electronic health records and will receive direct invitations through MyChart, the institution’s patient portal.

### Ethical Considerations

#### Human Subject Ethics Review Approvals

All study procedures are reviewed and approved by the institutional review board of the Johns Hopkins Bloomberg School of Public Health (IRB00013413).

#### Informed Consent

All participants will be required to meet eligibility criteria and provide online consent prior to study involvement. To assess understanding of the information provided in the informed consent, eligible participants will be asked a series of 3 multiple-choice questions regarding the informed consent before being able to proceed with the study. Answers will be collected, and any wrong answer will result in the potential participant being prompted to review the full consent document before attempting the consent questions again. Potential participants will not be enrolled in the study if they answer the questions incorrectly 4 times. After consenting to participate but before being enrolled into the study, participants will be required to send study staff a picture of a valid identification (eg, driver’s license) that has their name, picture, and birth date to validate their age and the fact that they are a real person. Only the participant picture on the ID (headshot) will be retained for biochemical verification of smoking abstinence and will be deleted at the end of data collection.

#### Privacy and Confidentiality

Participant privacy and data confidentiality will be maintained throughout the study. Screening, baseline, and follow-up survey data will be collected using the Qualtrics survey platform, and data are securely stored on Johns Hopkins servers. Moreover, the study utilizes a HIPAA-compliant mobile research platform (MetricWire Catalyst) to administer EMA surveys, capture GPS location data, and deliver intervention messages. Data collected through the app are encrypted during transmission and securely stored on the platform’s servers. No personally identifiable information is stored on the participant’s device. All location data are time-stamped and linked only to unique participant identifiers. Access to raw data is limited to authorized study staff. Data used for analysis are de-identified and stored on secure, access-controlled servers at Johns Hopkins. Any publications or presentations resulting from the study will use only aggregated or anonymized data. For example, location data points may be jittered or mapped to an alternative base layer to maintain participant anonymity.

#### Compensation Details

The study will use an incentive scheme to reward participants for high compliance with EMA prompts. Participants will receive US $2 for each day of participation in the EMA surveys (US $2 x 44 days = US $88) plus an extra incentive of US $90 if they complete at least 75% of the prompted assessments. Participants will also receive additional incentives for completion of baseline (US $10) and follow-up surveys at 3 months and 6 months (US $20 each). The maximum total incentive amount will be US $228 for participants in the trial.

### Study Procedure

#### Randomization

We will use a dynamic minimization randomization approach to assign participants into the MRT intervention group (n=80) and EMA-only control group (n=80; Figure 1) while ensuring balance across arms on gender and nicotine dependence status. The variable known to robustly impact smoking cessation success is level of nicotine dependence (smoking within first 30 minutes after waking: yes/no) [45]. Gender will be used as additional variable to be balanced across arms. This adaptive approach recalculates imbalance scores in real time based on the distribution of previously assigned participants, assigning participants to the group that minimizes overall imbalance. Adaptive randomization using minimization will enable real-time assignment while ensuring balanced allocation of participants by key variables that may impact intervention outcomes.

#### Technology Implementation

For technology implementation, we will work with the Canadian company MetricWire and their Catalyst system. This company has developed a technology platform to develop, deploy, and conduct MRTs using an app on participants’ own devices. MetricWire is HIPAA compliant, thus minimizing participant risk for loss of privacy. Further, we have successfully used their system in our previous EMA studies [46-52].

#### Intervention Messages

**Figure 2:**
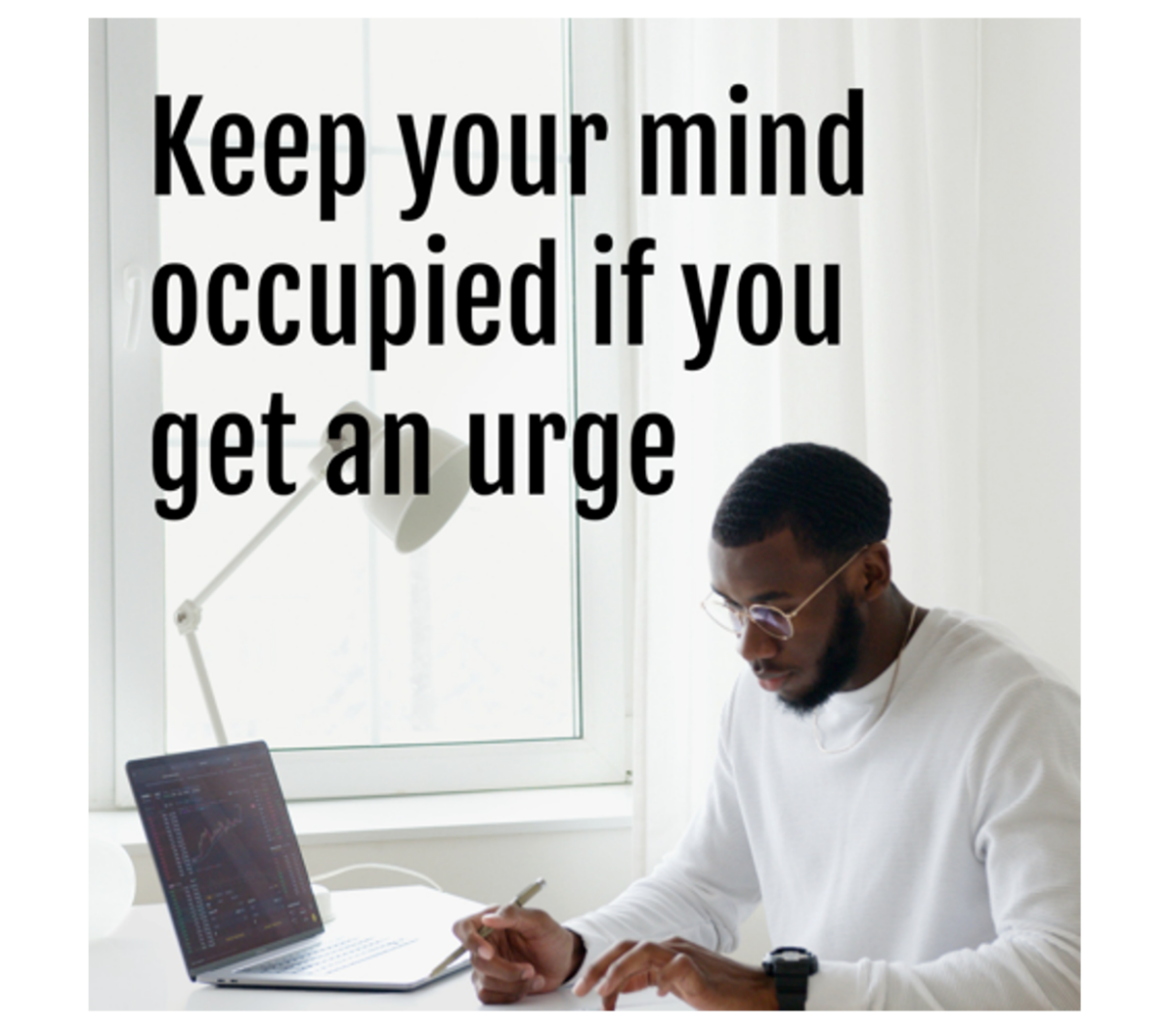
Intervention message examples for distraction and acceptance messages, including image and text content for a cognitive behavioral therapy (CBT) distraction message: "Keep your mind occupied if you get an urge. Do a mental puzzle, make your next shopping list, read a book, write a poem...you might be surprised how well occupying your mind can get rid of urges.".

**Figure 3:**
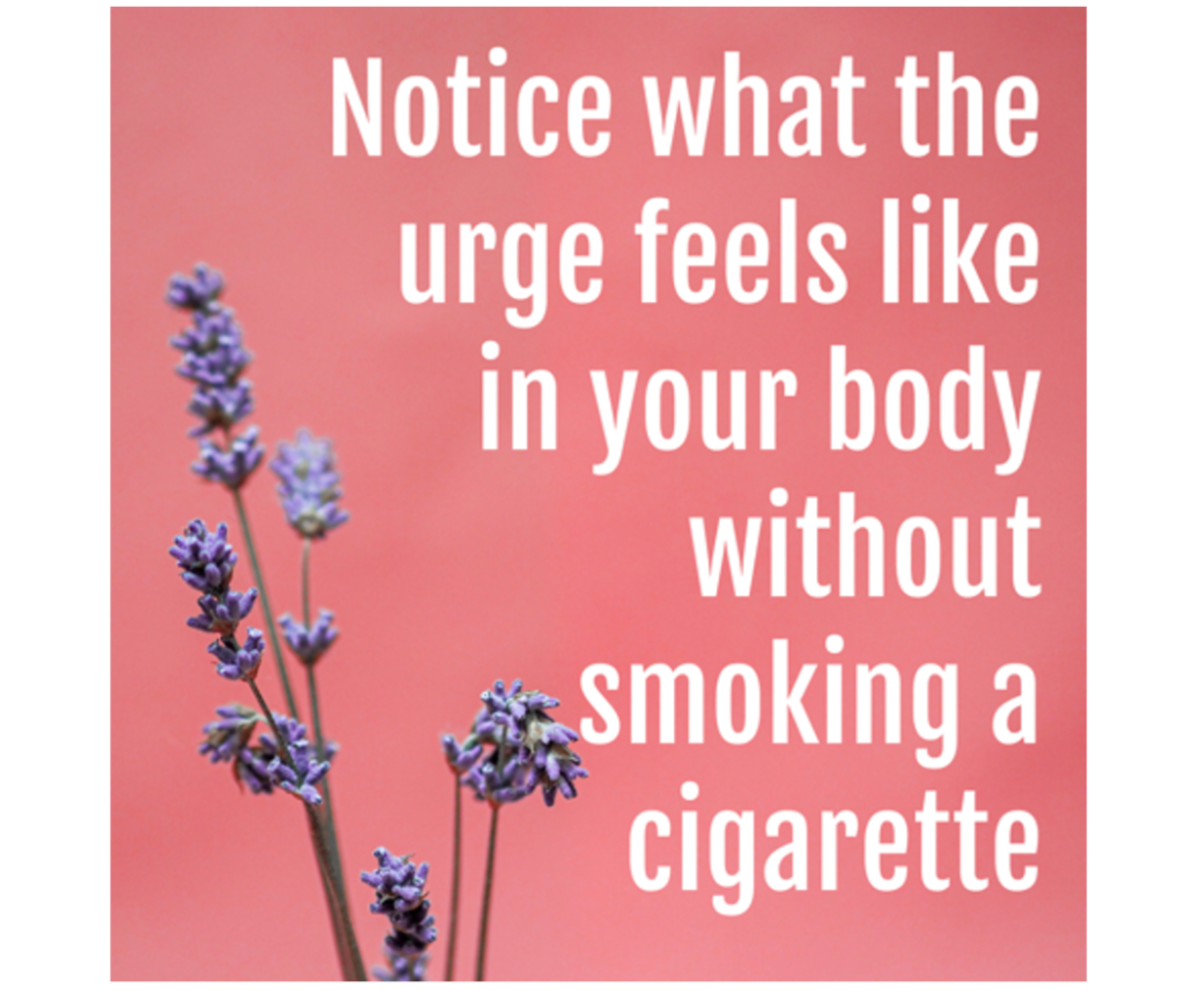
Intervention message examples for distraction and acceptance messages, including image and text content for an acceptance and commitment therapy (ACT) acceptance message: "Take a minute to notice what the urge feels like in your body without smoking a cigarette. Notice what is going on. See what happens. Does the urge pass?".

Intervention messages came from several previous studies [33,53,54]. Initially, these intervention messages were refined internally and combined with image content from free stock photo websites (Pexels, Unsplash). See Figures 2 and 3 for intervention message examples. Before inclusion in the trial, a total of 124 intervention messages were rated by an online Qualtrics panel of 301 diverse young adults (18-30 years old) who endorsed current cigarette smoking. Each participant rated 10 randomly selected messages (3010 total message ratings; 24.3 ratings per message) on dimensions of content, design, helpfulness for in-the-moment urge reduction, and helpfulness for quitting smoking [55]. Of these 124 messages, the 34 messages with lowest average scores on dimensions of helpfulness for in-the-moment urge reduction and helpfulness for quitting smoking were dropped, for a total of 90 messages that were retained and used in this trial. All intervention messages are available on the project Open Science Framework page [56] and in Multimedia Appendix 1.

#### Participant Compliance and Incentives

Frequent assessments may lead to participant noncompliance and study attrition. Procedures are built into the study to prevent attrition over time: The app tracks participant compliance and provides feedback to participants. We will monitor compliance over time; proactively reach out to participants via calls, text messages, and email to keep them engaged; and increase contact with participants if their compliance drops below the target of a 75% response rate. We will explore potential participant problems and remind them of the importance of compliance for data quality and of the extra incentive for high compliance. Compensation details are provided in the Ethical Considerations section of the manuscript.

### Treatment Conditions

#### Training Phase—EMA Data Collection

##### Baseline Survey

Initially, all participants will complete a baseline survey on the online survey platform Qualtrics to assess basic demographics; smoking and other tobacco use behavior, including e-cigarettes, nicotine dependence [57], quitting history and current quit motivation, and frequency and intensity of smoking urges; and other substance use behavior including alcohol and cannabis. Smoking-specific experiential avoidance, a measure to assess a person’s willingness to experience negative mental states (eg, urges, mood, thoughts), will be assessed with the Avoidance and Inflexibility Scale [58,59]. Psychological flexibility will be assessed with the CompACT-15 [60]. Baseline measures will also include psychological distress [61].

##### EMA Data Collection

Before EMA data collection, participants will be contacted by phone to receive detailed instructions on how to use the EMA study app. Participants will use their own smartphones and the study app to collect data on smoking situations over the course of 14 days (EMAs of smoking situations and smartphone location sensor data). Participants will complete 3 randomly prompted EMA surveys per day and will report every time they smoke a cigarette. A random subset of these cigarette reports will trigger up to 3 EMA smoking survey prompts per day. All EMA surveys and codebooks are provided in Multimedia Appendices 2 and 3. 

##### EMA Momentary Surveys

Smoking urges will be recorded with a single item in accordance with recommendations from the Society for Research on Nicotine and Tobacco. Additional questions will examine internal and external aspects of the situation. The number of questions asked will be limited to ensure they do not interfere with participants’ daily activities. In addition, the EMA software will log participants’ geolocation based on GPS. All data will be time- and date-stamped to allow time-specific analyses and determine high-risk periods for smoking.

##### EMA Daily Diaries

Thorough daily diaries will be collected to assess overall cigarette, other tobacco product (including e-cigarettes), alcohol and cannabis. Questions will also assess same occasion co-use of tobacco, alcohol, cannabis, and drugs (when participants were feeling under the influence of other substance).

##### Purpose of the EMA Training Phase

The EMA training phase serves multiple purposes: First, it allows participants to get used to the study app that will also deliver the intervention messages, and second, the collected data will allow us to generate an individual risk profile with regard to time of day and location (by combining time stamps, GPS data, and self-reported data) with the highest likelihood of smoking for each participant. We will use geofencing to generate geospatial buffers around these high-risk locations. In combination with time-of-day information to target high-risk time periods for smoking, these geofences will trigger delivery of intervention messages when a mobile device enters the area: A message can be triggered when a participant approaches a smoking location during one of the high-risk time windows. We will use our established protocol for triggering intervention messages that we developed in previous work [32] and successfully used in a pilot trial [36]. Participants will also be prompted to self-report relevant smoking locations in the MetricWire app.

#### Intervention Phase—Microrandomized Trial With 80 Participants to Determine Message Efficacy

**Figure 4:**
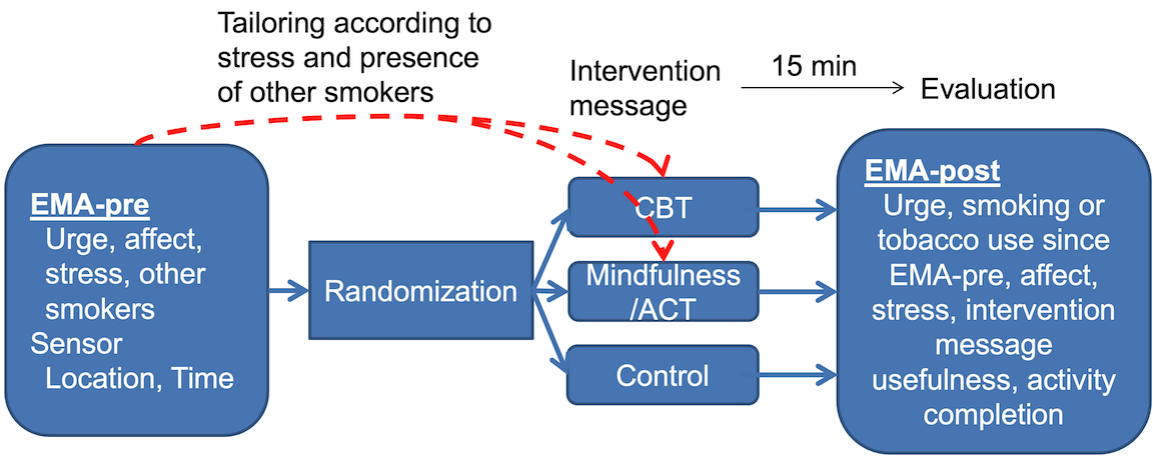
Flowchart for delivery of each intervention message in the microrandomized trial group. ACT: acceptance and commitment therapy; CBT: cognitive behavioral therapy; EMA: ecological momentary assessment.

The MRT will determine if CBT and mindfulness or ACT messages are superior to control messages for reducing the primary outcome momentary smoking urges. Based on participants’ training data collected in the initial 14 days of EMA monitoring, intervention messages will be delivered during time periods and at high-risk locations for smoking. In the intervention phase, participants will be prompted to complete up to 3 geofence-triggered EMAs per day for a total of 30 days. Each EMA will be followed by an intervention message, and the type of message (CBT, mindfulness or ACT, control) will be randomly selected at each time point (within-subject randomization; see Figure 4). Each intervention message will also be tailored to situational factors from the EMA-pre data. Message tailoring will focus on 2 key situational triggers stemming from our conceptual framework: (1) stress (high/low) and (2) presence of other smokers (yes/no). For each situation, characterized by a combination of these 4 possible characteristics, several messages will be placed in separate message bins (4 bins for each CBT and mindfulness or ACT message, for a total of 8 bins). Control messages will thank participants for completing an assessment. Proximate outcomes will be assessed 5 minutes to 15 minutes after message delivery and include urge levels, smoking or other tobacco product use since EMA-pre survey, affect, stress, and an evaluation of the last message (eg, perceived usefulness of message, completion of suggested activity or intervention). In addition, participants will continue completing 1 brief retrospective EMA each morning. Just like the training phase, this retrospective EMA will assess cigarette, other tobacco product, alcohol and cannabis use.

#### Built-in Parallel-Group RCT—EMA-Only Control Group (n=80)

A total of 80 participants will be randomized into an EMA-only control group, parallel to the MRT intervention group (Figure 1). This group will conduct a 14-day EMA-only training phase just like the MRT group but will not be switched over to the intervention phase after these initial 14 days. Instead, participants will continue the EMA-only data collection procedure for an additional 30 days (analogous to the 30-day intervention phase of the MRT). During these 30 days, the EMA-only control group will continue to receive 3 randomly prompted EMA surveys per day and an additional 3 EMA surveys triggered by smoking reports. By comparing this control group with the MRT intervention group, we will investigate if intervention message delivery results in changes in smoking behavior over time (see the Analytical Strategy Aim 2: To Test if Urge Reduction Messages Change Smoking Behavior Over Time section).

### Measures

#### Primary Outcomes

The primary proximal outcome will be change in participants’ ratings of smoking urge in EMA-post surveys, prompted up to 15 minutes after intervention message delivery, and controlling for the ratings in EMA-pre surveys. Urge will be assessed using a single item on a 5-point scale, ranging from 1 (very low) to 5 (very high).

The primary distal outcome will be change in self-reported number of cigarettes smoked per day in the past week from baseline to the 45-day, 3-month, and 6-month follow-ups.

#### Secondary Outcomes

This study will also assess several secondary outcomes. Proximal secondary outcomes will be collected up to 15 minutes after message delivery and include smoking or other tobacco product use (including e-cigarettes), change in rating of stress and negative affect in EMA-post surveys (controlling for the ratings in EMA-pre surveys), ratings of message helpfulness, and self-reported completion of intervention message recommendation. Distal secondary outcomes will be collected at the 45-day, 3-month, and 6-month follow-ups and include self-reported 7-day point prevalence abstinence from smoking, saliva cotinine–confirmed 7-day point prevalence abstinence (assessed using saliva test strips mailed to participants and photo confirmation) [44,62-64], change (reduction) in cigarettes smoked by at least 50% between baseline and each follow-up time point, tobacco quit attempts, change in frequency and intensity of smoking urges as assessed using 2 items of the Mood and Physical Symptoms Scale [65], change in psychological flexibility as assessed using the CompACT-15 [60], change in smoking-specific experiential avoidance as assessed with the Avoidance and Inflexibility Scale [58,59], and switching to other tobacco products including e-cigarettes from baseline to follow-up.

### Power Calculation

#### Aim 1

A sample size of 80 participants receiving 3 randomized messages per day for 30 days will provide 7200 observations. Across person-days, there will be an average of 1 prompt per day for each of the 3 conditions: CBT, mindfulness or ACT, and control (balance across person-days). Power estimations were conducted using an online tool [66] specifically developed for sample size calculations for MRTs [37,67]. Assuming a message randomization probability of 1/3 (33% CBT, 33% mindfulness or ACT, 33% control), a quadratic effect over time (initially smaller as participants are getting used to the intervention, increasing over time to peak at day 20, and decreasing thereafter as participants may get desensitized) [68], and an average compliance rate of 75% linearly decreasing over time, a sample size of 80 participants will allow for detecting a standardized mean difference between CBT, mindfulness or ACT, and control conditions on the primary outcome of self-reported smoking urge of *d*=0.1 with a power of 0.8 and an α level of .05. This measure of effect size is a generalization of Cohen *d* (standardized mean difference), with the difference that the standardization is by the average standard error over the entire study, with multiple treatments for each person. A Cohen *d* of 0.1 is considered a small effect. A previous EMA study found that playing Tetris (analogous to a CBT distraction technique) decreased substance use urges with a mean effect size of *f*^2^=0.12 (medium-sized effect) [69]. Given these effect sizes in the existing literature, we will be adequately powered to detect effects in Aim 1 analyses.

#### Aim 2

This aim will test between-group differences in number of cigarettes smoked over time among MRT (n=80) and EMA-only control group (n=80) participants. Sample size calculations were based on findings from an app-based mindfulness meditation trial for smoking reduction. This trial only delivered intervention content for 14 days (as opposed to the 30 days in this study) and found a significant reduction in 3.8 cigarettes per day in the intervention group compared with an increase in 0.8 cigarettes per day in the control group [70], which translates to an effect size of *d*=0.651 (*f*=0.326) [71]. Power estimation for between-group repeated-measures ANOVA and interactions were conducted with G-Power. Assuming an α level of .05, a sample size of 160 will allow us to detect effects in reduction of cigarettes per day of *f*=0.326 and larger with a power of 0.80. Multiple imputation will be used for missing data, and the full sample will be analyzed.

### Data Analysis

#### Analytical Strategy Aim 1: To Compare CBT and ACT Intervention Message Efficacy

Aim 1 analyses to test the hypothesis of a proximal benefit of urge reduction messages will be conducted using a centered and weighted least squares method [72], which estimates treatment effects and allows inclusion of covariates. The method is similar to generalized estimating equations (GEEs) [73] and multilevel models in that it accounts for dependence of responses within individuals due to repeated measures via the use of robust standard errors. Moreover, the method takes advantage of sequential randomization to estimate causal treatment effects. The primary outcome will be participants’ ratings of smoking urge in EMA-post surveys, prompted up to 15 minutes after intervention message delivery and controlling for the ratings in EMA-pre surveys. The main independent variable will be message type: CBT versus mindfulness or ACT versus a control message (“Thank you for completing the survey”). We will also test differences in efficacy between CBT versus mindfulness or ACT. We will use multiple imputation to impute missing data in EMA-post surveys.

Secondary outcomes from EMA-pre to EMA-post surveys to be investigated include smoking in the 15-minute time window since message delivery, other tobacco product use (including e-cigarettes), as well as participant ratings of negative affect, stress, perceived message usefulness, and activity completion. The centered and weighted least squares method with robust standard errors [72] will be used to adjust for multiple observations nested within participants. The number of cigarettes per day and other tobacco product use (including e-cigarettes) as reported in the daily diary EMA will be analyzed as additional secondary outcome to investigate participants’ changes in smoking and other tobacco use over time while enrolled in the MRT. As poly- and multiple tobacco product use is increasingly common among young adults, we will be able to investigate if participants are switching from cigarettes to other tobacco products (including e-cigarettes) over time.

Among MRT participants, we will explore changes in message efficacy on smoking urges over time by investigating both overall effects (across all time points) as well as linear and quadratic time trend of the intervention effect, assessed by estimating the interaction between treatment effect and day in the study with the weighted least squares method [72]. These models assess whether the relationship between a predictor and outcome changes over time. Separately for CBT and mindfulness or ACT messages, we will explore whether EMA-pre and EMA-post urge reduction changes as the intervention progresses. The same analyses will also be conducted for secondary outcomes. Analyses will provide evidence for increasing or diminishing returns of message delivery with time. Sex as a biological variable will be considered as a participant-level covariate in all models. We will also control for nicotine dependence, psychological distress, and trait negative affect.

#### Analytical Strategy Aim 2: To Test if Urge Reduction Messages Change Smoking Behavior Over Time

Aim 2 analyses will use data from both the MRT group (n=80) and the EMA control group (n=80). The primary outcome will be the self-reported number of cigarettes per day in the past week at the 45-day, 3-month, and 6-month follow-ups. GEEs [73] will examine cigarettes per day at each follow-up by group (MRT vs EMA-only control). Independent variables are group membership, variables that differ by group at baseline, gender, and nicotine dependence. Our previous work has shown follow-up completion rates of up to 82% of participants at the 6-month follow-up in a Facebook smoking cessation trial with young adults [43]. Again, multiple imputation procedures will be used to impute missing data at follow-up.

To compare if delivery of CBT or mindfulness or ACT messages is associated with smoking outcomes over time, we will conduct dose-response post hoc analyses. A total of 90 message randomizations per participant will result in a range of empirical distributions of message type dose. We conducted a simulation of 90 randomizations over 80 participants in R, which resulted in a range of between 18 and 42 messages of CBT or ACT or mindfulness across participants for the intervention duration. Post hoc analyses will investigate if the extent of exposure to CBT or mindfulness or ACT messages predicts smoking outcomes. These analyses will control for baseline nicotine dependence, quit motivation, and total exposure to intervention messages.

For the secondary outcomes, we will run GEE models and mixed effects multinomial logistic regression analyses to analyze longitudinal ordinal response data to model the following outcomes across time (45 days, 3 months, 6 months): (1) self-reported 7-day point prevalence abstinence (yes/no—with sensitivity analyses for missing=smoking, complete cases, and multiple imputation), (2) saliva cotinine–confirmed 7-day point prevalence abstinence (yes/no—again with sensitivity analyses for different assumptions including self-reported nicotine replacement or other tobacco product use), (3) reduction of cigarettes by 50% or more (yes/no), (4) tobacco quit attempt (yes/no), (5) switching to other tobacco products including e-cigarettes, (6) frequency and intensity of smoking urges, and (7) ACT measures of experiential avoidance and mindfulness. Independent variables are group, gender, nicotine dependence, and any variables that differ by group at baseline.

#### Analytical Strategy Aim 3: Explore Moderation Effects of Substance Co-Use (Cannabis, Alcohol, Other Drugs) and Exposure to Specific Location (Home, Work, Bars) on Urge Reduction Message Efficacy

Based on our data on high co-use of cigarettes with cannabis and alcohol [74-76], as well as changes in perceived reward of smoking cigarettes when under the influence of cannabis or alcohol [77-79], analyses in this aim will explore if message efficacy is moderated by substance co-use (whether a participant currently is under the influence of another substance [eg, cannabis, alcohol, other drugs]). Centered and weighted least squares models [72] similar to those for Aim 1 will be estimated with predictors of intervention message type (CBT, mindfulness or ACT, control), substance use (cannabis, alcohol, other drugs, none), and their interaction. Based on existing evidence on alcohol and cannabis co-use as barrier to cigarette smoking cessation, we hypothesize that intervention message efficacy will be reduced when participants are under the influence of another substance. However, analyses for this aim will enable us to explore if specific types of messages (CBT, mindfulness or ACT) are more helpful in co-use situations.

Moreover, we will explore aspects of intervention message-situation fit. We will test if specific locations (eg, home, work, bars) impact intervention message efficacy. Mixed models will be estimated containing the predictors of intervention type (CBT, mindfulness or ACT, control), location (home, work, bar, other locations), and their interaction. We will explore other situational characteristics and intervention message-situation fit based on affect, arousal, stress, and the presence of other smokers. We do not have specific hypotheses about moderation effects of location and other situational characteristics, but findings will inform future interventions using adaptive messages over time to improve intervention message-situation fit and intervention efficacy.

## Results

As of June 2025, we had enrolled 58 participants of the target sample of 160, with 52% (30/58) assigned to the MRT group and 48% (28/58) assigned to the EMA-only control.

## Discussion

### Principal Findings

This is one of the first studies that combines an MRT [30,31] using within-subject randomization and a conventional between-subject RCT design to test the real-time impact of smartphone-based intervention messages on young adult cigarette smoking outcomes. In a traditional RCT, baseline randomization does not offer protection against causal confounding due to time-varying factors and contexts in which intervention messages are delivered. Individual participants self-select into unique high-risk situations, which are impossible to control for and balance across groups in a 3-arm RCT. In an MRT, multiple within-subject randomizations will produce compositional balance in unobserved factors between the message conditions and balance of different intervention messages across participants, days, and situations. Because messages are repeatedly randomized, resulting data will allow us to assess how causal effects of messages change over time. Moreover, between-person randomized designs do not allow investigation of message-situation fit and how time-varying factors may moderate intervention effects (Aim 3). Even within participants, CBT or mindfulness or ACT messages may be more effective in different situations depending on contextual factors and between-subject randomization and assignment to exclusive CBT and mindfulness or ACT conditions would not allow us to investigate this. For these reasons, microrandomization of messages is critical for informing the development of an adaptive smoking cessation intervention. Last, MRTs are highly efficient. Due to repeated randomization within participants, effect estimations can take advantage of between- and within-subject contrasts. These within-subject comparisons allow our MRT to recruit far fewer participants than a traditional full factorial design [30]. Baseline randomization into a MRT group or control group is important to test whether repeated delivery of intervention messages improves smoking behavior outcomes over and above just repeated assessment of smoking situations over an extended period of time. To provide evidence for the long-term efficacy of CBT or mindfulness or ACT messages on smoking behavior, we will conduct post hoc dose-response analyses in Aim 2.

### Limitations

Despite increasing trends in the use of novel tobacco products, including e-cigarettes, in recent years, tobacco cigarettes are still the most frequently used tobacco product. However, in a changing tobacco product landscape [80] and because of high rates of polytobacco use among young adults [6], it is important to assess the full spectrum of tobacco product use among young adults over time, to investigate if participants are switching from cigarettes to other tobacco products. Message refinement will ensure intervention messages are worded so they will be applicable to tobacco use urges beyond cigarettes. If we find in this MRT that urge reduction messages are effective for cigarette smoking urges, results could inform future intervention trials to counter urges to use other tobacco products.

In the past, we have successfully recruited young adults ready to quit in the next 30 days for smoking cessation interventions [42]. If we find that the recruitment on social media will not result in enrollment of adequate participant numbers, we will pursue additional strategies to boost recruitment, which include outreach to local schools and colleges and flyers at places young adults purchase tobacco.

Frequent assessments may lead to participant noncompliance and study attrition. Participants will be asked to complete up to 6 EMAs per day. A recent EMA study has shown that participants were equally responsive when they had to complete 1 versus 6 EMAs per day [81], suggesting that this is a feasible EMA frequency. Moreover, EMA survey length, compared with frequency, seems to have a greater impact on participant compliance [82], which means we will keep EMA surveys brief. Additional measures are built into the study to prevent attrition over time, including compliance feedback, outreach to participants, and an incentive scheme designed to promote high compliance.

### Conclusions

We are testing the use of mobile technology for cancer control and prevention in an underserved, high-priority population. In the current situation of rapidly changing technology platforms as well as changes in the tobacco product landscape, it is important to conduct research that is not uniquely tied to a specific platform and tobacco product. This research builds on existing infrastructure and does not aim at developing a new smoking cessation app. Instead, results of this project will provide evidence on the efficacy of tailored intervention messages to help young adult smokers cope with smoking urges as an integral part of smartphone interventions. Findings will inform the field about key principles, strategies, and efficacy of situational tailoring of app-based tobacco use urge reduction messages. Moreover, this study will produce a database of evidence-based smoking urge reduction messages for real-time smartphone-based interventions. The logical next step after this trial is to use these messages and the results to develop and test a just-in-time adaptive intervention (JITAI) to improve tobacco product cessation among young adults. A JITAI is an intervention design that aims at providing the right type and amount of support, at the right time, by adapting to an individual’s changing internal and contextual state [83,84]. This MRT will provide the necessary evidence and intervention content to inform a JITAI to support young adult tobacco use cessation.

## Appendix

Multimedia Appendix 1 Intervention messages.

Multimedia Appendix 2 EMA surveys.

Multimedia Appendix 3 EMA survey codebook.

Multimedia Appendix 4 Peer-review report from ZRG1 RPHB-V (02) - Center for Scientific Review Special Emphasis Panel Risk, Prevention and Health Behavior Integrated Review Group, National Cancer Institute and the National Institute on Drug Abuse (National Institutes of Health, USA).

## Abbreviations

ACT: acceptance and commitment therapy
CBT: cognitive behavioral therapy
EMA: ecological momentary assessment
GEE: generalized estimating equations
HIPAA: Health Insurance Portability and Accountability Act
JITAI: just-in-time adaptive intervention
MRT: microrandomized trial
RCT: randomized controlled trial

## Data Availability

All intervention messages and study ecological momentary assessments (EMA) surveys are made available on the project Open Science Framework page [56].
