# Supplementary material for: App-Based Smoking Urge Reduction Intervention for Young Adults: Protocol Combining a Microrandomized Trial and Conventional Between-Subject Randomized Trial
Source: JMIR Res Protoc. 2025 Sep 23;14:e74388. doi: 10.2196/74388 (PMC12504902; doi:10.2196/74388)
Supplement: Multimedia Appendix 2 [file resprot_v14i1e74388_app2.docx]

Table of Contents

[A. Study details 1](#_Toc167998580)

[B. Cigarette smoking self-report (log cigarette) 2](#_Toc167998581)

[C. Other tobacco/nicotine self-report (log other tobacco) 2](#_Toc167998582)

[D. Smoking location self-report (log smoking locations) 2](#_Toc167998583)

[E. Cigarette follow-up survey 3](#_Toc167998584)

[F. Daily diary 7](#_Toc167998585)

[G. Random assessment 12](#_Toc167998586)

[H. Location assessment 15](#_Toc167998587)

[I. Location assessment follow-up 19](#_Toc167998588)

# Study details

- Study groups: intervention group and control group
- Study duration: 45 days
- Training phase: 14 days (day 1 to 14)
- Geofences: created on day 14
- Intervention phase: 30 days (days 15 to 44)
- Day 45: daily diary only
- Miscellaneous
  - all participants start with a compliance rate of 100% - 0 submissions / 0 possible submissions
  - compliance rate is based on daily diaries, random assessments, cigarette follow-up surveys, location assessment follow-up surveys
  - compliance rate does not include location assessments
  - study day on app insights page transitions to next day at 8:00pm

# Cigarette smoking self-report (log cigarette)

*NOTE: Right before a participant smokes a cigarette, they are directed to open the app and press the log cigarette button. Reporting a cigarette will trigger the cigarette follow-up survey. This button is available for the entire study.*

|  | I’m about to smoke a cigarette. | 1\| Confirm | cigjustnow |
| --- | --- | --- | --- |

# Other tobacco/nicotine self-report (log other tobacco)

*NOTE: Right before a participant uses other tobacco/nicotine products, they are directed to open the app and press the log other tobacco button. This button is available for the entire study.*

|  | I’m about to use one of the following. | 1\|Cigarillo  2\|Dip, Snus, or other smokeless  3\|Hookah  4\|E-cigarette, nicotine vape  5\|Other tobacco product [This will trigger next question, otherwise skip] | tobaccojustnow |
| --- | --- | --- | --- |
|  | What kind of tobacco product? Please specify. |  | otherjustnow |

# Smoking location self-report (log smoking locations)

*NOTE: Participants will be asked to report up to 10 usual smoking locations. This button is available for the entire training phase.*

|  | We are interested in locations where you usually smoke cigarettes. How many smoking locations would you like to log? | 1\|1  2\|  3\|  4\|  5\|  6\|  7\|  8\|  9\|  10\|10 | smoking_loc |
| --- | --- | --- | --- |
|  | Smoking location 1: Please enter a location where you usually smoke cigarettes. | 1\|Geolocation | smoking_coord_loc1 |
|  | Smoking location 1: Please enter the time of day when you usually smoke cigarettes at this location. | 1\|HH:MM | smoking_time_loc1 |
|  | Where is smoking location 1? | 1\|Home  2\|Workplace/School  3\|Other’s home  4\|Bar  5\|Restaurant  6\|Walking between places  7\|Park  8\|Other location | smoking_place_loc1 |

# Cigarette follow-up survey

*NOTE: Right after reporting a cigarette, the platform will trigger this survey based on random probability. The probability is based on the number of cigarettes smoked at baseline (e.g., if a participant smokes 10 cigarettes per day, then the probability that the survey will fire is set to 30%). The app in real time will generate a threshold value and if this threshold value is greater than the probability value, then the survey does not trigger. If the survey does not trigger, this survey is designated as randomized out. If the threshold value is less than the probability value, then the survey will trigger. The platform can trigger this survey a maximum of 3 times per day. Once this survey is triggered, it is available for 60 minutes and the app will send a reminder prompt at the 20- and 40-minute mark.*

*Intervention group: this survey is deactivated and is no longer available once participants enter the intervention phase.*

*Control group: this survey can trigger for the entire study duration.*

|  | JUST BEFORE SMOKING: Overall feeling? | 1\|1 – Very unpleasant  2\|  3\|  4\|  5\|5 – Very pleasant | psfeeling |
| --- | --- | --- | --- |
|  | JUST BEFORE SMOKING: Overall arousal/energy level right now? | 1\|1 – Very low  2\|  3\|  4\|  5\|5 – Very high | psarousal |
|  | JUST BEFORE SMOKING: Overall stress level right now? | 1\|1 – Very low  2\|  3\|  4\|  5\|5 – Very high | psstress |
|  | JUST BEFORE SMOKING: Overall anxiety level right now? | 1\|1 – Very low  2\|  3\|  4\|  5\|5 – Very high | psanxiety |
|  | JUST BEFORE SMOKING: Craving a cigarette or tobacco product? | 1\|1 – Very low  2\|  3\|  4\|  5\|5 – Very high | pscraving |
|  | Where were you when you decided to smoke? | 1\|Home  2\|Workplace/School  3\|Other's home  4\|Bar  5\|Restaurant  6\|Vehicle  7\|Walking between places  8\|Public transit stop  9\|Other location [This will trigger next question, otherwise skip] | pslocation |
|  | Location when you decided to smoke? (please specify) |  | pslocationother |
|  | JUST BEFORE SMOKING: Location? Inside/outside. | 1\|Inside  2\|Outside (patio, entrance, street, etc.) | psinsideoutside |
|  | JUST BEFORE SMOKING: Smoking cigarettes allowed? | 1\|Forbidden  2\|Discouraged  3\|Allowed | pscigsallowed |
|  | Did you change location to smoke? | 1\|Yes  0\|No | pschangelocation |
|  | JUST BEFORE SMOKING: With others? | 2\|Friends  3\|Acquaintances  4\|Family members  5\|Coworkers  6\|Romantic partner  7\|Unknown person  1\|Alone [Skip follow up social questions] | pssocial2 |
|  | JUST BEFORE SMOKING: With how many people? | 1\|1  2\|2-4  3\|5-20  4\|21+ | pssocial3 |
|  | JUST BEFORE SMOKING: Other people smoking cigarettes or using tobacco products? | 1\|Yes [This will trigger next two questions, otherwise skip]  0\|No | pssocial4 |
|  | JUST BEFORE SMOKING: Who was smoking cigarettes or using tobacco products? (check all that apply) | 1\|Friends  2\|Acquaintances  3\|Family members  4\|Coworkers  5\|Romantic partner  6\|Unknown person | pssocial5 |
|  | JUST BEFORE SMOKING: How many people were smoking cigarettes or using other tobacco products? | 1\|1  2\|2-4  3\|5-20  4\|21+ | pssocial6 |
|  | JUST BEFORE SMOKING: What were you doing? | 1\|Working/Chores  2\|Inactive/leisure  3\|Interacting with others  4\|Eating/drinking  5\|Between activities  6\|Other activities [This will trigger next question, otherwise skip] | psactivity |
|  | JUST BEFORE SMOKING: What other activities? (please specify) |  | psactivityother |
|  | Consume any of the following in last hour? (check all that apply) | 1\|Food  2\|Caffeinated drink  3\|Non-caffeinated drink  4\|Alcohol  5\|Cigarettes  6\|Other tobacco product  7\|Marijuana or Cannabis  8\|Other substance or drug  9\|None of the above | consume |
|  | Intoxicated and/or drunk? | 1\|1- No!!  2\|  3\|  4\|  5\|5- Yes!! | psintoxicated |
|  | JUST BEFORE SMOKING: Saw any of the following? (check all that apply) | 1\|Cigarette or other tobacco product  2\|Lighter/matches  3\|Cigarette or tobacco product pack  4\|Ashtray  5\|Cigarette or tobacco product in the media  6\|Someone smoking or using tobacco product  7\|Other things related to smoking or tobacco products  8\|None of the above | psexposed |
|  | SINCE THE LAST SURVEY: I felt discriminated against. | 1\|Yes  0\|No [Skip follow up discrimination questions] | discrimination1 |
|  | SINCE THE LAST SURVEY: What was the main reason (s) for the discrimination that you experienced? (check all that apply) | 1\|Your age  2\|Your gender  3\|Your race  4\|Your ethnicity or nationality  5\|Your religion  6\|Your height or weight  7\|Some other aspect of your appearance  8\|A physical disability  9\|Your sexual orientation  10\| Being a smoker  11\|Being poor  12\|Other  13\|None of the above | discrimination2 |
|  | SINCE THE LAST SURVEY: Who discriminated against you? (check all that apply) | 1\|Family member  2\|Boyfriend  3\|Girlfriend  4\|Stranger  5\|Acquaintance  6\|Friend  7\|Employer  8\|Other  9\|None of the above | discrimination3 |

# Daily diary

*NOTE: Starting on day 2, the platform will trigger this survey at 9:00am. Once this survey is triggered, it is available until 9:00pm and will send a reminder prompt at 10:00am and 3:00pm. This survey will trigger for the entire study duration.*

|  | YESTERDAY: How many cigarettes? | 0\| 0 - I did not smoke yesterday (not even a puff)  1\|1  2\|2-5  3\|6-10  4\|11-15  5\|16-20  6\|21-30  7\|31+ | cigsyest1 |
| --- | --- | --- | --- |
|  | YESTERDAY: How many cigarillos? | 0\| 0 - I did not use cigarillos yesterday  1\|1  2\|2-5  3\|6-10  4\|11-15  5\|16-20  6\|21-30  7\|31+ | cigarillosyest1 |
|  | YESTERDAY: Did you use Juul or other pod e-cigarette? | 1\|Yes [This will trigger next question and question 7, otherwise skip]  0\|No | juulyest1 |
|  | YESTERDAY: How many times Juul or other pod e-cigarette? | 1\|1  2\|2-5  3\|6-10  4\|11-15  5\|16-20  6\|21-30  7\|31+ | juulyest2 |
|  | YESTERDAY: Did you use another type of e-cigarette (e.g., vape pen, tank)? | 1\|Yes [This will trigger next question and question 7, otherwise skip]  0\|No | vapeyest1 |
|  | YESTERDAY: How many times other type e-cigarette (e.g., vape pen, tank)? | 1\|1  2\|2-5  3\|6-10  4\|11-15  5\|16-20  6\|21-30  7\|31+ | vapeyest2 |
|  | Did your e-cigarette/vape contain nicotine YESTERDAY? | 1\|Yes  0\|No | vapeyest3 |
|  | YESTERDAY: Did you use any other tobacco or nicotine product? | 1\|Yes [This will trigger next question, otherwise skip]  0\|No | tobaccoyest1 |
|  | Which tobacco or nicotine product? (check all that apply) | 1\|Smokeless tobacco  2\|Hookah  3\|Other [This will trigger next question, otherwise skip] | tobaccoyest2 |
|  | Which tobacco or nicotine product? (please specify) |  | tobaccoyest3 |
|  | YESTERDAY: Did you use a marijuana or cannabis product? | 1\|Yes [This will trigger cannabis follow up questions, otherwise skip]  0\|No | cannabisyest1 |
|  | How many times marijuana or cannabis product? | 1\|1  2\|2  3\|3  4\|4  5\|5  6\|6  7\|7+ | cannabisyest2 |
|  | YESTERDAY: How did you use marijuana or cannabis? (check all that apply) | 1\|Smoked (pipe, joint, blunt, etc.)  2\|Vape (vaporizer)  3\|Edible (candy, cookie, etc.)  4\|Dab or other concentrate  5\|Other | cannabisyest3 |
|  | YESTERDAY: How much marijuana did you personally use? (Do NOT include other forms of cannabis you may use (such as concentrates))  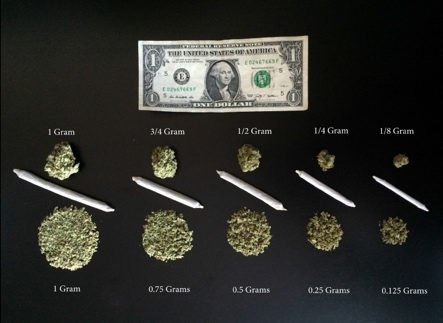 | 1\|0.125 grams or less  2\|0.25 grams  3\|0.5 grams  4\|0.75 grams  5\|1 gram or more | cannabisyest4 |
|  | YESTERDAY: Did you combine tobacco and marijuana or cannabis (e.g., blunt, spliff, mixing and smoking in a pipe or bong)? | 1\|Yes [This will trigger next question, otherwise skip]  0\|No | combineyset1 |
|  | YESTERDAY: How did you combine tobacco and marijuana or cannabis? (check all that apply) | 1\|Blunt  2\|Spliff  3\|Mixing and smoking (e.g., in pipe or bong)  4\|Other | combineyest2 |
|  | YESTERDAY: Did you drink any alcohol? | 1\|Yes [This will trigger next question, otherwise skip]  0\|No | alcoholyest1 |
|  | YESTERDAY: Number of alcoholic drinks?  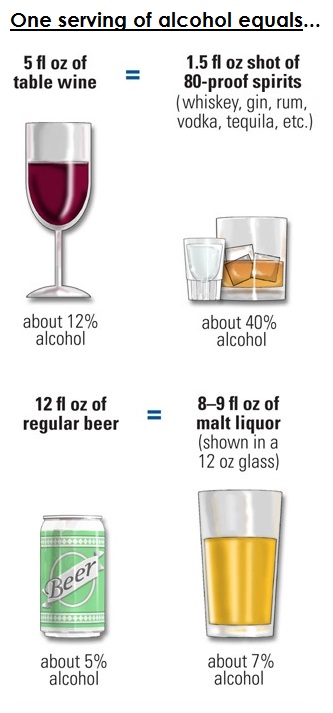 | 1\|1  2\|2  3\|3  4\|4  5\|5  6\|6  7\|7+ | alcoholyest2 |
|  | [If YES to alcoholyest1 AND >0 to cigsyest1]  YESTERDAY: While drinking ALCOHOL, did you also use CIGARETTES? | 1\|Yes  0\|No | combineyest3 |
|  | [If YES to alcoholyest1 AND (YES to juulyest1 OR vapeyest1)]  YESTERDAY: While drinking ALCOHOL, did you also use E-CIGS/VAPES with nicotine? | 1\|Yes  0\|No | combineyest4 |
|  | [If YES to cannabisyest1 AND >0 to cigsyest1]  YESTERDAY: While using CANNABIS, did you also use CIGARETTES? | 1\|Yes  0\|No | combineyest5 |
|  | [If YES to cannabisyest1 AND (YES to juulyest1 OR vapeyest1)]  YESTERDAY: While using CANNABIS, did you also use E-CIGS/VAPES with nicotine? | 1\|Yes  0\|No | combineyest6 |
|  | [If YES to alcoholyest1 AND >0 to cigsyest1]  YESTERDAY: How many of your CIGARETTES did you smoke while drinking ALCOHOL? | 1\|None of them  2\|Some of them  3\|Most of them  4\|All of them | combineyest7 |
|  | [If YES to cannabisyest1 AND >0 to cigsyest1]  YESTERDAY: How many of your CIGARETTES did you smoke while using CANNABIS? | 1\|None of them  2\|Some of them  3\|Most of them  4\|All of them | combineyest8 |
|  | [If YES to alcoholyest1 AND (YES to juulyest1 OR vapeyest1)]  YESTERDAY: How much of your E-CIG/VAPE use with nicotine was while drinking ALCOHOL? | 1\|None of them  2\|Some of them  3\|Most of them  4\|All of them | combineyest9 |
|  | [If YES to cannabisyest1 AND (YES to juulyest1 OR vapeyest1)]  YESTERDAY: How much of your E-CIG/VAPE use with nicotine was while using CANNABIS? | 1\|None of them  2\|Some of them  3\|Most of them  4\|All of them | combineyest10 |
|  | YESTERDAY: I felt discriminated against. | 1\|Yes [This will trigger next two questions, otherwise skip]  0\|No | discriminationyest1 |
|  | YESTERDAY: What was the main reason(s) for the discrimination that you experienced? (check all that apply) | 1\|Your age  2\|Your gender  3\|Your race  4\|Your ethnicity or nationality  5\|Your religion  6\|Your height or weight  7\|Some other aspect of your appearance  8\|A physical disability  9\|Your sexual orientation  10\| Being a smoker  11\|Being poor  12\|Other | discriminationyest2 |
|  | YESTERDAY: Who discriminated against you? check all that apply) | 1\|Family member  2\|Boyfriend  3\|Girlfriend  4\|Stranger  5\|Acquaintance  6\|Friend  7\|Employer  8\|None of the above | discriminationyest3 |

# Random assessment

*NOTE: Starting on day 2, the platform will trigger this survey one time randomly during each 4-hour window (9:00am to 1:00pm, 1:00pm to 5:00pm, 5:00pm to 9:00pm). The platform will trigger this survey a maximum of 3 times per day. Once this survey is triggered, it is available for 60 minutes and will send a reminder prompt at the 20- and 40-minute mark.*

*Intervention group: this survey is deactivated and no longer triggers once they enter the intervention phase.*

*Control group: this survey can trigger for the entire study duration.*

|  | Overall feeling right now? | 1\|1 – Very unpleasant  2\|  3\|  4\|  5\|5 – Very pleasant | feeling |
| --- | --- | --- | --- |
|  | Overall arousal/energy level right now? | 1\|1 – Very low  2\|  3\|  4\|  5\|5 – Very high | arousal |
|  | Overall stress level right now? | 1\|1 – Very low  2\|  3\|  4\|  5\|5 – Very high | stress |
|  | Overall anxiety level right now? | 1\|1 – Very low  2\|  3\|  4\|  5\|5 – Very high | anxiety |
|  | Craving a cigarette or tobacco product? | 1\|1 – Very low  2\|  3\|  4\|  5\|5 – Very high | craving |
|  | Where are you? | 1\|Home  2\|Workplace/School  3\|Other's home  4\|Bar  5\|Restaurant  6\|Vehicle  7\|Walking between places  8\|Public transit stop  9\|Other location [This will trigger next question, otherwise skip] | location |
|  | Which other location? (please specify) |  | locationother |
|  | Location? Inside/outside. | 1\|Inside  2\|Outside (patio, entrance, street, etc.) | insideoutside |
|  | Smoking cigarettes allowed? | 1\|Forbidden  2\|Discouraged  3\|Allowed | cigsallowed |
|  | With others? | 1\|Yes  0\|No [Skip follow up social questions] | social1 |
|  | With others? (check all that apply) | 2\|Friends  3\|Acquaintances  4\|Family members  5\|Coworkers  6\|Romantic partner  7\|Unknown person  1\|Alone | social2 |
|  | With how many people? | 1\|1  2\|2-4  3\|5-20  4\|21+ | social3 |
|  | Other people smoking cigarettes or using other tobacco products? | 1\|Yes [This will trigger next two questions, otherwise skip]  0\|No | social4 |
|  | Who was smoking cigarettes or using other tobacco products? (check all that apply) | 1\|Friends  2\|Acquaintances  3\|Family members  4\|Coworkers  5\|Romantic partner  6\|Unknown person | social5 |
|  | How many people were smoking cigarettes or using other tobacco products? | 1\|1  2\|2-4  3\|5-20  4\|21+ | social6 |
|  | What were you doing? | 1\|Working/Chores  2\|Inactive/leisure  3\|Interacting with others  4\|Eating/drinking  5\|Between activities  6\|Other activities [This will trigger next question, otherwise skip] | activity |
|  | What other activities? (please specify) |  | activityother |
|  | Consume any of the following in last hour? (check all that apply) | 1\|Food  2\|Caffeinated drink  3\|Non-caffeinated drink  4\|Alcohol  5\|Cigarettes  6\|Other tobacco product  7\|Marijuana or Cannabis  8\|Other substance or drug  9\|None of the above | consume |
|  | Intoxicated and/or drunk? | 1\|1- No!!  2\|  3\|  4\|  5\|5- Yes!! | intoxicated |
|  | Saw any of the following? (check all that apply) | 1\|Cigarette or other tobacco product  2\|Lighter/matches  3\|Cigarette or tobacco product pack  4\|Ashtray  5\|Cigarette or tobacco product in the media  6\|Someone smoking or using tobacco product  7\|Other things related to smoking or tobacco products  8\|None of the above | exposed |
|  | SINCE THE LAST SURVEY: I felt discriminated against. | 1\|Yes  0\|No [Skip follow up discrimination questions] | discrimination1 |
|  | SINCE THE LAST SURVEY: What was the main reason (s) for the discrimination that you experienced? (check all that apply) | 1\|Your age  2\|Your gender  3\|Your race  4\|Your ethnicity or nationality  5\|Your religion  6\|Your height or weight  7\|Some other aspect of your appearance  8\|A physical disability  9\|Your sexual orientation  10\| Being a smoker  11\|Being poor  12\|Other  13\|None of the above | discrimination2 |
|  | SINCE THE LAST SURVEY: Who discriminated against you? (check all that apply) | 1\|Family member  2\|Boyfriend  3\|Girlfriend  4\|Stranger  5\|Acquaintance  6\|Friend  7\|Employer  8\|Other  9\|None of the above | discrimination3 |

# Location assessment

*NOTE: This survey will be triggered after recording 5 minutes of passive GPS location data inside a geofence.*

*Geofence location windows:*

*1 9:00:00 to 10:59:59*

*2 11:00:00 to 12:59:59*

*3 13:00:00 to 14:59:59*

*4 15:00:00 to 16:59:59*

*5 17:00:00 to 18:59:59*

*6 19:00:00 to 20:59:59*

|  | Overall feeling right now? | 1\|1 – Very unpleasant  2\|  3\|  4\|  5\|5 – Very pleasant | gf_feeling |
| --- | --- | --- | --- |
|  | Overall arousal/energy level right now? | 1\|1 – Very low  2\|  3\|  4\|  5\|5 – Very high | gf_arousal |
|  | Overall stress level right now? | 1\|1 – Very low  2\|  3\|  4\|  5\|5 – Very high | gf_stress |
|  | Overall anxiety level right now? | 1\|1 – Very low  2\|  3\|  4\|  5\|5 – Very high | gf_anxiety |
|  | Craving a cigarette or tobacco product? | 1\|1 – Very low  2\|  3\|  4\|  5\|5 – Very high | gf_craving |
|  | Where are you? | 1\|Home  2\|Workplace/School  3\|Other's home  4\|Bar  5\|Restaurant  6\|Vehicle  7\|Walking between places  8\|Public transit stop  9\|Other location [This will trigger next question, otherwise skip] | gf_location |
|  | Which other location? (please specify) |  | gf_locationother |
|  | Location? Inside/outside. | 1\|Inside  2\|Outside (patio, entrance, street, etc.) | gf_insideoutside |
|  | Smoking cigarettes allowed? | 1\|Forbidden  2\|Discouraged  3\|Allowed | gf_cigsallowed |
|  | With others? | 1\|Yes  0\|No [Skip follow up social questions] | gf_social1 |
|  | With others? (check all that apply) | 1\|Alone  2\|Friends  3\|Acquaintances  4\|Family members  5\|Coworkers  6\|Romantic partner  7\|Unknown person | gf_social2 |
|  | With how many people? | 1\|1  2\|2-4  3\|5-20  4\|21+ | gf_social3 |
|  | Other people smoking cigarettes or using other tobacco products? | 1\|Yes [This will trigger next two questions, otherwise skip]  0\|No | gf_social4 |
|  | Who was smoking cigarettes or using other tobacco products? (check all that apply) | 1\|Friends  2\|Acquaintances  3\|Family members  4\|Coworkers  5\|Romantic partner  6\|Unknown person | gf_social5 |
|  | How many people were smoking cigarettes or using other tobacco products? | 1\|1  2\|2-4  3\|5-20  4\|21+ | gf_socia6 |
|  | What were you doing? | 1\|Working/Chores  2\|Inactive/leisure  3\|Interacting with others  4\|Eating/drinking  5\|Between activities  6\|Other activities [This will trigger next question, otherwise skip] | gf_activity |
|  | What other activities? (please specify) |  | gf_activityother |
|  | Consume any of the following in last hour? (check all that apply) | 1\|Food  2\|Caffeinated drink  3\|Non-caffeinated drink  4\|Alcohol  5\|Cigarettes  6\|Other tobacco product  7\|Marijuana or Cannabis  8\|Other substance or drug  9\|None of the above | gf_consume |
|  | Intoxicated and/or drunk? | 1\|1- No!!  2\|  3\|  4\|  5\|5- Yes!! | gf_intoxicated |
|  | Saw any of the following? (check all that apply) | 1\|Cigarette or other tobacco product  2\|Lighter/matches  3\|Cigarette or tobacco product pack  4\|Ashtray  5\|Cigarette or tobacco product in the media  6\|Someone smoking or using tobacco product  7\|Other things related to smoking or tobacco products  8\|None of the above | gf_exposed |
|  | SINCE THE LAST SURVEY: I felt discriminated against. | 1\|Yes  0\|No [Skip follow up discrimination questions] | gf_discrimination1 |
|  | SINCE THE LAST SURVEY: What was the main reason (s) for the discrimination that you experienced? (check all that apply) | 1\|Your age  2\|Your gender  3\|Your race  4\|Your ethnicity or nationality  5\|Your religion  6\|Your height or weight  7\|Some other aspect of your appearance  8\|A physical disability  9\|Your sexual orientation  10\| Being a smoker  11\|Being poor  12\|Other  13\|None of the above | gf_discrimination2 |
|  | SINCE THE LAST SURVEY: Who discriminated against you? (check all that apply) | 1\|Family member  2\|Boyfriend  3\|Girlfriend  4\|Stranger  5\|Acquaintance  6\|Friend  7\|Employer  8\|Other  9\|None of the above | gf_discrimination3 |
|  | Intervention message  Step 1: randomly select message group (33% chance)  Step 2: display message based on stress (high/low) and presence of other smokers (yes/no) | 1\|Control message   - Thanks for completing the assessment!   2\|ACT message   - Bin 1 (stress) - Stress ≥ 4 and around other smokers = No - Bin 2 (general) - Stress ≤ 3 and around other smokers = No - Bin 3 (stress/social) - Stress ≥ 4 and around other smokers = Yes - Bin 4 (social) - Stress ≤ 3 and around other smokers = Yes   3\|CBT message   - Bin 1 (stress) - Stress ≥ 4 and around other smokers = No - Bin 2 (general) - Stress ≤ 3 and around other smokers = No - Bin 3 (stress/social) - Stress ≥ 4 and around other smokers = Yes - Bin 4 (social) - Stress ≤ 3 and around other smokers = Yes |  |

# Location assessment follow-up

*NOTE: This survey is triggered randomly between 5 and 15 minutes after completing the location assessment survey. Once this survey is triggered, it is available for 60 minutes and will send a reminder prompt at the 20- and 40-minute mark. Intervention group only.*

|  | Overall feeling right now? | 1\|1 – Very unpleasant  2\|  3\|  4\|  5\|5 – Very pleasant | pgf_feeling |
| --- | --- | --- | --- |
|  | Overall arousal/energy level right now? | 1\|1 – Very low  2\|  3\|  4\|  5\|5 – Very high | pgf_arousal |
|  | Overall stress level right now? | 1\|1 – Very low  2\|  3\|  4\|  5\|5 – Very high | pgf_stress |
|  | Overall anxiety level right now? | 1\|1 – Very low  2\|  3\|  4\|  5\|5 – Very high | pgf_anxiety |
|  | Craving a cigarette or tobacco product? | 1\|1 – Very low  2\|  3\|  4\|  5\|5 – Very high | pgf_craving |
|  | Did you smoke a cigarette since the last intervention message? | 1\|Yes  0\|No | lastsmoke1 |
|  | Did you use another tobacco product since the last intervention message? | 1\|Yes [This will trigger next question, otherwise skip]  0\|No | lasttobacco1 |
|  | What tobacco product? (check all that apply) | 1\|Cigarillo  2\|Dip, Snus, or other smokeless  3\|Hookah  4\|E-cigarette, nicotine vape  5\|Other tobacco product [This will trigger next question, otherwise skip] | lasttobacco2 |
|  | What kind of tobacco product? (please specify) |  | lasttobaccoother |
|  | Did you see an intervention message and picture when you completed the most recent location assessment? | 1\|Yes, I saw a message and a picture  2\|No, there was no message or picture [Skip next 6 questions] | message_displayed |
|  | Do you remember the last intervention message? | 1\|Yes  0\|No | remembermessage |
|  | How would you rate the content (that is, the words and meaning) of the last intervention message? | 1\|Very poor  2\|Poor  3\|Acceptable  4\|Good  5\|Very good | contentrating |
|  | How would you rate the design (that is, how the message looks) of the last intervention message? | 1\|Very poor  2\|Poor  3\|Acceptable  4\|Good  5\|Very good | designrating |
|  | How helpful was the last intervention message for coping with a smoking urge? | 1\|Not at all helpful  2\|Somewhat helpful  3\|Moderately helpful  4\|Very helpful  5\|Extremely helpful | urgehelpful |
|  | How helpful was the last intervention message for supporting you in quitting or reducing smoking? | 1\|Not at all helpful  2\|Somewhat helpful  3\|Moderately helpful  4\|Very helpful  5\|Extremely helpful | supporthelpful |
|  | Did you follow the suggestion of the last intervention message? | 1\|Yes  0\|No | followsuggestion |
