# Supplementary material for: App-Based Smoking Urge Reduction Intervention for Young Adults: Protocol Combining a Microrandomized Trial and Conventional Between-Subject Randomized Trial
Source: JMIR Res Protoc. 2025 Sep 23;14:e74388. doi: 10.2196/74388 (PMC12504902; doi:10.2196/74388)
Supplement: Multimedia Appendix 3 [file resprot_v14i1e74388_app3.pdf]

GeoQuit Codebook

Study Information

Study Name	GeoQuit
Study Description	Thanks for being a part of our study.
Notification Text	GeoQuit has pending questions
Public Enrollment Link	http://my.metricwire.com/studies/info/61af417ccab65ff544a70074

Study System Variables

Participants Invited	0
Participants Enrolled	14
Participants Downloaded App	48
Participants Opted Out	0
Number of Participants Submitting Responses	0

Survey Information

Survey Name / Type	Trigger Name / Type	Questions	Responses
Random Assessment / MOBILE	Number of Triggers: 7	25	0
Location Assessment Follow-Up / MOBILE	Number of Triggers: 3	16	0

Cigarette Follow-Up / MOBILE	Number of Triggers: 21	25	0
Daily Diary / MOBILE	Number of Triggers: 8	34	0
Location Assessment / MOBILE	Number of Triggers: 50	26	0

Survey Information for Random Assessment

- Name: Random Assessment
- Type: MOBILE
- Enable Survey End Screen: No
- Randomized Question Order: No
- Disable Review Question Screen: Yes
- Randomly Trigger a Single Group: No
- Number of Survey Triggers: 7
- Number of Questions: 25

Data Format

Variable Name	Format	Position	Description
Response ID	Character	1	Unique Response Identifier
User ID	Character	2	Unique Participant Identifier
Survey Started Date	Character	3	Date participant started to answer the survey
Survey Started Time	Character	4	Time participant started to answer the survey
Survey Submitted Date	Character	5	Date participant submitted survey
Survey Submitted Time	Character	6	Time participant submitted survey

Time Zone	Character	7	Identifies the time zone in UTC of the paricipant at the time of survey submission
Submission Location	Numeric	8	Location of the user at the time of response submission
Device OS	Character	9	Identifies the device operating system at the time of submission
Device OS Version	Character	10	Identifies the version device operating system at the time of submission
App Version	Numeric	11	Identifies the app version downloaded on the participant's device at the time of submission
Trigger Date	Character	12	Identifies the date that the trigger was prompted to participant
Trigger Time	Character	13	Identifies the time that the trigger was prompted to participant
Trigger Type	Character	14	Identifies the type of trigger that was prompted to participant
Trigger Name	Character	15	Identifies the name of trigger that was prompted to participant
Trigger Index	Numeric	16	Identifies the order of the trigger based on the parent prompt that the trigger was based on

Question Level Response Variables

Question	Variable Name	Format	Position	Question Type	Choices: Coded Value

Overall feeling right now?	feeling	Character	17	SINGLE_CHOICE	- 1 - Very unpleasant : 1<br>- 2 : 2<br>- 3 : 3<br>- 4 : 4<br>- 5 - Very pleasant : 5
Overall arousal/energy level right now?	arousal	Character	18	SINGLE_CHOICE	- 1 - Very low : 1<br>- 2 : 2<br>- 3 : 3<br>- 4 : 4<br>- 5 - Very high : 5
Overall stress level right now?	stress	Character	19	SINGLE_CHOICE	- 1 - Very low : 1<br>- 2 : 2<br>- 3 : 3<br>- 4 : 4<br>- 5 - Very high : 5
Overall anxiety level right now?	anxiety	Character	20	SINGLE_CHOICE	- 1 - Very low : 1<br>- 2 : 2<br>- 3 : 3<br>- 4 : 4<br>- 5 - Very high : 5
Craving a cigarette or tobacco product?	craving	Character	21	SINGLE_CHOICE	- 1 - Very low : 1<br>- 2 : 2<br>- 3 : 3<br>- 4 : 4<br>- 5 - Very high : 5
Where are you?	location	Character	22	SINGLE_CHOICE	- Home : 1<br>- Workplace/School : 2<br>- Other's home : 3<br>- Bar : 4<br>- Restaurant : 5<br>- Vehicle : 6

					- Walking between places : 7<br>- Public transit stop : 8<br>- Other location : 9
Which other location?	locationother	Character	23	TEXT	
Location? Inside/outside.	insideoutside	Character	24	SINGLE_CHOICE	- Inside : 1<br>- Outside (patio, entrance, street, etc.) : 2
Smoking cigarettes allowed?	cigsallowed	Character	25	SINGLE_CHOICE	- Forbidden : 1<br>- Discouraged : 2<br>- Allowed : 3
With others?	social1	Character	26	SINGLE_CHOICE	- Yes : 1<br>- No : 0
Who are you with?	social2	Character	27	MULTIPLE_CHOICE	- Friends : 2<br>- Acquaintances : 3<br>- Family members : 4<br>- Coworkers : 5<br>- Romantic partner : 6<br>- Unknown person : 7
With how many people?	social3	Character	28	SINGLE_CHOICE	- 1 : 1<br>- 2-4 : 2<br>- 5-20 : 3<br>- 21+ : 4
With how many people?	social3_dropdown	Character	29	DROPDOWN	-<br>1 : 1<br>- 2 : 2<br>- 3 : 3<br>- 4 : 4<br>- 5 : 5<br>- 6 : 6

					- 7 : 7<br>- 8 : 8<br>- 9 : 9<br>- 10 : 10<br>- 11 : 11<br>- 12 : 12<br>- 13 : 13<br>- 14 : 14<br>- 15 : 15<br>- 16 : 16<br>- 17 : 17<br>- 18 : 18<br>- 19 : 19<br>- 20 : 20<br>- 21+ : 21
Other people smoking cigarettes or using other tobacco products?	social4	Character	30	SINGLE_CHOICE	- Yes : 1<br>- No : 0
Who was smoking cigarettes or using other tobacco products?	social5	Character	31	MULTIPLE_CHOIC E	- Friends : 1<br>- Acquaintances : 2<br>- Family members : 3<br>- Coworkers : 4<br>- Romantic partner : 5<br>- Unknown person : 6
How many people were smoking cigarettes or using other tobacco products?	social6	Character	32	SINGLE_CHOICE	- 1 : 1<br>- 2-4 : 2<br>- 5-20 : 3<br>- 21+ : 4
How many people were smoking cigarettes or using other tobacco products?	social6_dropdown	Character	33	DROPDOWN	-<br>1 : 1<br>- 2 : 2<br>- 3 : 3

					- 4 : 4<br>- 5 : 5<br>- 6 : 6<br>- 7 : 7<br>- 8 : 8<br>- 9 : 9<br>- 10 : 10<br>- 11 : 11<br>- 12 : 12<br>- 13 : 13<br>- 14 : 14<br>- 15 : 15<br>- 16 : 16<br>- 17 : 17<br>- 18 : 18<br>- 19 : 19<br>- 20 : 20<br>- 21+ : 21
What were you doing?	activity	Character	34	SINGLE_CHOICE	- Working/Chores : 1<br>- Inactive/leisure : 2<br>- Interacting with others : 3<br>- Eating/drinking : 4<br>- Between activities : 5<br>- Other activities : 6
What other activities?	activityother	Character	35	TEXT	
Consume any of the following in last hour?	consume	Character	36	MULTIPLE_CHOICE	- Food : 1<br>- Caffeinated drink : 2<br>- Non-caffeinated drink : 3<br>- Alcohol : 4<br>- Cigarettes : 5<br>- Other tobacco product : 6<br>- Marijuana or Cannabis : 7

					- Other substance or drug : 8
Intoxicated and/or drunk?	intoxicated	Character	37	SINGLE_CHOICE	- 1- No!! : 1<br>- 2 : 2<br>- 3 : 3<br>- 4 : 4<br>- 5 - Yes!! : 5
Saw any of the following?	exposed	Character	38	MULTIPLE_CHOICE	- Cigarette or other tobacco product : 1<br>- Lighter/matches : 2<br>- Cigarette or tobacco product pack : 3<br>- Ashtray : 4<br>- Cigarette or tobacco product in the media : 5<br>- Someone smoking or using tobacco product : 6<br>- Other things related to smoking or tobacco products : 7
SINCE THE LAST SURVEY: I felt discriminated against.	discrimination1	Character	39	SINGLE_CHOICE	- Yes : 1<br>- No : 0
SINCE THE LAST SURVEY: What was the main reason(s) for the discrimination that you experienced?	discrimination2	Character	40	MULTIPLE_CHOICE	- Your age : 1<br>- Your gender : 2<br>- Your race : 3<br>- Your ethnicity or nationality : 4<br>- Your religion : 5<br>- Your height or weight : 6<br>- Some other aspect of your appearance : 7

					- A physical disability : 8<br>- Your sexual orientation : 9<br>- Being a smoker : 10<br>- Being poor : 11<br>- Other : 12
SINCE THE LAST SURVEY: Who discriminated against you?	discrimination3	Character	41	MULTIPLE_CHOICE	- Family member : 1<br>- Romantic partner : 2<br>- Stranger : 4<br>- Acquaintance : 5<br>- Friend : 6<br>- Employer : 7<br>- Other : 8

Breakdown of Each Question

Question & Settings	Question Type	Variable Name	Global Variable Name
1) Overall feeling right now?	SINGLE_CHOICE	feeling	G:RA:feeling

- Display Conditions: None
- Question Groups: None
- Response Required: Yes
- Randomize Choice: No Choices
- Seconds to Answer: 0
- Seconds Until Next Button Becomes Active: 0

Breakdown of Each Trigger

Name	Type	Settings
1)	ONCE	Runs from 2024-04-08T11:37:00 UNTIL

		2025-04-08T00:00:00.000Z
2) RA (Control) (9:00am-1:00pm)	schedule	false
3) RA (Control) (1:00pm-5:00pm)	schedule	false
4) RA (Control) (5:00pm-9:00pm)	schedule	false
5) RA (Intervention) (9:00am-1:00pm)	schedule	false
6) RA (Intervention) (1:00pm-5:00pm)	schedule	false
7) RA (Intervention) (5:00pm-9:00pm)	schedule	false

Question & Settings	Question Type	Variable Name	Global Variable Name
2) Overall arousal/energy level right now?	SINGLE_CHOICE	arousal	G:RA:arousal

- Display Conditions: None
- Question Groups: None
- Response Required: Yes
- Randomize Choice: No Choices
- Seconds to Answer: 0
- Seconds Until Next Button Becomes Active: 0

Breakdown of Each Trigger

Name	Type	Settings
1)	ONCE	Runs from 2024-04-08T11:37:00 UNTIL 2025-04-08T00:00:00.000Z
2) RA (Control) (9:00am-1:00pm)	schedule	false
3) RA (Control) (1:00pm-5:00pm)	schedule	false
4) RA (Control) (5:00pm-9:00pm)	schedule	false

5) RA (Intervention) (9:00am-1:00pm)	schedule	false
6) RA (Intervention) (1:00pm-5:00pm)	schedule	false
7) RA (Intervention) (5:00pm-9:00pm)	schedule	false

Question & Settings	Question Type	Variable Name	Global Variable Name
3) Overall stress level right now?	SINGLE_CHOICE	stress	G:RA:stress

- Display Conditions: None
- Question Groups: None
- Response Required: Yes
- Randomize Choice: No Choices
- Seconds to Answer: 0
- Seconds Until Next Button Becomes Active: 0

Breakdown of Each Trigger

Name	Type	Settings
1)	ONCE	Runs from 2024-04-08T11:37:00 UNTIL 2025-04-08T00:00:00.000Z
2) RA (Control) (9:00am-1:00pm)	schedule	false
3) RA (Control) (1:00pm-5:00pm)	schedule	false
4) RA (Control) (5:00pm-9:00pm)	schedule	false
5) RA (Intervention) (9:00am-1:00pm)	schedule	false
6) RA (Intervention) (1:00pm-5:00pm)	schedule	false
7) RA (Intervention) (5:00pm-9:00pm)	schedule	false

Question & Settings	Question Type	Variable Name	Global Variable Name

4) Overall anxiety level right now?	SINGLE_CHOICE	anxiety	G:RA:anxiety

- Display Conditions: None
- Question Groups: None
- Response Required: Yes
- Randomize Choice: No Choices
- Seconds to Answer: 0
- Seconds Until Next Button Becomes Active: 0

Breakdown of Each Trigger

Name	Type	Settings
1)	ONCE	Runs from 2024-04-08T11:37:00 UNTIL 2025-04-08T00:00:00.000Z
2) RA (Control) (9:00am-1:00pm)	schedule	false
3) RA (Control) (1:00pm-5:00pm)	schedule	false
4) RA (Control) (5:00pm-9:00pm)	schedule	false
5) RA (Intervention) (9:00am-1:00pm)	schedule	false
6) RA (Intervention) (1:00pm-5:00pm)	schedule	false
7) RA (Intervention) (5:00pm-9:00pm)	schedule	false

Question & Settings	Question Type	Variable Name	Global Variable Name
5) Craving a cigarette or tobacco product?	SINGLE_CHOICE	craving	G:RA:craving

- Display Conditions: None
- Question Groups: None
- Response Required: Yes

- Randomize Choice: No Choices
- Seconds to Answer: 0
- Seconds Until Next Button Becomes Active: 0

Breakdown of Each Trigger

Name	Type	Settings
1)	ONCE	Runs from 2024-04-08T11:37:00 UNTIL 2025-04-08T00:00:00.000Z
2) RA (Control) (9:00am-1:00pm)	schedule	false
3) RA (Control) (1:00pm-5:00pm)	schedule	false
4) RA (Control) (5:00pm-9:00pm)	schedule	false
5) RA (Intervention) (9:00am-1:00pm)	schedule	false
6) RA (Intervention) (1:00pm-5:00pm)	schedule	false
7) RA (Intervention) (5:00pm-9:00pm)	schedule	false

Question & Settings	Question Type	Variable Name	Global Variable Name
6) Where are you?	SINGLE_CHOICE	location	G:RA:location

- Display Conditions: None
- Question Groups: None
- Response Required: Yes
- Randomize Choice: No Choices
- Seconds to Answer: 0
- Seconds Until Next Button Becomes Active: 0

Breakdown of Each Trigger

Name	Type	Settings
1)	ONCE	Runs from 2024-04-08T11:37:00 UNTIL 2025-04-08T00:00:00.000Z
2) RA (Control) (9:00am-1:00pm)	schedule	false
3) RA (Control) (1:00pm-5:00pm)	schedule	false
4) RA (Control) (5:00pm-9:00pm)	schedule	false
5) RA (Intervention) (9:00am-1:00pm)	schedule	false
6) RA (Intervention) (1:00pm-5:00pm)	schedule	false
7) RA (Intervention) (5:00pm-9:00pm)	schedule	false

Question & Settings	Question Type	Variable Name	Global Variable Name
7) Which other location?	TEXT	locationother	G:RA:locationother

- Display Conditions: If Where are you? IS Other location
- Question Groups: None
- Response Required: Yes
- Randomize Choice: No Choices
- Seconds to Answer: 0
- Seconds Until Next Button Becomes Active: 0

Breakdown of Each Trigger

Name	Type	Settings
1)	ONCE	Runs from 2024-04-08T11:37:00 UNTIL 2025-04-08T00:00:00.000Z
2) RA (Control) (9:00am-1:00pm)	schedule	false
3) RA (Control) (1:00pm-5:00pm)	schedule	false

4) RA (Control) (5:00pm-9:00pm)	schedule	false
5) RA (Intervention) (9:00am-1:00pm)	schedule	false
6) RA (Intervention) (1:00pm-5:00pm)	schedule	false
7) RA (Intervention) (5:00pm-9:00pm)	schedule	false

Question & Settings	Question Type	Variable Name	Global Variable Name
8) Location? Inside/outside.	SINGLE_CHOICE	insideoutside	G:RA:insideoutside

- Display Conditions: None
- Question Groups: None
- Response Required: Yes
- Randomize Choice: No Choices
- Seconds to Answer: 0
- Seconds Until Next Button Becomes Active: 0

Breakdown of Each Trigger

Name	Type	Settings
1)	ONCE	Runs from 2024-04-08T11:37:00 UNTIL 2025-04-08T00:00:00.000Z
2) RA (Control) (9:00am-1:00pm)	schedule	false
3) RA (Control) (1:00pm-5:00pm)	schedule	false
4) RA (Control) (5:00pm-9:00pm)	schedule	false
5) RA (Intervention) (9:00am-1:00pm)	schedule	false
6) RA (Intervention) (1:00pm-5:00pm)	schedule	false
7) RA (Intervention) (5:00pm-9:00pm)	schedule	false

Question & Settings	Question Type	Variable Name	Global Variable Name
9) Smoking cigarettes allowed?	SINGLE_CHOICE	cigsallowed	G:RA:cigsallowed

- Display Conditions: None
- Question Groups: None
- Response Required: Yes
- Randomize Choice: No Choices
- Seconds to Answer: 0
- Seconds Until Next Button Becomes Active: 0

Breakdown of Each Trigger

Name	Type	Settings
1)	ONCE	Runs from 2024-04-08T11:37:00 UNTIL 2025-04-08T00:00:00.000Z
2) RA (Control) (9:00am-1:00pm)	schedule	false
3) RA (Control) (1:00pm-5:00pm)	schedule	false
4) RA (Control) (5:00pm-9:00pm)	schedule	false
5) RA (Intervention) (9:00am-1:00pm)	schedule	false
6) RA (Intervention) (1:00pm-5:00pm)	schedule	false
7) RA (Intervention) (5:00pm-9:00pm)	schedule	false

Question & Settings	Question Type	Variable Name	Global Variable Name
10) With others?	SINGLE_CHOICE	social1	G:RA:social1

- Display Conditions: None
- Question Groups: None
- Response Required: Yes

- Randomize Choice: No Choices
- Seconds to Answer: 0
- Seconds Until Next Button Becomes Active: 0

Breakdown of Each Trigger

Name	Type	Settings
1)	ONCE	Runs from 2024-04-08T11:37:00 UNTIL 2025-04-08T00:00:00.000Z
2) RA (Control) (9:00am-1:00pm)	schedule	false
3) RA (Control) (1:00pm-5:00pm)	schedule	false
4) RA (Control) (5:00pm-9:00pm)	schedule	false
5) RA (Intervention) (9:00am-1:00pm)	schedule	false
6) RA (Intervention) (1:00pm-5:00pm)	schedule	false
7) RA (Intervention) (5:00pm-9:00pm)	schedule	false

Question & Settings	Question Type	Variable Name	Global Variable Name
11) Who are you with?	MULTIPLE_CHOICE	social2	G:RA:social2

- Display Conditions: If With others? IS Yes
- Question Groups: None
- Response Required: Yes
- Randomize Choice: No Choices
- Seconds to Answer: 0
- Seconds Until Next Button Becomes Active: 0

Breakdown of Each Trigger

Name	Type	Settings
1)	ONCE	Runs from 2024-04-08T11:37:00 UNTIL 2025-04-08T00:00:00.000Z
2) RA (Control) (9:00am-1:00pm)	schedule	false
3) RA (Control) (1:00pm-5:00pm)	schedule	false
4) RA (Control) (5:00pm-9:00pm)	schedule	false
5) RA (Intervention) (9:00am-1:00pm)	schedule	false
6) RA (Intervention) (1:00pm-5:00pm)	schedule	false
7) RA (Intervention) (5:00pm-9:00pm)	schedule	false

Question & Settings	Question Type	Variable Name	Global Variable Name
12) With how many people?	SINGLE_CHOICE	social3	G:RA:social3

- Display Conditions: ((If Who are you with? IS Friends) OR (If Who are you with? IS Acquaintances) OR (If Who are you with? IS Family members) OR (If Who are you with? IS Coworkers) OR (If Who are you with? IS Romantic partner) OR (If Who are you with? IS Unknown person))
- Question Groups: None
- Response Required: Yes
- Randomize Choice: No Choices
- Seconds to Answer: 0
- Seconds Until Next Button Becomes Active: 0

Breakdown of Each Trigger

Name	Type	Settings
1)	ONCE	Runs from 2024-04-08T11:37:00 UNTIL 2025-04-08T00:00:00.000Z
2) RA (Control) (9:00am-1:00pm)	schedule	false

3) RA (Control) (1:00pm-5:00pm)	schedule	false
4) RA (Control) (5:00pm-9:00pm)	schedule	false
5) RA (Intervention) (9:00am-1:00pm)	schedule	false
6) RA (Intervention) (1:00pm-5:00pm)	schedule	false
7) RA (Intervention) (5:00pm-9:00pm)	schedule	false

Question & Settings	Question Type	Variable Name	Global Variable Name
13) With how many people?	DROPDOWN	social3_dropdown	G:RA:social3_dropdown

- Display Conditions: (If Who are you with? IS Friends) OR (If Who are you with? IS Acquaintances) OR (If Who are you with? IS Family members) OR (If Who are you with? IS Coworkers) OR (If Who are you with? IS Romantic partner) OR (If Who are you with? IS Unknown person)
- Question Groups: None
- Response Required: Yes
- Randomize Choice: No Choices
- Seconds to Answer: 0
- Seconds Until Next Button Becomes Active: 0

Breakdown of Each Trigger

Name	Type	Settings
1)	ONCE	Runs from 2024-04-08T11:37:00 UNTIL 2025-04-08T00:00:00.000Z
2) RA (Control) (9:00am-1:00pm)	schedule	false
3) RA (Control) (1:00pm-5:00pm)	schedule	false
4) RA (Control) (5:00pm-9:00pm)	schedule	false
5) RA (Intervention) (9:00am-1:00pm)	schedule	false

6) RA (Intervention) (1:00pm-5:00pm)	schedule	false
7) RA (Intervention) (5:00pm-9:00pm)	schedule	false

Question & Settings	Question Type	Variable Name	Global Variable Name
14) Other people smoking cigarettes or using other tobacco products?	SINGLE_CHOICE	social4	G:RA:social4

- Display Conditions: ((If Who are you with? IS Friends) OR (If Who are you with? IS Acquaintances) OR (If Who are you with? IS Family members) OR (If Who are you with? IS Coworkers) OR (If Who are you with? IS Romantic partner) OR (If Who are you with? IS Unknown person))
- Question Groups: None
- Response Required: Yes
- Randomize Choice: No Choices
- Seconds to Answer: 0
- Seconds Until Next Button Becomes Active: 0

Breakdown of Each Trigger

Name	Type	Settings
1)	ONCE	Runs from 2024-04-08T11:37:00 UNTIL 2025-04-08T00:00:00.000Z
2) RA (Control) (9:00am-1:00pm)	schedule	false
3) RA (Control) (1:00pm-5:00pm)	schedule	false
4) RA (Control) (5:00pm-9:00pm)	schedule	false
5) RA (Intervention) (9:00am-1:00pm)	schedule	false
6) RA (Intervention) (1:00pm-5:00pm)	schedule	false
7) RA (Intervention) (5:00pm-9:00pm)	schedule	false

Question & Settings	Question Type	Variable Name	Global Variable Name
15) Who was smoking cigarettes or using other tobacco products?	MULTIPLE_CHOICE	social5	G:RA:social5

- Display Conditions: If Other people smoking cigarettes or using other tobacco products? IS Yes
- Question Groups: None
- Response Required: Yes
- Randomize Choice: No Choices
- Seconds to Answer: 0
- Seconds Until Next Button Becomes Active: 0

Breakdown of Each Trigger

Name	Type	Settings
1)	ONCE	Runs from 2024-04-08T11:37:00 UNTIL 2025-04-08T00:00:00.000Z
2) RA (Control) (9:00am-1:00pm)	schedule	false
3) RA (Control) (1:00pm-5:00pm)	schedule	false
4) RA (Control) (5:00pm-9:00pm)	schedule	false
5) RA (Intervention) (9:00am-1:00pm)	schedule	false
6) RA (Intervention) (1:00pm-5:00pm)	schedule	false
7) RA (Intervention) (5:00pm-9:00pm)	schedule	false

Question & Settings	Question Type	Variable Name	Global Variable Name
16) How many people were smoking cigarettes or using other tobacco products?	SINGLE_CHOICE	social6	G:RA:social6

- Display Conditions: If Other people smoking cigarettes or using other tobacco products? IS Yes
- Question Groups: None
- Response Required: Yes
- Randomize Choice: No Choices
- Seconds to Answer: 0
- Seconds Until Next Button Becomes Active: 0

Breakdown of Each Trigger

Name	Type	Settings
1)	ONCE	Runs from 2024-04-08T11:37:00 UNTIL 2025-04-08T00:00:00.000Z
2) RA (Control) (9:00am-1:00pm)	schedule	false
3) RA (Control) (1:00pm-5:00pm)	schedule	false
4) RA (Control) (5:00pm-9:00pm)	schedule	false
5) RA (Intervention) (9:00am-1:00pm)	schedule	false
6) RA (Intervention) (1:00pm-5:00pm)	schedule	false
7) RA (Intervention) (5:00pm-9:00pm)	schedule	false

Question & Settings	Question Type	Variable Name	Global Variable Name
17) How many people were smoking cigarettes or using other tobacco products?	DROPDOWN	social6_dropdown	G:RA:social6_dropdown

- Display Conditions: If Other people smoking cigarettes or using other tobacco products? IS Yes
- Question Groups: None
- Response Required: Yes
- Randomize Choice: No Choices

- Seconds to Answer: 0
- Seconds Until Next Button Becomes Active: 0

Breakdown of Each Trigger

Name	Type	Settings
1)	ONCE	Runs from 2024-04-08T11:37:00 UNTIL 2025-04-08T00:00:00.000Z
2) RA (Control) (9:00am-1:00pm)	schedule	false
3) RA (Control) (1:00pm-5:00pm)	schedule	false
4) RA (Control) (5:00pm-9:00pm)	schedule	false
5) RA (Intervention) (9:00am-1:00pm)	schedule	false
6) RA (Intervention) (1:00pm-5:00pm)	schedule	false
7) RA (Intervention) (5:00pm-9:00pm)	schedule	false

Question & Settings	Question Type	Variable Name	Global Variable Name
18) What were you doing?	SINGLE_CHOICE	activity	G:RA:activity

- Display Conditions: None
- Question Groups: None
- Response Required: Yes
- Randomize Choice: No Choices
- Seconds to Answer: 0
- Seconds Until Next Button Becomes Active: 0

Breakdown of Each Trigger

Name	Type	Settings

1)	ONCE	Runs from 2024-04-08T11:37:00 UNTIL 2025-04-08T00:00:00.000Z
2) RA (Control) (9:00am-1:00pm)	schedule	false
3) RA (Control) (1:00pm-5:00pm)	schedule	false
4) RA (Control) (5:00pm-9:00pm)	schedule	false
5) RA (Intervention) (9:00am-1:00pm)	schedule	false
6) RA (Intervention) (1:00pm-5:00pm)	schedule	false
7) RA (Intervention) (5:00pm-9:00pm)	schedule	false

Question & Settings	Question Type	Variable Name	Global Variable Name
19) What other activities?	TEXT	activityother	G:RA:activityother

- Display Conditions: If What were you doing? IS Other activities
- Question Groups: None
- Response Required: Yes
- Randomize Choice: No Choices
- Seconds to Answer: 0
- Seconds Until Next Button Becomes Active: 0

Breakdown of Each Trigger

Name	Type	Settings
1)	ONCE	Runs from 2024-04-08T11:37:00 UNTIL 2025-04-08T00:00:00.000Z
2) RA (Control) (9:00am-1:00pm)	schedule	false
3) RA (Control) (1:00pm-5:00pm)	schedule	false
4) RA (Control) (5:00pm-9:00pm)	schedule	false

5) RA (Intervention) (9:00am-1:00pm)	schedule	false
6) RA (Intervention) (1:00pm-5:00pm)	schedule	false
7) RA (Intervention) (5:00pm-9:00pm)	schedule	false

Question & Settings	Question Type	Variable Name	Global Variable Name
20) Consume any of the following in last hour?	MULTIPLE_CHOICE	consume	G:RA:consume

- Display Conditions: None
- Question Groups: None
- Response Required: Yes
- Randomize Choice: No Choices
- Seconds to Answer: 0
- Seconds Until Next Button Becomes Active: 0

Breakdown of Each Trigger

Name	Type	Settings
1)	ONCE	Runs from 2024-04-08T11:37:00 UNTIL 2025-04-08T00:00:00.000Z
2) RA (Control) (9:00am-1:00pm)	schedule	false
3) RA (Control) (1:00pm-5:00pm)	schedule	false
4) RA (Control) (5:00pm-9:00pm)	schedule	false
5) RA (Intervention) (9:00am-1:00pm)	schedule	false
6) RA (Intervention) (1:00pm-5:00pm)	schedule	false
7) RA (Intervention) (5:00pm-9:00pm)	schedule	false

Question & Settings	Question Type	Variable Name	Global Variable Name
21) Intoxicated and/or drunk?	SINGLE_CHOICE	intoxicated	G:RA:intoxicated

- Display Conditions: None
- Question Groups: None
- Response Required: Yes
- Randomize Choice: No Choices
- Seconds to Answer: 0
- Seconds Until Next Button Becomes Active: 0

Breakdown of Each Trigger

Name	Type	Settings
1)	ONCE	Runs from 2024-04-08T11:37:00 UNTIL 2025-04-08T00:00:00.000Z
2) RA (Control) (9:00am-1:00pm)	schedule	false
3) RA (Control) (1:00pm-5:00pm)	schedule	false
4) RA (Control) (5:00pm-9:00pm)	schedule	false
5) RA (Intervention) (9:00am-1:00pm)	schedule	false
6) RA (Intervention) (1:00pm-5:00pm)	schedule	false
7) RA (Intervention) (5:00pm-9:00pm)	schedule	false

Question & Settings	Question Type	Variable Name	Global Variable Name
22) Saw any of the following?	MULTIPLE_CHOICE	exposed	G:RA:exposed

- Display Conditions: None
- Question Groups: None
- Response Required: Yes

- Randomize Choice: No Choices
- Seconds to Answer: 0
- Seconds Until Next Button Becomes Active: 0

Breakdown of Each Trigger

Name	Type	Settings
1)	ONCE	Runs from 2024-04-08T11:37:00 UNTIL 2025-04-08T00:00:00.000Z
2) RA (Control) (9:00am-1:00pm)	schedule	false
3) RA (Control) (1:00pm-5:00pm)	schedule	false
4) RA (Control) (5:00pm-9:00pm)	schedule	false
5) RA (Intervention) (9:00am-1:00pm)	schedule	false
6) RA (Intervention) (1:00pm-5:00pm)	schedule	false
7) RA (Intervention) (5:00pm-9:00pm)	schedule	false

Question & Settings	Question Type	Variable Name	Global Variable Name
23) SINCE THE LAST SURVEY: I felt discriminated against.	SINGLE_CHOICE	discrimination1	G:RA:discrimination1

- Display Conditions: None
- Question Groups: None
- Response Required: Yes
- Randomize Choice: No Choices
- Seconds to Answer: 0
- Seconds Until Next Button Becomes Active: 0

Breakdown of Each Trigger

Name	Type	Settings
1)	ONCE	Runs from 2024-04-08T11:37:00 UNTIL 2025-04-08T00:00:00.000Z
2) RA (Control) (9:00am-1:00pm)	schedule	false
3) RA (Control) (1:00pm-5:00pm)	schedule	false
4) RA (Control) (5:00pm-9:00pm)	schedule	false
5) RA (Intervention) (9:00am-1:00pm)	schedule	false
6) RA (Intervention) (1:00pm-5:00pm)	schedule	false
7) RA (Intervention) (5:00pm-9:00pm)	schedule	false

Question & Settings	Question Type	Variable Name	Global Variable Name
24) SINCE THE LAST SURVEY: What was the main reason(s) for the discrimination that you experienced?	MULTIPLE_CHOICE	discrimination2	G:RA:discrimination2

- Display Conditions: If SINCE THE LAST SURVEY: I felt discriminated against. IS Yes
- Question Groups: None
- Response Required: Yes
- Randomize Choice: No Choices
- Seconds to Answer: 0
- Seconds Until Next Button Becomes Active: 0

Breakdown of Each Trigger

Name	Type	Settings
1)	ONCE	Runs from 2024-04-08T11:37:00 UNTIL

		2025-04-08T00:00:00.000Z
2) RA (Control) (9:00am-1:00pm)	schedule	false
3) RA (Control) (1:00pm-5:00pm)	schedule	false
4) RA (Control) (5:00pm-9:00pm)	schedule	false
5) RA (Intervention) (9:00am-1:00pm)	schedule	false
6) RA (Intervention) (1:00pm-5:00pm)	schedule	false
7) RA (Intervention) (5:00pm-9:00pm)	schedule	false

Question & Settings	Question Type	Variable Name	Global Variable Name
25) SINCE THE LAST SURVEY: Who discriminated against you?	MULTIPLE_CHOICE	discrimination3	G:RA:discrimination3

- Display Conditions: If SINCE THE LAST SURVEY: I felt discriminated against. IS Yes
- Question Groups: None
- Response Required: Yes
- Randomize Choice: No Choices
- Seconds to Answer: 0
- Seconds Until Next Button Becomes Active: 0

Breakdown of Each Trigger

Name	Type	Settings
1)	ONCE	Runs from 2024-04-08T11:37:00 UNTIL 2025-04-08T00:00:00.000Z
2) RA (Control) (9:00am-1:00pm)	schedule	false
3) RA (Control) (1:00pm-5:00pm)	schedule	false
4) RA (Control) (5:00pm-9:00pm)	schedule	false

5) RA (Intervention) (9:00am-1:00pm)	schedule	false
6) RA (Intervention) (1:00pm-5:00pm)	schedule	false
7) RA (Intervention) (5:00pm-9:00pm)	schedule	false

Survey Information for Location Assessment Follow-Up

- Name: Location Assessment Follow-Up
- Type: MOBILE
- Enable Survey End Screen: No
- Randomized Question Order: No
- Disable Review Question Screen: Yes
- Randomly Trigger a Single Group: No
- Number of Survey Triggers: 3
- Number of Questions: 16

Data Format

Variable Name	Format	Position	Description
Response ID	Character	1	Unique Response Identifier
User ID	Character	2	Unique Participant Identifier
Survey Started Date	Character	3	Date participant started to answer the survey
Survey Started Time	Character	4	Time participant started to answer the survey
Survey Submitted Date	Character	5	Date participant submitted survey
Survey Submitted Time	Character	6	Time participant submitted

			survey
Time Zone	Character	7	Identifies the time zone in UTC of the paricipant at the time of survey submission
Submission Location	Numeric	8	Location of the user at the time of response submission
Device OS	Character	9	Identifies the device operating system at the time of submission
Device OS Version	Character	10	Identifies the version device operating system at the time of submission
App Version	Numeric	11	Identifies the app version downloaded on the participant's device at the time of submission
Trigger Date	Character	12	Identifies the date that the trigger was prompted to participant
Trigger Time	Character	13	Identifies the time that the trigger was prompted to participant
Trigger Type	Character	14	Identifies the type of trigger that was prompted to participant
Trigger Name	Character	15	Identifies the name of trigger that was prompted to participant
Trigger Index	Numeric	16	Identifies the order of the trigger based on the parent prompt that the trigger was based on

Question Level Response Variables

Question	Variable Name	Format	Position	Question Type	Choices: Coded Value
Overall feeling right now?	pgf_feeling	Character	17	SINGLE_CHOICE	- 1 - Very unpleasant : 1<br>- 2 : 2<br>- 3 : 3<br>- 4 : 4<br>- 5 - Very pleasant : 5
Overall arousal/energy level right now?	pgf_arousal	Character	18	SINGLE_CHOICE	- 1 - Very low : 1<br>- 2 : 2<br>- 3 : 3<br>- 4 : 4<br>- 5 - Very high : 5
Overall stress level right now?	pgf_stress	Character	19	SINGLE_CHOICE	- 1 - Very low : 1<br>- 2 : 2<br>- 3 : 3<br>- 4 : 4<br>- 5 - Very high : 5
Overall anxiety level right now?	pgf_anxiety	Character	20	SINGLE_CHOICE	- 1 - Very low : 1<br>- 2 : 2<br>- 3 : 3<br>- 4 : 4<br>- 5 - Very high : 5
Craving a cigarette or tobacco product?	pgf_craving	Character	21	SINGLE_CHOICE	- 1 - Very low : 1<br>- 2 : 2<br>- 3 : 3<br>- 4 : 4<br>- 5 - Very high : 5
Did you smoke a cigarette since completing the Location Assessment?	lastsmoke1	Character	22	SINGLE_CHOICE	- Yes : 1<br>- No : 0
Did you use other tobacco or nicotine	lasttobacco1	Character	23	SINGLE_CHOICE	- Yes : 1

products since completing the Location Assessment?					- No : 0
What tobacco product?	lasttobacco2	Character	24	MULTIPLE_CHOICE	- Cigarillo : 1<br>- Dip, Snus, or other smokeless : 2<br>- Hookah : 3<br>- E-cigarette, nicotine vape : 4<br>- Other tobacco product : 5
What kind of tobacco product?	lasttobaccoother	Character	25	TEXT	
Did you see an intervention message and picture when you completed the most recent location assessment?	message_displayed	Character	26	SINGLE_CHOICE	- Yes, I saw a message and a picture : 1<br>- No, there was no message or picture : 0
Do you remember the intervention message?	remembermessage	Character	27	SINGLE_CHOICE	- Yes : 1<br>- No : 0
How would you rate the content (that is, the words and meaning) of the last intervention message?	contentrating	Character	28	SINGLE_CHOICE	- Very poor : 1<br>- Poor : 2<br>- Acceptable : 3<br>- Good : 4<br>- Very good : 5
How would you rate the design (that is, how the message looks) of the last intervention message?	designrating	Character	29	SINGLE_CHOICE	- Very poor : 1<br>- Poor : 2<br>- Acceptable : 3<br>- Good : 4<br>- Very good : 5
How helpful was the last intervention message for coping with a smoking urge?	urgehelpful	Character	30	SINGLE_CHOICE	- Not at all helpful : 1<br>- Somewhat helpful : 2<br>- Moderately helpful : 3<br>- Very helpful : 4

					- Extremely helpful : 5
How helpful was the last intervention message for supporting you in quitting or reducing smoking?	supporthelpful	Character	31	SINGLE_CHOICE	- Not at all helpful : 1<br>- Somewhat helpful : 2<br>- Moderately helpful : 3<br>- Very helpful : 4<br>- Extremely helpful : 5
Did you follow the suggestion of the last intervention message?	followsuggestion	Character	32	SINGLE_CHOICE	- Yes : 1<br>- No : 0

Breakdown of Each Question

Question & Settings	Question Type	Variable Name	Global Variable Name
1) Overall feeling right now?	SINGLE_CHOICE	pgf_feeling	G:LAF:pgf_feeling

- Display Conditions: None
- Question Groups: None
- Response Required: Yes
- Randomize Choice: No Choices
- Seconds to Answer: 0
- Seconds Until Next Button Becomes Active: 0

Breakdown of Each Trigger

Name	Type	Settings
1) Location assessment follow-up	participantAction	false
2) Location assessment follow-up (v2)	participantAction	false
3) Location assessment follow-up (v3)	participantAction	false

Question & Settings	Question Type	Variable Name	Global Variable Name
2) Overall arousal/energy level right now?	SINGLE_CHOICE	pgf_arousal	G:LAF:pgf_arousal

- Display Conditions: None
- Question Groups: None
- Response Required: Yes
- Randomize Choice: No Choices
- Seconds to Answer: 0
- Seconds Until Next Button Becomes Active: 0

Breakdown of Each Trigger

Name	Type	Settings
1) Location assessment follow-up	participantAction	false
2) Location assessment follow-up (v2)	participantAction	false
3) Location assessment follow-up (v3)	participantAction	false

Question & Settings	Question Type	Variable Name	Global Variable Name
3) Overall stress level right now?	SINGLE_CHOICE	pgf_stress	G:LAF:pgf_stress

- Display Conditions: None
- Question Groups: None
- Response Required: Yes
- Randomize Choice: No Choices
- Seconds to Answer: 0
- Seconds Until Next Button Becomes Active: 0

Breakdown of Each Trigger

Name	Type	Settings
1) Location assessment follow-up	participantAction	false
2) Location assessment follow-up (v2)	participantAction	false
3) Location assessment follow-up (v3)	participantAction	false

Question & Settings	Question Type	Variable Name	Global Variable Name
4) Overall anxiety level right now?	SINGLE_CHOICE	pgf_anxiety	G:LAF:pgf_anxiety

- Display Conditions: None
- Question Groups: None
- Response Required: Yes
- Randomize Choice: No Choices
- Seconds to Answer: 0
- Seconds Until Next Button Becomes Active: 0

Breakdown of Each Trigger

Name	Type	Settings
1) Location assessment follow-up	participantAction	false
2) Location assessment follow-up (v2)	participantAction	false
3) Location assessment follow-up (v3)	participantAction	false

Question & Settings	Question Type	Variable Name	Global Variable Name
5) Craving a cigarette or tobacco product?	SINGLE_CHOICE	pgf_craving	G:LAF:pgf_craving

- Display Conditions: None

- Question Groups: None
- Response Required: Yes
- Randomize Choice: No Choices
- Seconds to Answer: 0
- Seconds Until Next Button Becomes Active: 0

Breakdown of Each Trigger

Name	Type	Settings
1) Location assessment follow-up	participantAction	false
2) Location assessment follow-up (v2)	participantAction	false
3) Location assessment follow-up (v3)	participantAction	false

Question & Settings	Question Type	Variable Name	Global Variable Name
6) Did you smoke a cigarette since completing the Location Assessment?	SINGLE_CHOICE	lastsmoke1	G:LAF:lastsmoke1

- Display Conditions: None
- Question Groups: None
- Response Required: Yes
- Randomize Choice: No Choices
- Seconds to Answer: 0
- Seconds Until Next Button Becomes Active: 0

Breakdown of Each Trigger

Name	Type	Settings
1) Location assessment follow-up	participantAction	false

2) Location assessment follow-up (v2)	participantAction	false
3) Location assessment follow-up (v3)	participantAction	false

Question & Settings	Question Type	Variable Name	Global Variable Name
7) Did you use other tobacco or nicotine products since completing the Location Assessment?	SINGLE_CHOICE	lasttobacco1	G:LAF:lasttobacco1

- Display Conditions: None
- Question Groups: None
- Response Required: Yes
- Randomize Choice: No Choices
- Seconds to Answer: 0
- Seconds Until Next Button Becomes Active: 0

Breakdown of Each Trigger

Name	Type	Settings
1) Location assessment follow-up	participantAction	false
2) Location assessment follow-up (v2)	participantAction	false
3) Location assessment follow-up (v3)	participantAction	false

Question & Settings	Question Type	Variable Name	Global Variable Name
8) What tobacco product?	MULTIPLE_CHOICE	lasttobacco2	G:LAF:lasttobacco2

- Display Conditions: If Did you use other tobacco or nicotine products since completing the Location Assessment? IS Yes
- Question Groups: None
- Response Required: Yes

- Randomize Choice: No Choices
- Seconds to Answer: 0
- Seconds Until Next Button Becomes Active: 0

Breakdown of Each Trigger

Name	Type	Settings
1) Location assessment follow-up	participantAction	false
2) Location assessment follow-up (v2)	participantAction	false
3) Location assessment follow-up (v3)	participantAction	false

Question & Settings	Question Type	Variable Name	Global Variable Name
9) What kind of tobacco product?	TEXT	lasttobaccoother	G:LAF:lasttobaccoother

- Display Conditions: If What tobacco product? IS Other tobacco product
- Question Groups: None
- Response Required: Yes
- Randomize Choice: No Choices
- Seconds to Answer: 0
- Seconds Until Next Button Becomes Active: 0

Breakdown of Each Trigger

Name	Type	Settings
1) Location assessment follow-up	participantAction	false
2) Location assessment follow-up (v2)	participantAction	false
3) Location assessment follow-up (v3)	participantAction	false

Question & Settings	Question Type	Variable Name	Global Variable Name
10) Did you see an intervention message and picture when you completed the most recent location assessment?	SINGLE_CHOICE	message_displayed	G:LAF:message_displayed

- Display Conditions: None
- Question Groups: None
- Response Required: Yes
- Randomize Choice: No Choices
- Seconds to Answer: 0
- Seconds Until Next Button Becomes Active: 0

Breakdown of Each Trigger

Name	Type	Settings
1) Location assessment follow-up	participantAction	false
2) Location assessment follow-up (v2)	participantAction	false
3) Location assessment follow-up (v3)	participantAction	false

Question & Settings	Question Type	Variable Name	Global Variable Name
11) Do you remember the intervention message?	SINGLE_CHOICE	remembermessage	G:LAF:remembermessage

- Display Conditions: If Did you see an intervention message and picture when you completed the most recent location assessment? IS Yes, I saw a message and a picture
- Question Groups: None
- Response Required: Yes
- Randomize Choice: No Choices
- Seconds to Answer: 0

- Seconds Until Next Button Becomes Active: 0

Breakdown of Each Trigger

Name	Type	Settings
1) Location assessment follow-up	participantAction	false
2) Location assessment follow-up (v2)	participantAction	false
3) Location assessment follow-up (v3)	participantAction	false

Question & Settings	Question Type	Variable Name	Global Variable Name
12) How would you rate the content (that is, the words and meaning) of the last intervention message?	SINGLE_CHOICE	contentrating	G:LAF:contentrating

- Display Conditions: If Do you remember the intervention message? IS Yes
- Question Groups: None
- Response Required: Yes
- Randomize Choice: No Choices
- Seconds to Answer: 0
- Seconds Until Next Button Becomes Active: 0

Breakdown of Each Trigger

Name	Type	Settings
1) Location assessment follow-up	participantAction	false
2) Location assessment follow-up (v2)	participantAction	false
3) Location assessment follow-up (v3)	participantAction	false

Question & Settings	Question Type	Variable Name	Global Variable Name
13) How would you rate the design (that is, how the message looks) of the last intervention message?	SINGLE_CHOICE	designrating	G:LAF:designrating

- Display Conditions: If Do you remember the intervention message? IS Yes
- Question Groups: None
- Response Required: Yes
- Randomize Choice: No Choices
- Seconds to Answer: 0
- Seconds Until Next Button Becomes Active: 0

Breakdown of Each Trigger

Name	Type	Settings
1) Location assessment follow-up	participantAction	false
2) Location assessment follow-up (v2)	participantAction	false
3) Location assessment follow-up (v3)	participantAction	false

Question & Settings	Question Type	Variable Name	Global Variable Name
14) How helpful was the last intervention message for coping with a smoking urge?	SINGLE_CHOICE	urgehelpful	G:LAF:urgehelpful

- Display Conditions: If Do you remember the intervention message? IS Yes
- Question Groups: None
- Response Required: Yes
- Randomize Choice: No Choices

- Seconds to Answer: 0
- Seconds Until Next Button Becomes Active: 0

Breakdown of Each Trigger

Name	Type	Settings
1) Location assessment follow-up	participantAction	false
2) Location assessment follow-up (v2)	participantAction	false
3) Location assessment follow-up (v3)	participantAction	false

Question & Settings	Question Type	Variable Name	Global Variable Name
15) How helpful was the last intervention message for supporting you in quitting or reducing smoking?	SINGLE_CHOICE	supporthelpful	G:LAF:supporthelpful

- Display Conditions: If Do you remember the intervention message? IS Yes
- Question Groups: None
- Response Required: Yes
- Randomize Choice: No Choices
- Seconds to Answer: 0
- Seconds Until Next Button Becomes Active: 0

Breakdown of Each Trigger

Name	Type	Settings
1) Location assessment follow-up	participantAction	false
2) Location assessment follow-up (v2)	participantAction	false

3) Location assessment follow-up (v3)	participantAction	false

Question & Settings	Question Type	Variable Name	Global Variable Name
16) Did you follow the suggestion of the last intervention message?	SINGLE_CHOICE	followsuggestion	G:LAF:followsuggestion

- Display Conditions: If Do you remember the intervention message? IS Yes
- Question Groups: None
- Response Required: Yes
- Randomize Choice: No Choices
- Seconds to Answer: 0
- Seconds Until Next Button Becomes Active: 0

Breakdown of Each Trigger

Name	Type	Settings
1) Location assessment follow-up	participantAction	false
2) Location assessment follow-up (v2)	participantAction	false
3) Location assessment follow-up (v3)	participantAction	false

Survey Information for Cigarette Follow-Up

- Name: Cigarette Follow-Up
- Type: MOBILE
- Enable Survey End Screen: No
- Randomized Question Order: No
- Disable Review Question Screen: Yes
- Randomly Trigger a Single Group: No

- Number of Survey Triggers: 21
- Number of Questions: 25

Data Format

Variable Name	Format	Position	Description
Response ID	Character	1	Unique Response Identifier
User ID	Character	2	Unique Participant Identifier
Survey Started Date	Character	3	Date participant started to answer the survey
Survey Started Time	Character	4	Time participant started to answer the survey
Survey Submitted Date	Character	5	Date participant submitted survey
Survey Submitted Time	Character	6	Time participant submitted survey
Time Zone	Character	7	Identifies the time zone in UTC of the paricipant at the time of survey submission
Submission Location	Numeric	8	Location of the user at the time of response submission
Device OS	Character	9	Identifies the device operating system at the time of submission
Device OS Version	Character	10	Identifies the version device operating system at the time of submission
App Version	Numeric	11	Identifies the app version downloaded on the participant's

			device at the time of submission
Trigger Date	Character	12	Identifies the date that the trigger was prompted to participant
Trigger Time	Character	13	Identifies the time that the trigger was prompted to participant
Trigger Type	Character	14	Identifies the type of trigger that was prompted to participant
Trigger Name	Character	15	Identifies the name of trigger that was prompted to participant
Trigger Index	Numeric	16	Identifies the order of the trigger based on the parent prompt that the trigger was based on

Question Level Response Variables

Question	Variable Name	Format	Position	Question Type	Choices: Coded Value
JUST BEFORE SMOKING: Overall feeling?	psfeeling	Character	17	SINGLE_CHOICE	- 1 - Very unpleasant : 1<br>- 2 : 2<br>- 3 : 3<br>- 4 : 4<br>- 5 - Very pleasant : 5
JUST BEFORE SMOKING: Overall arousal/energy level?	psarousal	Character	18	SINGLE_CHOICE	- 1 - Very low : 1<br>- 2 : 2<br>- 3 : 3<br>- 4 : 4<br>- 5 - Very high : 5
JUST BEFORE SMOKING: Overall stress level?	psstress	Character	19	SINGLE_CHOICE	- 1 - Very low : 1<br>- 2 : 2<br>- 3 : 3

					- 4 : 4<br>- 5 - Very high : 5
JUST BEFORE SMOKING: Overall anxiety level?	psanxiety	Character	20	SINGLE_CHOICE	- 1 - Very low : 1<br>- 2 : 2<br>- 3 : 3<br>- 4 : 4<br>- 5 - Very high : 5
JUST BEFORE SMOKING: Craving a cigarette or tobacco product?	pscraving	Character	21	SINGLE_CHOICE	- 1 - Very low : 1<br>- 2 : 2<br>- 3 : 3<br>- 4 : 4<br>- 5 - Very high : 5
Where were you when you decided to smoke?	pslocation	Character	22	SINGLE_CHOICE	- Home : 1<br>- Workplace/School : 2<br>- Other's home : 3<br>- Bar : 4<br>- Restaurant : 5<br>- Vehicle : 6<br>- Walking between places : 7<br>- Public transit stop : 8<br>- Other location : 9
Location when you decided to smoke?	pslocationother	Character	23	TEXT	
JUST BEFORE SMOKING: Location? Inside/outside.	psinsideoutside	Character	24	SINGLE_CHOICE	- Inside : 1<br>- Outside (patio, entrance, street, etc.) : 2
JUST BEFORE SMOKING: Smoking cigarettes allowed?	pscigsallowed	Character	25	SINGLE_CHOICE	- Forbidden : 1<br>- Discouraged : 2<br>- Allowed : 3

Did you change location to smoke?	pschangelocation	Character	26	SINGLE_CHOICE	- Yes : 1<br>- No : 0
JUST BEFORE SMOKING: With others?	pssocial2	Character	27	MULTIPLE_CHOIC<br>E	- Friends : 2<br>- Acquaintances : 3<br>- Family members : 4<br>- Coworkers : 5<br>- Romantic partner : 6<br>- Unknown person : 7
JUST BEFORE SMOKING: With how many people?	pssocial3	Character	28	SINGLE_CHOICE	- 1 : 1<br>- 2-4 : 2<br>- 5-20 : 3<br>- 21+ : 4
JUST BEFORE SMOKING: With how many people?	pssocial3_dropdown	Character	29	DROPDOWN	-<br>1 : 1<br>- 2 : 2<br>- 3 : 3<br>- 4 : 4<br>- 5 : 5<br>- 6 : 6<br>- 7 : 7<br>- 8 : 8<br>- 9 : 9<br>- 10 : 10<br>- 11 : 11<br>- 12 : 12<br>- 13 : 13<br>- 14 : 14<br>- 15 : 15<br>- 16 : 16<br>- 17 : 17<br>- 18 : 18<br>- 19 : 19

					- 20 : 20<br>- 21+ : 21
JUST BEFORE SMOKING: Other people smoking cigarettes or using tobacco products?	pssocial4	Character	30	SINGLE_CHOICE	- Yes : 1<br>- No : 0
JUST BEFORE SMOKING: Who was smoking cigarettes or using tobacco products?	pssocial5	Character	31	MULTIPLE_CHOICE	- Friends : 1<br>- Acquaintances : 2<br>- Family members : 3<br>- Coworkers : 4<br>- Romantic partner : 5<br>- Unknown person : 6
JUST BEFORE SMOKING: How many people were smoking or using tobacco products?	pssocial6	Character	32	SINGLE_CHOICE	- 1 : 1<br>- 2-4 : 2<br>- 5-20 : 3<br>- 21+ : 4
JUST BEFORE SMOKING: How many people were smoking or using tobacco products?	pssocial6_dropdown	Character	33	DROPDOWN	-<br>1 : 1<br>- 2 : 2<br>- 3 : 3<br>- 4 : 4<br>- 5 : 5<br>- 6 : 6<br>- 7 : 7<br>- 8 : 8<br>- 9 : 9<br>- 10 : 10<br>- 11 : 11<br>- 12 : 12<br>- 13 : 13<br>- 14 : 14<br>- 15 : 15

					- 16 : 16<br>- 17 : 17<br>- 18 : 18<br>- 19 : 19<br>- 20 : 20<br>- 21+ : 21
JUST BEFORE SMOKING: What were you doing?	psactivity	Character	34	SINGLE_CHOICE	- Working/Chores : 1<br>- Inactive/leisure : 2<br>- Interacting with others : 3<br>- Eating/drinking : 4<br>- Between activities : 5<br>- Other activities : 6
JUST BEFORE SMOKING: What other activities?	psactivityother	Character	35	TEXT	
Consume any of the following in last hour?	psconsume	Character	36	MULTIPLE_CHOICE	- Food : 1<br>- Caffeinated drink : 2<br>- Non-caffeinated drink : 3<br>- Alcohol : 4<br>- Cigarettes : 5<br>- Other tobacco product : 6<br>- Marijuana or Cannabis : 7<br>- Other substance or drug : 8
JUST BEFORE SMOKING: Intoxicated/drunk?	psintoxicated	Character	37	SINGLE_CHOICE	- 1 - No!! : 1<br>- 2 : 2<br>- 3 : 3<br>- 4 : 4<br>- 5 - Yes!! : 5
JUST BEFORE SMOKING: Saw any of the following?	psexposed	Character	38	MULTIPLE_CHOICE	- Cigarette or tobacco product : 1<br>- Lighter/matches : 2

					- Cigarette or tobacco product pack : 3<br>- Ashtray : 4<br>- Cigarette or tobacco product in the media : 5<br>- Someone smoking or using tobacco product : 6<br>- Other things related to smoking or tobacco products : 7
SINCE THE LAST SURVEY: I felt discriminated against.	psdiscrimination1	Character	39	SINGLE_CHOICE	- Yes : 1<br>- No : 0
SINCE THE LAST SURVEY: What was the main reason (s) for the discrimination that you experienced?	psdiscrimination2	Character	40	MULTIPLE_CHOICE	- Your age : 1<br>- Your gender : 2<br>- Your race : 3<br>- Your ethnicity or nationality : 4<br>- Your religion : 5<br>- Your height or weight : 6<br>- Some other aspect of your appearance : 7<br>- A physical disability : 8<br>- Your sexual orientation : 9<br>- Being a smoker : 10<br>- Being poor : 11<br>- Other : 12
SINCE THE LAST SURVEY: Who discriminated against you?	psdiscrimination3	Character	41	MULTIPLE_CHOICE	- Family member : 1<br>- Romantic partner : 2<br>- Stranger : 4<br>- Acquaintance : 5<br>- Friend : 6<br>- Employer : 7

- Other : 8

Breakdown of Each Question

Question & Settings	Question Type	Variable Name	Global Variable Name
1) JUST BEFORE SMOKING: Overall feeling?	SINGLE_CHOICE	psfeeling	G:CF:psfeeling

- Display Conditions: None
- Question Groups: None
- Response Required: Yes
- Randomize Choice: No Choices
- Seconds to Answer: 0
- Seconds Until Next Button Becomes Active: 0

Breakdown of Each Trigger

Name	Type	Settings
1) 1 CPD > Probability = 100%	participantAction	false
2) 2 CPD > Probability = 100%	participantAction	false
3) 4 CPD > Probability = 75%	participantAction	false
4) 5 CPD > Probability = 60%	participantAction	false
5) 6 CPD > Probability = 50%	participantAction	false
6) 7 CPD > Probability = 43%	participantAction	false
7) 8 CPD > Probability = 38%	participantAction	false
8) 9 CPD > Probability = 33%	participantAction	false
9) 11 CPD > Probability = 27%	participantAction	false

10) 12 CPD > Probability = 25%	participantAction	false
11) 14 CPD > Probability = 21%	participantAction	false
12) 15 CPD > Probability = 20%	participantAction	false
13) 16 CPD > Probability = 19%	participantAction	false
14) 17 CPD > Probability = 18%	participantAction	false
15) 18 CPD > Probability = 17%	participantAction	false
16) 19 CPD > Probability = 16%	participantAction	false
17) >= 20 CPD > Probability = 15%	participantAction	false
18) 13 CPD > Probability = 23%	participantAction	false
19) 3 CPD > Probability = 100%	participantAction	false
20) 10 CPD > Probability = 30%	participantAction	false
21) PARTICIPANTACTION Trigger - test	participantAction	false

Question & Settings	Question Type	Variable Name	Global Variable Name
2) JUST BEFORE SMOKING: Overall arousal/energy level?	SINGLE_CHOICE	psarousal	G:CF:psarousal

- Display Conditions: None
- Question Groups: None
- Response Required: Yes
- Randomize Choice: No Choices
- Seconds to Answer: 0
- Seconds Until Next Button Becomes Active: 0

Breakdown of Each Trigger

Name	Type	Settings

1) 1 CPD > Probability = 100%	participantAction	false
2) 2 CPD > Probability = 100%	participantAction	false
3) 4 CPD > Probability = 75%	participantAction	false
4) 5 CPD > Probability = 60%	participantAction	false
5) 6 CPD > Probability = 50%	participantAction	false
6) 7 CPD > Probability = 43%	participantAction	false
7) 8 CPD > Probability = 38%	participantAction	false
8) 9 CPD > Probability = 33%	participantAction	false
9) 11 CPD > Probability = 27%	participantAction	false
10) 12 CPD > Probability = 25%	participantAction	false
11) 14 CPD > Probability = 21%	participantAction	false
12) 15 CPD > Probability = 20%	participantAction	false
13) 16 CPD > Probability = 19%	participantAction	false
14) 17 CPD > Probability = 18%	participantAction	false
15) 18 CPD > Probability = 17%	participantAction	false
16) 19 CPD > Probability = 16%	participantAction	false
17) >= 20 CPD > Probability = 15%	participantAction	false
18) 13 CPD > Probability = 23%	participantAction	false
19) 3 CPD > Probability = 100%	participantAction	false
20) 10 CPD > Probability = 30%	participantAction	false
21) PARTICIPANTACTION Trigger - test	participantAction	false

Question & Settings	Question Type	Variable Name	Global Variable Name
3) JUST BEFORE SMOKING: Overall stress level?	SINGLE_CHOICE	psstress	G:CF:psstress

- Display Conditions: None
- Question Groups: None
- Response Required: Yes
- Randomize Choice: No Choices
- Seconds to Answer: 0
- Seconds Until Next Button Becomes Active: 0

Breakdown of Each Trigger

Name	Type	Settings
1) 1 CPD > Probability = 100%	participantAction	false
2) 2 CPD > Probability = 100%	participantAction	false
3) 4 CPD > Probability = 75%	participantAction	false
4) 5 CPD > Probability = 60%	participantAction	false
5) 6 CPD > Probability = 50%	participantAction	false
6) 7 CPD > Probability = 43%	participantAction	false
7) 8 CPD > Probability = 38%	participantAction	false
8) 9 CPD > Probability = 33%	participantAction	false
9) 11 CPD > Probability = 27%	participantAction	false
10) 12 CPD > Probability = 25%	participantAction	false
11) 14 CPD > Probability = 21%	participantAction	false
12) 15 CPD > Probability = 20%	participantAction	false
13) 16 CPD > Probability = 19%	participantAction	false
14) 17 CPD > Probability = 18%	participantAction	false
15) 18 CPD > Probability = 17%	participantAction	false

16) 19 CPD > Probability = 16%	participantAction	false
17) >= 20 CPD > Probability = 15%	participantAction	false
18) 13 CPD > Probability = 23%	participantAction	false
19) 3 CPD > Probability = 100%	participantAction	false
20) 10 CPD > Probability = 30%	participantAction	false
21) PARTICIPANTACTION Trigger - test	participantAction	false

Question & Settings	Question Type	Variable Name	Global Variable Name
4) JUST BEFORE SMOKING: Overall anxiety level?	SINGLE_CHOICE	psanxiety	G:CF:psanxiety

- Display Conditions: None
- Question Groups: None
- Response Required: Yes
- Randomize Choice: No Choices
- Seconds to Answer: 0
- Seconds Until Next Button Becomes Active: 0

Breakdown of Each Trigger

Name	Type	Settings
1) 1 CPD > Probability = 100%	participantAction	false
2) 2 CPD > Probability = 100%	participantAction	false
3) 4 CPD > Probability = 75%	participantAction	false
4) 5 CPD > Probability = 60%	participantAction	false
5) 6 CPD > Probability = 50%	participantAction	false
6) 7 CPD > Probability = 43%	participantAction	false

7) 8 CPD > Probability = 38%	participantAction	false
8) 9 CPD > Probability = 33%	participantAction	false
9) 11 CPD > Probability = 27%	participantAction	false
10) 12 CPD > Probability = 25%	participantAction	false
11) 14 CPD > Probability = 21%	participantAction	false
12) 15 CPD > Probability = 20%	participantAction	false
13) 16 CPD > Probability = 19%	participantAction	false
14) 17 CPD > Probability = 18%	participantAction	false
15) 18 CPD > Probability = 17%	participantAction	false
16) 19 CPD > Probability = 16%	participantAction	false
17) >= 20 CPD > Probability = 15%	participantAction	false
18) 13 CPD > Probability = 23%	participantAction	false
19) 3 CPD > Probability = 100%	participantAction	false
20) 10 CPD > Probability = 30%	participantAction	false
21) PARTICIPANTACTION Trigger - test	participantAction	false

Question & Settings	Question Type	Variable Name	Global Variable Name
5) JUST BEFORE SMOKING: Craving a cigarette or tobacco product?	SINGLE_CHOICE	pscraving	G:CF:pscraving

- Display Conditions: None
- Question Groups: None
- Response Required: Yes
- Randomize Choice: No Choices
- Seconds to Answer: 0
- Seconds Until Next Button Becomes Active: 0

Breakdown of Each Trigger

Name	Type	Settings
1) 1 CPD > Probability = 100%	participantAction	false
2) 2 CPD > Probability = 100%	participantAction	false
3) 4 CPD > Probability = 75%	participantAction	false
4) 5 CPD > Probability = 60%	participantAction	false
5) 6 CPD > Probability = 50%	participantAction	false
6) 7 CPD > Probability = 43%	participantAction	false
7) 8 CPD > Probability = 38%	participantAction	false
8) 9 CPD > Probability = 33%	participantAction	false
9) 11 CPD > Probability = 27%	participantAction	false
10) 12 CPD > Probability = 25%	participantAction	false
11) 14 CPD > Probability = 21%	participantAction	false
12) 15 CPD > Probability = 20%	participantAction	false
13) 16 CPD > Probability = 19%	participantAction	false
14) 17 CPD > Probability = 18%	participantAction	false
15) 18 CPD > Probability = 17%	participantAction	false
16) 19 CPD > Probability = 16%	participantAction	false
17) >= 20 CPD > Probability = 15%	participantAction	false
18) 13 CPD > Probability = 23%	participantAction	false
19) 3 CPD > Probability = 100%	participantAction	false
20) 10 CPD > Probability = 30%	participantAction	false
21) PARTICIPANTACTION Trigger - test	participantAction	false

Question & Settings	Question Type	Variable Name	Global Variable Name
6) Where were you when you decided to smoke?	SINGLE_CHOICE	pslocation	G:CF:pslocation

- Display Conditions: None
- Question Groups: None
- Response Required: Yes
- Randomize Choice: No Choices
- Seconds to Answer: 0
- Seconds Until Next Button Becomes Active: 0

Breakdown of Each Trigger

Name	Type	Settings
1) 1 CPD > Probability = 100%	participantAction	false
2) 2 CPD > Probability = 100%	participantAction	false
3) 4 CPD > Probability = 75%	participantAction	false
4) 5 CPD > Probability = 60%	participantAction	false
5) 6 CPD > Probability = 50%	participantAction	false
6) 7 CPD > Probability = 43%	participantAction	false
7) 8 CPD > Probability = 38%	participantAction	false
8) 9 CPD > Probability = 33%	participantAction	false
9) 11 CPD > Probability = 27%	participantAction	false
10) 12 CPD > Probability = 25%	participantAction	false
11) 14 CPD > Probability = 21%	participantAction	false
12) 15 CPD > Probability = 20%	participantAction	false

13) 16 CPD > Probability = 19%	participantAction	false
14) 17 CPD > Probability = 18%	participantAction	false
15) 18 CPD > Probability = 17%	participantAction	false
16) 19 CPD > Probability = 16%	participantAction	false
17) >= 20 CPD > Probability = 15%	participantAction	false
18) 13 CPD > Probability = 23%	participantAction	false
19) 3 CPD > Probability = 100%	participantAction	false
20) 10 CPD > Probability = 30%	participantAction	false
21) PARTICIPANTACTION Trigger - test	participantAction	false

Question & Settings	Question Type	Variable Name	Global Variable Name
7) Location when you decided to smoke?	TEXT	pslocationother	G:CF:pslocationother

- Display Conditions: (If Where were you when you decided to smoke? IS Other location)
- Question Groups: None
- Response Required: Yes
- Randomize Choice: No Choices
- Seconds to Answer: 0
- Seconds Until Next Button Becomes Active: 0

Breakdown of Each Trigger

Name	Type	Settings
1) 1 CPD > Probability = 100%	participantAction	false
2) 2 CPD > Probability = 100%	participantAction	false
3) 4 CPD > Probability = 75%	participantAction	false

4) 5 CPD > Probability = 60%	participantAction	false
5) 6 CPD > Probability = 50%	participantAction	false
6) 7 CPD > Probability = 43%	participantAction	false
7) 8 CPD > Probability = 38%	participantAction	false
8) 9 CPD > Probability = 33%	participantAction	false
9) 11 CPD > Probability = 27%	participantAction	false
10) 12 CPD > Probability = 25%	participantAction	false
11) 14 CPD > Probability = 21%	participantAction	false
12) 15 CPD > Probability = 20%	participantAction	false
13) 16 CPD > Probability = 19%	participantAction	false
14) 17 CPD > Probability = 18%	participantAction	false
15) 18 CPD > Probability = 17%	participantAction	false
16) 19 CPD > Probability = 16%	participantAction	false
17) >= 20 CPD > Probability = 15%	participantAction	false
18) 13 CPD > Probability = 23%	participantAction	false
19) 3 CPD > Probability = 100%	participantAction	false
20) 10 CPD > Probability = 30%	participantAction	false
21) PARTICIPANTACTION Trigger - test	participantAction	false

Question & Settings	Question Type	Variable Name	Global Variable Name
8) JUST BEFORE SMOKING: Location? Inside/outside.	SINGLE_CHOICE	psinsideoutside	G:CF:psinsideoutside

- Display Conditions: None
- Question Groups: None
- Response Required: Yes

- Randomize Choice: No Choices
- Seconds to Answer: 0
- Seconds Until Next Button Becomes Active: 0

Breakdown of Each Trigger

Name	Type	Settings
1) 1 CPD > Probability = 100%	participantAction	false
2) 2 CPD > Probability = 100%	participantAction	false
3) 4 CPD > Probability = 75%	participantAction	false
4) 5 CPD > Probability = 60%	participantAction	false
5) 6 CPD > Probability = 50%	participantAction	false
6) 7 CPD > Probability = 43%	participantAction	false
7) 8 CPD > Probability = 38%	participantAction	false
8) 9 CPD > Probability = 33%	participantAction	false
9) 11 CPD > Probability = 27%	participantAction	false
10) 12 CPD > Probability = 25%	participantAction	false
11) 14 CPD > Probability = 21%	participantAction	false
12) 15 CPD > Probability = 20%	participantAction	false
13) 16 CPD > Probability = 19%	participantAction	false
14) 17 CPD > Probability = 18%	participantAction	false
15) 18 CPD > Probability = 17%	participantAction	false
16) 19 CPD > Probability = 16%	participantAction	false
17) >= 20 CPD > Probability = 15%	participantAction	false
18) 13 CPD > Probability = 23%	participantAction	false

19) 3 CPD > Probability = 100%	participantAction	false
20) 10 CPD > Probability = 30%	participantAction	false
21) PARTICIPANTACTION Trigger - test	participantAction	false

Question & Settings	Question Type	Variable Name	Global Variable Name
9) JUST BEFORE SMOKING: Smoking cigarettes allowed?	SINGLE_CHOICE	pscigsallowed	G:CF:pscigsallowed

- Display Conditions: None
- Question Groups: None
- Response Required: Yes
- Randomize Choice: No Choices
- Seconds to Answer: 0
- Seconds Until Next Button Becomes Active: 0

Breakdown of Each Trigger

Name	Type	Settings
1) 1 CPD > Probability = 100%	participantAction	false
2) 2 CPD > Probability = 100%	participantAction	false
3) 4 CPD > Probability = 75%	participantAction	false
4) 5 CPD > Probability = 60%	participantAction	false
5) 6 CPD > Probability = 50%	participantAction	false
6) 7 CPD > Probability = 43%	participantAction	false
7) 8 CPD > Probability = 38%	participantAction	false
8) 9 CPD > Probability = 33%	participantAction	false
9) 11 CPD > Probability = 27%	participantAction	false

10) 12 CPD > Probability = 25%	participantAction	false
11) 14 CPD > Probability = 21%	participantAction	false
12) 15 CPD > Probability = 20%	participantAction	false
13) 16 CPD > Probability = 19%	participantAction	false
14) 17 CPD > Probability = 18%	participantAction	false
15) 18 CPD > Probability = 17%	participantAction	false
16) 19 CPD > Probability = 16%	participantAction	false
17) >= 20 CPD > Probability = 15%	participantAction	false
18) 13 CPD > Probability = 23%	participantAction	false
19) 3 CPD > Probability = 100%	participantAction	false
20) 10 CPD > Probability = 30%	participantAction	false
21) PARTICIPANTACTION Trigger - test	participantAction	false

Question & Settings	Question Type	Variable Name	Global Variable Name
10) Did you change location to smoke?	SINGLE_CHOICE	pschangelocation	G:CF:pschangelocation

- Display Conditions: None
- Question Groups: None
- Response Required: Yes
- Randomize Choice: No Choices
- Seconds to Answer: 0
- Seconds Until Next Button Becomes Active: 0

Breakdown of Each Trigger

Name	Type	Settings

1) 1 CPD > Probability = 100%	participantAction	false
2) 2 CPD > Probability = 100%	participantAction	false
3) 4 CPD > Probability = 75%	participantAction	false
4) 5 CPD > Probability = 60%	participantAction	false
5) 6 CPD > Probability = 50%	participantAction	false
6) 7 CPD > Probability = 43%	participantAction	false
7) 8 CPD > Probability = 38%	participantAction	false
8) 9 CPD > Probability = 33%	participantAction	false
9) 11 CPD > Probability = 27%	participantAction	false
10) 12 CPD > Probability = 25%	participantAction	false
11) 14 CPD > Probability = 21%	participantAction	false
12) 15 CPD > Probability = 20%	participantAction	false
13) 16 CPD > Probability = 19%	participantAction	false
14) 17 CPD > Probability = 18%	participantAction	false
15) 18 CPD > Probability = 17%	participantAction	false
16) 19 CPD > Probability = 16%	participantAction	false
17) >= 20 CPD > Probability = 15%	participantAction	false
18) 13 CPD > Probability = 23%	participantAction	false
19) 3 CPD > Probability = 100%	participantAction	false
20) 10 CPD > Probability = 30%	participantAction	false
21) PARTICIPANTACTION Trigger - test	participantAction	false

Question & Settings	Question Type	Variable Name	Global Variable Name
11) JUST BEFORE SMOKING: With others?	MULTIPLE_CHOICE	pssocial2	G:CF:pssocial2

- Display Conditions: None
- Question Groups: None
- Response Required: Yes
- Randomize Choice: No Choices
- Seconds to Answer: 0
- Seconds Until Next Button Becomes Active: 0

Breakdown of Each Trigger

Name	Type	Settings
1) 1 CPD > Probability = 100%	participantAction	false
2) 2 CPD > Probability = 100%	participantAction	false
3) 4 CPD > Probability = 75%	participantAction	false
4) 5 CPD > Probability = 60%	participantAction	false
5) 6 CPD > Probability = 50%	participantAction	false
6) 7 CPD > Probability = 43%	participantAction	false
7) 8 CPD > Probability = 38%	participantAction	false
8) 9 CPD > Probability = 33%	participantAction	false
9) 11 CPD > Probability = 27%	participantAction	false
10) 12 CPD > Probability = 25%	participantAction	false
11) 14 CPD > Probability = 21%	participantAction	false
12) 15 CPD > Probability = 20%	participantAction	false
13) 16 CPD > Probability = 19%	participantAction	false
14) 17 CPD > Probability = 18%	participantAction	false
15) 18 CPD > Probability = 17%	participantAction	false

16) 19 CPD > Probability = 16%	participantAction	false
17) >= 20 CPD > Probability = 15%	participantAction	false
18) 13 CPD > Probability = 23%	participantAction	false
19) 3 CPD > Probability = 100%	participantAction	false
20) 10 CPD > Probability = 30%	participantAction	false
21) PARTICIPANTACTION Trigger - test	participantAction	false

Question & Settings	Question Type	Variable Name	Global Variable Name
12) JUST BEFORE SMOKING: With how many people?	SINGLE_CHOICE	pssocial3	G:CF:pssocial3

- Display Conditions: ((If JUST BEFORE SMOKING: With others? IS Friends) OR (If JUST BEFORE SMOKING: With others? IS Acquaintances) OR (If JUST BEFORE SMOKING: With others? IS Family members) OR (If JUST BEFORE SMOKING: With others? IS Coworkers) OR (If JUST BEFORE SMOKING: With others? IS Romantic partner) OR (If JUST BEFORE SMOKING: With others? IS Unknown person))
- Question Groups: None
- Response Required: Yes
- Randomize Choice: No Choices
- Seconds to Answer: 0
- Seconds Until Next Button Becomes Active: 0

Breakdown of Each Trigger

Name	Type	Settings
1) 1 CPD > Probability = 100%	participantAction	false
2) 2 CPD > Probability = 100%	participantAction	false
3) 4 CPD > Probability = 75%	participantAction	false
4) 5 CPD > Probability = 60%	participantAction	false

5) 6 CPD > Probability = 50%	participantAction	false
6) 7 CPD > Probability = 43%	participantAction	false
7) 8 CPD > Probability = 38%	participantAction	false
8) 9 CPD > Probability = 33%	participantAction	false
9) 11 CPD > Probability = 27%	participantAction	false
10) 12 CPD > Probability = 25%	participantAction	false
11) 14 CPD > Probability = 21%	participantAction	false
12) 15 CPD > Probability = 20%	participantAction	false
13) 16 CPD > Probability = 19%	participantAction	false
14) 17 CPD > Probability = 18%	participantAction	false
15) 18 CPD > Probability = 17%	participantAction	false
16) 19 CPD > Probability = 16%	participantAction	false
17) >= 20 CPD > Probability = 15%	participantAction	false
18) 13 CPD > Probability = 23%	participantAction	false
19) 3 CPD > Probability = 100%	participantAction	false
20) 10 CPD > Probability = 30%	participantAction	false
21) PARTICIPANTACTION Trigger - test	participantAction	false

Question & Settings	Question Type	Variable Name	Global Variable Name
13) JUST BEFORE SMOKING: With how many people?	DROPDOWN	pssocial3_dropdown	G:CF:pssocial3_dropdown

- Display Conditions: (If JUST BEFORE SMOKING: With others? IS Friends) OR (If JUST BEFORE SMOKING: With others? IS Acquaintances) OR (If JUST BEFORE SMOKING: With others? IS Family members) OR (If JUST BEFORE SMOKING: With others? IS Coworkers) OR (If JUST BEFORE SMOKING: With others? IS Romantic partner) OR (If JUST BEFORE SMOKING: With others? IS Unknown person)
- Question Groups: None
- Response Required: Yes

- Randomize Choice: No Choices
- Seconds to Answer: 0
- Seconds Until Next Button Becomes Active: 0

Breakdown of Each Trigger

Name	Type	Settings
1) 1 CPD > Probability = 100%	participantAction	false
2) 2 CPD > Probability = 100%	participantAction	false
3) 4 CPD > Probability = 75%	participantAction	false
4) 5 CPD > Probability = 60%	participantAction	false
5) 6 CPD > Probability = 50%	participantAction	false
6) 7 CPD > Probability = 43%	participantAction	false
7) 8 CPD > Probability = 38%	participantAction	false
8) 9 CPD > Probability = 33%	participantAction	false
9) 11 CPD > Probability = 27%	participantAction	false
10) 12 CPD > Probability = 25%	participantAction	false
11) 14 CPD > Probability = 21%	participantAction	false
12) 15 CPD > Probability = 20%	participantAction	false
13) 16 CPD > Probability = 19%	participantAction	false
14) 17 CPD > Probability = 18%	participantAction	false
15) 18 CPD > Probability = 17%	participantAction	false
16) 19 CPD > Probability = 16%	participantAction	false
17) >= 20 CPD > Probability = 15%	participantAction	false
18) 13 CPD > Probability = 23%	participantAction	false

19) 3 CPD > Probability = 100%	participantAction	false
20) 10 CPD > Probability = 30%	participantAction	false
21) PARTICIPANTACTION Trigger - test	participantAction	false

Question & Settings	Question Type	Variable Name	Global Variable Name
14) JUST BEFORE SMOKING: Other people smoking cigarettes or using tobacco products?	SINGLE_CHOICE	pssocial4	G:CF:pssocial4

- Display Conditions: ((If JUST BEFORE SMOKING: With others? IS Friends) OR (If JUST BEFORE SMOKING: With others? IS Acquaintances) OR (If JUST BEFORE SMOKING: With others? IS Family members) OR (If JUST BEFORE SMOKING: With others? IS Coworkers) OR (If JUST BEFORE SMOKING: With others? IS Romantic partner) OR (If JUST BEFORE SMOKING: With others? IS Unknown person))
- Question Groups: None
- Response Required: Yes
- Randomize Choice: No Choices
- Seconds to Answer: 0
- Seconds Until Next Button Becomes Active: 0

Breakdown of Each Trigger

Name	Type	Settings
1) 1 CPD > Probability = 100%	participantAction	false
2) 2 CPD > Probability = 100%	participantAction	false
3) 4 CPD > Probability = 75%	participantAction	false
4) 5 CPD > Probability = 60%	participantAction	false
5) 6 CPD > Probability = 50%	participantAction	false
6) 7 CPD > Probability = 43%	participantAction	false
7) 8 CPD > Probability = 38%	participantAction	false

8) 9 CPD > Probability = 33%	participantAction	false
9) 11 CPD > Probability = 27%	participantAction	false
10) 12 CPD > Probability = 25%	participantAction	false
11) 14 CPD > Probability = 21%	participantAction	false
12) 15 CPD > Probability = 20%	participantAction	false
13) 16 CPD > Probability = 19%	participantAction	false
14) 17 CPD > Probability = 18%	participantAction	false
15) 18 CPD > Probability = 17%	participantAction	false
16) 19 CPD > Probability = 16%	participantAction	false
17) >= 20 CPD > Probability = 15%	participantAction	false
18) 13 CPD > Probability = 23%	participantAction	false
19) 3 CPD > Probability = 100%	participantAction	false
20) 10 CPD > Probability = 30%	participantAction	false
21) PARTICIPANTACTION Trigger - test	participantAction	false

Question & Settings	Question Type	Variable Name	Global Variable Name
15) JUST BEFORE SMOKING: Who was smoking cigarettes or using tobacco products?	MULTIPLE_CHOICE	pssocial5	G:CF:pssocial5

- Display Conditions: If JUST BEFORE SMOKING: Other people smoking cigarettes or using tobacco products? IS Yes
- Question Groups: None
- Response Required: Yes
- Randomize Choice: No Choices
- Seconds to Answer: 0
- Seconds Until Next Button Becomes Active: 0

Breakdown of Each Trigger

Name	Type	Settings
1) 1 CPD > Probability = 100%	participantAction	false
2) 2 CPD > Probability = 100%	participantAction	false
3) 4 CPD > Probability = 75%	participantAction	false
4) 5 CPD > Probability = 60%	participantAction	false
5) 6 CPD > Probability = 50%	participantAction	false
6) 7 CPD > Probability = 43%	participantAction	false
7) 8 CPD > Probability = 38%	participantAction	false
8) 9 CPD > Probability = 33%	participantAction	false
9) 11 CPD > Probability = 27%	participantAction	false
10) 12 CPD > Probability = 25%	participantAction	false
11) 14 CPD > Probability = 21%	participantAction	false
12) 15 CPD > Probability = 20%	participantAction	false
13) 16 CPD > Probability = 19%	participantAction	false
14) 17 CPD > Probability = 18%	participantAction	false
15) 18 CPD > Probability = 17%	participantAction	false
16) 19 CPD > Probability = 16%	participantAction	false
17) >= 20 CPD > Probability = 15%	participantAction	false
18) 13 CPD > Probability = 23%	participantAction	false
19) 3 CPD > Probability = 100%	participantAction	false
20) 10 CPD > Probability = 30%	participantAction	false
21) PARTICIPANTACTION Trigger - test	participantAction	false

Question & Settings	Question Type	Variable Name	Global Variable Name
16) JUST BEFORE SMOKING: How many people were smoking or using tobacco products?	SINGLE_CHOICE	pssocial6	G:CF:pssocial6

- Display Conditions: If JUST BEFORE SMOKING: Other people smoking cigarettes or using tobacco products? IS Yes
- Question Groups: None
- Response Required: Yes
- Randomize Choice: No Choices
- Seconds to Answer: 0
- Seconds Until Next Button Becomes Active: 0

Breakdown of Each Trigger

Name	Type	Settings
1) 1 CPD > Probability = 100%	participantAction	false
2) 2 CPD > Probability = 100%	participantAction	false
3) 4 CPD > Probability = 75%	participantAction	false
4) 5 CPD > Probability = 60%	participantAction	false
5) 6 CPD > Probability = 50%	participantAction	false
6) 7 CPD > Probability = 43%	participantAction	false
7) 8 CPD > Probability = 38%	participantAction	false
8) 9 CPD > Probability = 33%	participantAction	false
9) 11 CPD > Probability = 27%	participantAction	false
10) 12 CPD > Probability = 25%	participantAction	false
11) 14 CPD > Probability = 21%	participantAction	false
12) 15 CPD > Probability = 20%	participantAction	false

13) 16 CPD > Probability = 19%	participantAction	false
14) 17 CPD > Probability = 18%	participantAction	false
15) 18 CPD > Probability = 17%	participantAction	false
16) 19 CPD > Probability = 16%	participantAction	false
17) >= 20 CPD > Probability = 15%	participantAction	false
18) 13 CPD > Probability = 23%	participantAction	false
19) 3 CPD > Probability = 100%	participantAction	false
20) 10 CPD > Probability = 30%	participantAction	false
21) PARTICIPANTACTION Trigger - test	participantAction	false

Question & Settings	Question Type	Variable Name	Global Variable Name
17) JUST BEFORE SMOKING: How many people were smoking or using tobacco products?	DROPDOWN	pssocial6_dropdown	G:CF:pssocial6_dropdown

- Display Conditions: If JUST BEFORE SMOKING: Other people smoking cigarettes or using tobacco products? IS Yes
- Question Groups: None
- Response Required: Yes
- Randomize Choice: No Choices
- Seconds to Answer: 0
- Seconds Until Next Button Becomes Active: 0

Breakdown of Each Trigger

Name	Type	Settings
1) 1 CPD > Probability = 100%	participantAction	false
2) 2 CPD > Probability = 100%	participantAction	false

3) 4 CPD > Probability = 75%	participantAction	false
4) 5 CPD > Probability = 60%	participantAction	false
5) 6 CPD > Probability = 50%	participantAction	false
6) 7 CPD > Probability = 43%	participantAction	false
7) 8 CPD > Probability = 38%	participantAction	false
8) 9 CPD > Probability = 33%	participantAction	false
9) 11 CPD > Probability = 27%	participantAction	false
10) 12 CPD > Probability = 25%	participantAction	false
11) 14 CPD > Probability = 21%	participantAction	false
12) 15 CPD > Probability = 20%	participantAction	false
13) 16 CPD > Probability = 19%	participantAction	false
14) 17 CPD > Probability = 18%	participantAction	false
15) 18 CPD > Probability = 17%	participantAction	false
16) 19 CPD > Probability = 16%	participantAction	false
17) >= 20 CPD > Probability = 15%	participantAction	false
18) 13 CPD > Probability = 23%	participantAction	false
19) 3 CPD > Probability = 100%	participantAction	false
20) 10 CPD > Probability = 30%	participantAction	false
21) PARTICIPANTACTION Trigger - test	participantAction	false

Question & Settings	Question Type	Variable Name	Global Variable Name
18) JUST BEFORE SMOKING: What were you doing?	SINGLE_CHOICE	psactivity	G:CF:psactivity

- Display Conditions: None
- Question Groups: None

- Response Required: Yes
- Randomize Choice: No Choices
- Seconds to Answer: 0
- Seconds Until Next Button Becomes Active: 0

Breakdown of Each Trigger

Name	Type	Settings
1) 1 CPD > Probability = 100%	participantAction	false
2) 2 CPD > Probability = 100%	participantAction	false
3) 4 CPD > Probability = 75%	participantAction	false
4) 5 CPD > Probability = 60%	participantAction	false
5) 6 CPD > Probability = 50%	participantAction	false
6) 7 CPD > Probability = 43%	participantAction	false
7) 8 CPD > Probability = 38%	participantAction	false
8) 9 CPD > Probability = 33%	participantAction	false
9) 11 CPD > Probability = 27%	participantAction	false
10) 12 CPD > Probability = 25%	participantAction	false
11) 14 CPD > Probability = 21%	participantAction	false
12) 15 CPD > Probability = 20%	participantAction	false
13) 16 CPD > Probability = 19%	participantAction	false
14) 17 CPD > Probability = 18%	participantAction	false
15) 18 CPD > Probability = 17%	participantAction	false
16) 19 CPD > Probability = 16%	participantAction	false
17) >= 20 CPD > Probability = 15%	participantAction	false
18) 13 CPD > Probability = 23%	participantAction	false

19) 3 CPD > Probability = 100%	participantAction	false
20) 10 CPD > Probability = 30%	participantAction	false
21) PARTICIPANTACTION Trigger - test	participantAction	false

Question & Settings	Question Type	Variable Name	Global Variable Name
19) JUST BEFORE SMOKING: What other activities?	TEXT	psactivityother	G:CF:psactivityother

- Display Conditions: If JUST BEFORE SMOKING: What were you doing? IS Other activities
- Question Groups: None
- Response Required: Yes
- Randomize Choice: No Choices
- Seconds to Answer: 0
- Seconds Until Next Button Becomes Active: 0

Breakdown of Each Trigger

Name	Type	Settings
1) 1 CPD > Probability = 100%	participantAction	false
2) 2 CPD > Probability = 100%	participantAction	false
3) 4 CPD > Probability = 75%	participantAction	false
4) 5 CPD > Probability = 60%	participantAction	false
5) 6 CPD > Probability = 50%	participantAction	false
6) 7 CPD > Probability = 43%	participantAction	false
7) 8 CPD > Probability = 38%	participantAction	false
8) 9 CPD > Probability = 33%	participantAction	false
9) 11 CPD > Probability = 27%	participantAction	false

10) 12 CPD > Probability = 25%	participantAction	false
11) 14 CPD > Probability = 21%	participantAction	false
12) 15 CPD > Probability = 20%	participantAction	false
13) 16 CPD > Probability = 19%	participantAction	false
14) 17 CPD > Probability = 18%	participantAction	false
15) 18 CPD > Probability = 17%	participantAction	false
16) 19 CPD > Probability = 16%	participantAction	false
17) >= 20 CPD > Probability = 15%	participantAction	false
18) 13 CPD > Probability = 23%	participantAction	false
19) 3 CPD > Probability = 100%	participantAction	false
20) 10 CPD > Probability = 30%	participantAction	false
21) PARTICIPANTACTION Trigger - test	participantAction	false

Question & Settings	Question Type	Variable Name	Global Variable Name
20) Consume any of the following in last hour?	MULTIPLE_CHOICE	psconsume	G:CF:psconsume

- Display Conditions: None
- Question Groups: None
- Response Required: Yes
- Randomize Choice: No Choices
- Seconds to Answer: 0
- Seconds Until Next Button Becomes Active: 0

Breakdown of Each Trigger

Name	Type	Settings

1) 1 CPD > Probability = 100%	participantAction	false
2) 2 CPD > Probability = 100%	participantAction	false
3) 4 CPD > Probability = 75%	participantAction	false
4) 5 CPD > Probability = 60%	participantAction	false
5) 6 CPD > Probability = 50%	participantAction	false
6) 7 CPD > Probability = 43%	participantAction	false
7) 8 CPD > Probability = 38%	participantAction	false
8) 9 CPD > Probability = 33%	participantAction	false
9) 11 CPD > Probability = 27%	participantAction	false
10) 12 CPD > Probability = 25%	participantAction	false
11) 14 CPD > Probability = 21%	participantAction	false
12) 15 CPD > Probability = 20%	participantAction	false
13) 16 CPD > Probability = 19%	participantAction	false
14) 17 CPD > Probability = 18%	participantAction	false
15) 18 CPD > Probability = 17%	participantAction	false
16) 19 CPD > Probability = 16%	participantAction	false
17) >= 20 CPD > Probability = 15%	participantAction	false
18) 13 CPD > Probability = 23%	participantAction	false
19) 3 CPD > Probability = 100%	participantAction	false
20) 10 CPD > Probability = 30%	participantAction	false
21) PARTICIPANTACTION Trigger - test	participantAction	false

Question & Settings	Question Type	Variable Name	Global Variable Name
21) JUST BEFORE SMOKING: Intoxicated/drunk?	SINGLE_CHOICE	psintoxicated	G:CF:psintoxicated

- Display Conditions: None
- Question Groups: None
- Response Required: Yes
- Randomize Choice: No Choices
- Seconds to Answer: 0
- Seconds Until Next Button Becomes Active: 0

Breakdown of Each Trigger

Name	Type	Settings
1) 1 CPD > Probability = 100%	participantAction	false
2) 2 CPD > Probability = 100%	participantAction	false
3) 4 CPD > Probability = 75%	participantAction	false
4) 5 CPD > Probability = 60%	participantAction	false
5) 6 CPD > Probability = 50%	participantAction	false
6) 7 CPD > Probability = 43%	participantAction	false
7) 8 CPD > Probability = 38%	participantAction	false
8) 9 CPD > Probability = 33%	participantAction	false
9) 11 CPD > Probability = 27%	participantAction	false
10) 12 CPD > Probability = 25%	participantAction	false
11) 14 CPD > Probability = 21%	participantAction	false
12) 15 CPD > Probability = 20%	participantAction	false
13) 16 CPD > Probability = 19%	participantAction	false
14) 17 CPD > Probability = 18%	participantAction	false
15) 18 CPD > Probability = 17%	participantAction	false

16) 19 CPD > Probability = 16%	participantAction	false
17) >= 20 CPD > Probability = 15%	participantAction	false
18) 13 CPD > Probability = 23%	participantAction	false
19) 3 CPD > Probability = 100%	participantAction	false
20) 10 CPD > Probability = 30%	participantAction	false
21) PARTICIPANTACTION Trigger - test	participantAction	false

Question & Settings	Question Type	Variable Name	Global Variable Name
22) JUST BEFORE SMOKING: Saw any of the following?	MULTIPLE_CHOICE	psexposed	G:CF:psexposed

- Display Conditions: None
- Question Groups: None
- Response Required: Yes
- Randomize Choice: No Choices
- Seconds to Answer: 0
- Seconds Until Next Button Becomes Active: 0

Breakdown of Each Trigger

Name	Type	Settings
1) 1 CPD > Probability = 100%	participantAction	false
2) 2 CPD > Probability = 100%	participantAction	false
3) 4 CPD > Probability = 75%	participantAction	false
4) 5 CPD > Probability = 60%	participantAction	false
5) 6 CPD > Probability = 50%	participantAction	false
6) 7 CPD > Probability = 43%	participantAction	false

7) 8 CPD > Probability = 38%	participantAction	false
8) 9 CPD > Probability = 33%	participantAction	false
9) 11 CPD > Probability = 27%	participantAction	false
10) 12 CPD > Probability = 25%	participantAction	false
11) 14 CPD > Probability = 21%	participantAction	false
12) 15 CPD > Probability = 20%	participantAction	false
13) 16 CPD > Probability = 19%	participantAction	false
14) 17 CPD > Probability = 18%	participantAction	false
15) 18 CPD > Probability = 17%	participantAction	false
16) 19 CPD > Probability = 16%	participantAction	false
17) >= 20 CPD > Probability = 15%	participantAction	false
18) 13 CPD > Probability = 23%	participantAction	false
19) 3 CPD > Probability = 100%	participantAction	false
20) 10 CPD > Probability = 30%	participantAction	false
21) PARTICIPANTACTION Trigger - test	participantAction	false

Question & Settings	Question Type	Variable Name	Global Variable Name
23) SINCE THE LAST SURVEY: I felt discriminated against.	SINGLE_CHOICE	psdiscrimination1	G:CF:psdiscrimination1

- Display Conditions: None
- Question Groups: None
- Response Required: Yes
- Randomize Choice: No Choices
- Seconds to Answer: 0
- Seconds Until Next Button Becomes Active: 0

Breakdown of Each Trigger

Name	Type	Settings
1) 1 CPD > Probability = 100%	participantAction	false
2) 2 CPD > Probability = 100%	participantAction	false
3) 4 CPD > Probability = 75%	participantAction	false
4) 5 CPD > Probability = 60%	participantAction	false
5) 6 CPD > Probability = 50%	participantAction	false
6) 7 CPD > Probability = 43%	participantAction	false
7) 8 CPD > Probability = 38%	participantAction	false
8) 9 CPD > Probability = 33%	participantAction	false
9) 11 CPD > Probability = 27%	participantAction	false
10) 12 CPD > Probability = 25%	participantAction	false
11) 14 CPD > Probability = 21%	participantAction	false
12) 15 CPD > Probability = 20%	participantAction	false
13) 16 CPD > Probability = 19%	participantAction	false
14) 17 CPD > Probability = 18%	participantAction	false
15) 18 CPD > Probability = 17%	participantAction	false
16) 19 CPD > Probability = 16%	participantAction	false
17) >= 20 CPD > Probability = 15%	participantAction	false
18) 13 CPD > Probability = 23%	participantAction	false
19) 3 CPD > Probability = 100%	participantAction	false
20) 10 CPD > Probability = 30%	participantAction	false
21) PARTICIPANTACTION Trigger - test	participantAction	false

Question & Settings	Question Type	Variable Name	Global Variable Name
24) SINCE THE LAST SURVEY: What was the main reason (s) for the discrimination that you experienced?	MULTIPLE_CHOICE	psdiscrimination2	G:CF:psdiscrimination2

- Display Conditions: If SINCE THE LAST SURVEY: I felt discriminated against. IS Yes
- Question Groups: None
- Response Required: Yes
- Randomize Choice: No Choices
- Seconds to Answer: 0
- Seconds Until Next Button Becomes Active: 0

Breakdown of Each Trigger

Name	Type	Settings
1) 1 CPD > Probability = 100%	participantAction	false
2) 2 CPD > Probability = 100%	participantAction	false
3) 4 CPD > Probability = 75%	participantAction	false
4) 5 CPD > Probability = 60%	participantAction	false
5) 6 CPD > Probability = 50%	participantAction	false
6) 7 CPD > Probability = 43%	participantAction	false
7) 8 CPD > Probability = 38%	participantAction	false
8) 9 CPD > Probability = 33%	participantAction	false
9) 11 CPD > Probability = 27%	participantAction	false
10) 12 CPD > Probability = 25%	participantAction	false
11) 14 CPD > Probability = 21%	participantAction	false

12) 15 CPD > Probability = 20%	participantAction	false
13) 16 CPD > Probability = 19%	participantAction	false
14) 17 CPD > Probability = 18%	participantAction	false
15) 18 CPD > Probability = 17%	participantAction	false
16) 19 CPD > Probability = 16%	participantAction	false
17) >= 20 CPD > Probability = 15%	participantAction	false
18) 13 CPD > Probability = 23%	participantAction	false
19) 3 CPD > Probability = 100%	participantAction	false
20) 10 CPD > Probability = 30%	participantAction	false
21) PARTICIPANTACTION Trigger - test	participantAction	false

Question & Settings	Question Type	Variable Name	Global Variable Name
25) SINCE THE LAST SURVEY: Who discriminated against you?	MULTIPLE_CHOICE	psdiscrimination3	G:CF:psdiscrimination3

- Display Conditions: If SINCE THE LAST SURVEY: I felt discriminated against. IS Yes
- Question Groups: None
- Response Required: Yes
- Randomize Choice: No Choices
- Seconds to Answer: 0
- Seconds Until Next Button Becomes Active: 0

Breakdown of Each Trigger

Name	Type	Settings
1) 1 CPD > Probability = 100%	participantAction	false
2) 2 CPD > Probability = 100%	participantAction	false

3) 4 CPD > Probability = 75%	participantAction	false
4) 5 CPD > Probability = 60%	participantAction	false
5) 6 CPD > Probability = 50%	participantAction	false
6) 7 CPD > Probability = 43%	participantAction	false
7) 8 CPD > Probability = 38%	participantAction	false
8) 9 CPD > Probability = 33%	participantAction	false
9) 11 CPD > Probability = 27%	participantAction	false
10) 12 CPD > Probability = 25%	participantAction	false
11) 14 CPD > Probability = 21%	participantAction	false
12) 15 CPD > Probability = 20%	participantAction	false
13) 16 CPD > Probability = 19%	participantAction	false
14) 17 CPD > Probability = 18%	participantAction	false
15) 18 CPD > Probability = 17%	participantAction	false
16) 19 CPD > Probability = 16%	participantAction	false
17) >= 20 CPD > Probability = 15%	participantAction	false
18) 13 CPD > Probability = 23%	participantAction	false
19) 3 CPD > Probability = 100%	participantAction	false
20) 10 CPD > Probability = 30%	participantAction	false
21) PARTICIPANTACTION Trigger - test	participantAction	false

Survey Information for Daily Diary

- Name: Daily Diary
- Type: MOBILE

- Enable Survey End Screen: No
- Randomized Question Order: No
- Disable Review Question Screen: Yes
- Randomly Trigger a Single Group: No
- Number of Survey Triggers: 8
- Number of Questions: 34

Data Format

Variable Name	Format	Position	Description
Response ID	Character	1	Unique Response Identifier
User ID	Character	2	Unique Participant Identifier
Survey Started Date	Character	3	Date participant started to answer the survey
Survey Started Time	Character	4	Time participant started to answer the survey
Survey Submitted Date	Character	5	Date participant submitted survey
Survey Submitted Time	Character	6	Time participant submitted survey
Time Zone	Character	7	Identifies the time zone in UTC of the paricipant at the time of survey submission
Submission Location	Numeric	8	Location of the user at the time of response submission
Device OS	Character	9	Identifies the device operating system at the time of submission
Device OS Version	Character	10	Identifies the version device

			operating system at the time of submission
App Version	Numeric	11	Identifies the app version downloaded on the participant's device at the time of submission
Trigger Date	Character	12	Identifies the date that the trigger was prompted to participant
Trigger Time	Character	13	Identifies the time that the trigger was prompted to participant
Trigger Type	Character	14	Identifies the type of trigger that was prompted to participant
Trigger Name	Character	15	Identifies the name of trigger that was prompted to participant
Trigger Index	Numeric	16	Identifies the order of the trigger based on the parent prompt that the trigger was based on

Question Level Response Variables

Question	Variable Name	Format	Position	Question Type	Choices: Coded Value
YESTERDAY: How many cigarettes?	cigsyest1_dropdown	Character	17	DROPDOWN	- 0 - I did not smoke yesterday (not even a puff) : 0<br>-<br>1 : 1<br>- 2 : 2<br>- 3 : 3<br>- 4 : 4<br>- 5 : 5

					- 6 : 6<br>- 7 : 7<br>- 8 : 8<br>- 9 : 9<br>- 10 : 10<br>- 11 : 11<br>- 12 : 12<br>- 13 : 13<br>- 14 : 14<br>- 15 : 15<br>- 16 : 16<br>- 17 : 17<br>- 18 : 18<br>- 19 : 19<br>- 20 : 20<br>- 21 : 21<br>- 22 : 22<br>- 23 : 23<br>- 24 : 24<br>- 25 : 25<br>- 26 : 26<br>- 27 : 27<br>- 28 : 28<br>- 29 : 29<br>- 30 : 30<br>- 31+ : 31
YESTERDAY: How many cigarettes?	cigsyest1	Character	18	SINGLE_CHOICE	- 0 - I did not smoke yesterday (not even a puff) : 0<br>- 1 : 1<br>- 2-5 : 2<br>- 6-10 : 3

					- 11-15 : 4<br>- 16-20 : 5<br>- 21-30 : 6<br>- 31+ : 7
YESTERDAY: Did you use cigarillos?	cigarillosyest2	Character	19	SINGLE_CHOICE	- Yes : 1<br>- No : 0
YESTERDAY: How many cigarillos?	cigarillosyest1_dropdown	Character	20	DROPDOWN	- 0 - I did not smoke cigarillos (not even a puff) : 0<br>-<br>1 : 1<br>- 2 : 2<br>- 3 : 3<br>- 4 : 4<br>- 5 : 5<br>- 6 : 6<br>- 7 : 7<br>- 8 : 8<br>- 9 : 9<br>- 10 : 10<br>- 11 : 11<br>- 12 : 12<br>- 13 : 13<br>- 14 : 14<br>- 15 : 15<br>- 16 : 16<br>- 17 : 17<br>- 18 : 18<br>- 19 : 19<br>- 20 : 20<br>- 21 : 21<br>- 22 : 22

					- 23 : 23<br>- 24 : 24<br>- 25 : 25<br>- 26 : 26<br>- 27 : 27<br>- 28 : 28<br>- 29 : 29<br>- 30 : 30<br>- 31+ : 31
YESTERDAY: How many cigarillos?	cigarillosyest1	Character	21	SINGLE_CHOICE	- 0 - I did not use cigarillos yesterday : 0<br>- 1 : 1<br>- 2-5 : 2<br>- 6-10 : 3<br>- 11-15 : 4<br>- 16-20 : 5<br>- 21-30 : 6<br>- 31+ : 7
YESTERDAY: Did you use Juul or other pod e-cigarette?	juulyest1	Character	22	SINGLE_CHOICE	- Yes : 1<br>- No : 0
YESTERDAY: How many times Juul or other pod e-cigarette?	juulyest2_dropdown	Character	23	DROPDOWN	-<br>1 : 1<br>- 2 : 2<br>- 3 : 3<br>- 4 : 4<br>- 5 : 5<br>- 6 : 6<br>- 7 : 7<br>- 8 : 8<br>- 9 : 9<br>- 10 : 10<br>- 11 : 11

					- 12 : 12<br>- 13 : 13<br>- 14 : 14<br>- 15 : 15<br>- 16 : 16<br>- 17 : 17<br>- 18 : 18<br>- 19 : 19<br>- 20 : 20<br>- 21 : 21<br>- 22 : 22<br>- 23 : 23<br>- 24 : 24<br>- 25 : 25<br>- 26 : 26<br>- 27 : 27<br>- 28 : 28<br>- 29 : 29<br>- 30 : 30<br>- 31+ : 31
YESTERDAY: How many times Juul or other pod e-cigarette?	juulyest2	Character	24	SINGLE_CHOICE	- 1 : 1<br>- 2-5 : 2<br>- 6-10 : 3<br>- 11-15 : 4<br>- 16-20 : 5<br>- 21-30 : 6<br>- 31+ : 7
YESTERDAY: Did you use another type of e-cigarette (e.g., vape pen, tank)?	vapeyest1	Character	25	SINGLE_CHOICE	- Yes : 1<br>- No : 0
YESTERDAY: How many times other type e-cigarette (e.g., vape pen,	vapeyest2_dropdown	Character	26	DROPDOWN	-<br>1 : 1

tank)?					- 2 : 2<br>- 3 : 3<br>- 4 : 4<br>- 5 : 5<br>- 6 : 6<br>- 7 : 7<br>- 8 : 8<br>- 9 : 9<br>- 10 : 10<br>- 11 : 11<br>- 12 : 12<br>- 13 : 13<br>- 14 : 14<br>- 15 : 15<br>- 16 : 16<br>- 17 : 17<br>- 18 : 18<br>- 19 : 19<br>- 20 : 20<br>- 21 : 21<br>- 22 : 22<br>- 23 : 23<br>- 24 : 24<br>- 25 : 25<br>- 26 : 26<br>- 27 : 27<br>- 28 : 28<br>- 29 : 29<br>- 30 : 30<br>- 31+ : 31
YESTERDAY: How many times other type e-cigarette (e.g., vape pen,	vapeyest2	Character	27	SINGLE_CHOICE	- 1 : 1<br>- 2-5 : 2

tank)?					- 6-10 : 3<br>- 11-15 : 4<br>- 16-20 : 5<br>- 21-30 : 6<br>- 31+ : 7
Did your e-cigarette/vape contain nicotine YESTERDAY?	vapeyest3	Character	28	SINGLE_CHOICE	- Yes : 1<br>- No : 0
YESTERDAY: Did you use any other tobacco or nicotine product?	tobaccoyest1	Character	29	SINGLE_CHOICE	- Yes : 1<br>- No : 0
Which tobacco or nicotine product?	tobaccoyest2	Character	30	MULTIPLE_CHOICE	- Smokeless tobacco : 1<br>- Hookah : 2<br>- Other : 3
Which tobacco or nicotine product?	tobaccoyest3	Character	31	TEXT	
YESTERDAY: Did you use a marijuana or cannabis product?	cannabisyest1	Character	32	SINGLE_CHOICE	- Yes : 1<br>- No : 0
How many times marijuana or cannabis product?	cannabisyest2	Character	33	SINGLE_CHOICE	- 1 : 1<br>- 2 : 2<br>- 3 : 3<br>- 4 : 4<br>- 5 : 5<br>- 6 : 6<br>- 7+ : 7
YESTERDAY: How did you use marijuana or cannabis?	cannabisyest3	Character	34	MULTIPLE_CHOICE	- Smoked (pipe, joint, blunt, etc.) : 1<br>- Vape (vaporizer) : 2<br>- Edible (candy, cookie, etc.) : 3<br>- Dab or other concentrate : 4<br>- Other : 5

YESTERDAY: How much marijuana did you personally use? (Do NOT include other forms of cannabis you may use (such as concentrates))	cannabisyest4	Character	35	SINGLE_CHOICE	- Did not use marijuana : 1<br>- 0.125 grams or less<br>- 0.25 grams : 2<br>- 0.5 grams : 3<br>- 0.75 grams : 4<br>- 1 gram or more : 5
YESTERDAY: Did you combine tobacco and marijuana or cannabis (e.g., blunt, spliff, mixing and smoking in a pipe or bong)?	combineyset1	Character	36	SINGLE_CHOICE	- Yes : 1<br>- No : 0
YESTERDAY: How did you combine tobacco and marijuana or cannabis?	combineyest2	Character	37	MULTIPLE_CHOIC E	- Blunt : 1<br>- Spliff : 2<br>- Mixing and smoking (e.g., in pipe or bong) : 3<br>- Other : 4
YESTERDAY: Did you drink any alcohol?	alcoholyest1	Character	38	SINGLE_CHOICE	- Yes : 1<br>- No : 0
YESTERDAY: Number of alcoholic drinks?	alcoholyest2	Character	39	SINGLE_CHOICE	- 1 : 1<br>- 2 : 2<br>- 3 : 3<br>- 4 : 4<br>- 5 : 5<br>- 6 : 6<br>- 7+ : 7
YESTERDAY: While drinking ALCOHOL, did you also use CIGARETTES?	combineyest3	Character	40	SINGLE_CHOICE	- Yes : 1<br>- No : 0
YESTERDAY: While drinking ALCOHOL, did you also use E-CIGS/ VAPES with nicotine?	combineyest4	Character	41	SINGLE_CHOICE	- Yes : 1<br>- No : 0

YESTERDAY: While using CANNABIS, did you also use CIGARETTES?	combineyest5	Character	42	SINGLE_CHOICE	- Yes : 1<br>- No : 0
YESTERDAY: While using CANNABIS, did you also use E-CIGS/VAPES with nicotine?	combineyest6	Character	43	SINGLE_CHOICE	- Yes : 1<br>- No : 0
YESTERDAY: How many of your CIGARETTES did you smoke while drinking ALCOHOL?	combineyest7	Character	44	SINGLE_CHOICE	- None of them : 1<br>- Some of them : 2<br>- Most of them : 3<br>- All of them : 4
YESTERDAY: How many of your CIGARETTES did you smoke while using CANNABIS?	combineyest8	Character	45	SINGLE_CHOICE	- None of them : 1<br>- Some of them : 2<br>- Most of them : 3<br>- All of them : 4
YESTERDAY: How much of your E-CIG/VAPE use with nicotine was while drinking ALCOHOL?	combineyest9	Character	46	SINGLE_CHOICE	- None of it : 1<br>- Some of it : 2<br>- Most of it : 3<br>- All of it : 4
YESTERDAY: How much of your E-CIG/VAPE use with nicotine was while using CANNABIS?	combineyest10	Character	47	SINGLE_CHOICE	- None of it : 1<br>- Some of it : 2<br>- Most of it : 3<br>- All of it : 4
YESTERDAY: I felt discriminated against.	discriminationyest1	Character	48	SINGLE_CHOICE	- Yes : 1<br>- No : 0
YESTERDAY: What was the main reason(s) for the discrimination that you experienced?	discriminationyest2	Character	49	MULTIPLE_CHOICE	- Your age : 1<br>- Your gender : 2<br>- Your race : 3<br>- Your ethnicity or nationality : 4<br>- Your religion : 5<br>- Your height or weight : 6

					- Some other aspect of your appearance : 7<br>- A physical disability : 8<br>- Your sexual orientation : 9<br>- Being a smoker : 10<br>- Being poor : 11<br>- Other : 12
YESTERDAY: Who discriminated against you? Check all that apply	discriminationyest3	Character	50	MULTIPLE_CHOICE	- Family member : 1<br>- Romantic partner : 2<br>- Stranger : 4<br>- Acquaintance : 5<br>- Friend : 6<br>- Employer : 7<br>- Other : 8

Breakdown of Each Question

Question & Settings	Question Type	Variable Name	Global Variable Name
1) YESTERDAY: How many cigarettes?	DROPDOWN	cigsyest1_dropdown	G:DD:cigsyest1_dropdown

- Display Conditions: None
- Question Groups: None
- Response Required: Yes
- Randomize Choice: No Choices
- Seconds to Answer: 0
- Seconds Until Next Button Becomes Active: 0

Breakdown of Each Trigger

Name	Type	Settings
1)	ONCE	Runs from 2024-04-08T11:12:00 UNTIL 2025-04-08T00:00:00.000Z
2)	ONCE	Runs from 2024-09-27T13:26:00 UNTIL 2025-09-27T00:00:00.000Z
3)	ONCE	Runs from 2024-09-27T13:27:00 UNTIL 2025-09-27T00:00:00.000Z
4) Daily Diary	schedule	false
5) Daily Diary	schedule	false
6) Daily Diary - intervention group (training phrase)	schedule	false
7) Daily Diary - intervention group (intervention phrase)	schedule	false
8) Daily Diary - test geofences group	schedule	false

Question & Settings	Question Type	Variable Name	Global Variable Name
2) YESTERDAY: How many cigarettes?	SINGLE_CHOICE	cigsyest1	G:DD: cigsyest1

- Display Conditions: None
- Question Groups: None
- Response Required: Yes
- Randomize Choice: No Choices
- Seconds to Answer: 0
- Seconds Until Next Button Becomes Active: 0

Breakdown of Each Trigger

Name	Type	Settings
1)	ONCE	Runs from 2024-04-08T11:12:00 UNTIL 2025-04-08T00:00:00.000Z
2)	ONCE	Runs from 2024-09-27T13:26:00 UNTIL 2025-09-27T00:00:00.000Z
3)	ONCE	Runs from 2024-09-27T13:27:00 UNTIL 2025-09-27T00:00:00.000Z
4) Daily Diary	schedule	false
5) Daily Diary	schedule	false
6) Daily Diary - intervention group (training phrase)	schedule	false
7) Daily Diary - intervention group (intervention phrase)	schedule	false
8) Daily Diary - test geofences group	schedule	false

Question & Settings	Question Type	Variable Name	Global Variable Name
3) YESTERDAY: Did you use cigarillos?	SINGLE_CHOICE	cigarillosyest2	G:DD:cigarillosyest2

- Display Conditions: None
- Question Groups: None
- Response Required: Yes
- Randomize Choice: No Choices
- Seconds to Answer: 0
- Seconds Until Next Button Becomes Active: 0

Breakdown of Each Trigger

Name	Type	Settings
1)	ONCE	Runs from 2024-04-08T11:12:00 UNTIL 2025-04-08T00:00:00.000Z
2)	ONCE	Runs from 2024-09-27T13:26:00 UNTIL 2025-09-27T00:00:00.000Z
3)	ONCE	Runs from 2024-09-27T13:27:00 UNTIL 2025-09-27T00:00:00.000Z
4) Daily Diary	schedule	false
5) Daily Diary	schedule	false
6) Daily Diary - intervention group (training phrase)	schedule	false
7) Daily Diary - intervention group (intervention phrase)	schedule	false
8) Daily Diary - test geofences group	schedule	false

Question & Settings	Question Type	Variable Name	Global Variable Name
4) YESTERDAY: How many cigarillos?	DROPDOWN	cigarillosyest1_dropdown	G:DD:cigarillosyest1_dropdown

- Display Conditions: If YESTERDAY: Did you use cigarillos? IS Yes
- Question Groups: None
- Response Required: Yes
- Randomize Choice: No Choices
- Seconds to Answer: 0
- Seconds Until Next Button Becomes Active: 0

Breakdown of Each Trigger

Name	Type	Settings
1)	ONCE	Runs from 2024-04-08T11:12:00 UNTIL 2025-04-08T00:00:00.000Z
2)	ONCE	Runs from 2024-09-27T13:26:00 UNTIL 2025-09-27T00:00:00.000Z
3)	ONCE	Runs from 2024-09-27T13:27:00 UNTIL 2025-09-27T00:00:00.000Z
4) Daily Diary	schedule	false
5) Daily Diary	schedule	false
6) Daily Diary - intervention group (training phrase)	schedule	false
7) Daily Diary - intervention group (intervention phrase)	schedule	false
8) Daily Diary - test geofences group	schedule	false

Question & Settings	Question Type	Variable Name	Global Variable Name
5) YESTERDAY: How many cigarillos?	SINGLE_CHOICE	cigarillosyest1	G:DD:cigarillosyest1

- Display Conditions: None
- Question Groups: None
- Response Required: Yes
- Randomize Choice: No Choices
- Seconds to Answer: 0
- Seconds Until Next Button Becomes Active: 0

Breakdown of Each Trigger

Name	Type	Settings
1)	ONCE	Runs from 2024-04-08T11:12:00 UNTIL 2025-04-08T00:00:00.000Z
2)	ONCE	Runs from 2024-09-27T13:26:00 UNTIL 2025-09-27T00:00:00.000Z
3)	ONCE	Runs from 2024-09-27T13:27:00 UNTIL 2025-09-27T00:00:00.000Z
4) Daily Diary	schedule	false
5) Daily Diary	schedule	false
6) Daily Diary - intervention group (training phrase)	schedule	false
7) Daily Diary - intervention group (intervention phrase)	schedule	false
8) Daily Diary - test geofences group	schedule	false

Question & Settings	Question Type	Variable Name	Global Variable Name
6) YESTERDAY: Did you use Juul or other pod e-cigarette?	SINGLE_CHOICE	juulyest1	G:DD:juulyest1

- Display Conditions: None
- Question Groups: None
- Response Required: Yes
- Randomize Choice: No Choices
- Seconds to Answer: 0
- Seconds Until Next Button Becomes Active: 0

Breakdown of Each Trigger

Name	Type	Settings
1)	ONCE	Runs from 2024-04-08T11:12:00 UNTIL 2025-04-08T00:00:00.000Z
2)	ONCE	Runs from 2024-09-27T13:26:00 UNTIL 2025-09-27T00:00:00.000Z
3)	ONCE	Runs from 2024-09-27T13:27:00 UNTIL 2025-09-27T00:00:00.000Z
4) Daily Diary	schedule	false
5) Daily Diary	schedule	false
6) Daily Diary - intervention group (training phrase)	schedule	false
7) Daily Diary - intervention group (intervention phrase)	schedule	false
8) Daily Diary - test geofences group	schedule	false

Question & Settings	Question Type	Variable Name	Global Variable Name
7) YESTERDAY: How many times Juul or other pod e-cigarette?	DROPDOWN	juulyest2_dropdown	G:DD:juulyest2_dropdown

- Display Conditions: If YESTERDAY: Did you use Juul or other pod e-cigarette? IS Yes
- Question Groups: None
- Response Required: Yes
- Randomize Choice: No Choices
- Seconds to Answer: 0
- Seconds Until Next Button Becomes Active: 0

Breakdown of Each Trigger

Name	Type	Settings
1)	ONCE	Runs from 2024-04-08T11:12:00 UNTIL 2025-04-08T00:00:00.000Z
2)	ONCE	Runs from 2024-09-27T13:26:00 UNTIL 2025-09-27T00:00:00.000Z
3)	ONCE	Runs from 2024-09-27T13:27:00 UNTIL 2025-09-27T00:00:00.000Z
4) Daily Diary	schedule	false
5) Daily Diary	schedule	false
6) Daily Diary - intervention group (training phrase)	schedule	false
7) Daily Diary - intervention group (intervention phrase)	schedule	false
8) Daily Diary - test geofences group	schedule	false

Question & Settings	Question Type	Variable Name	Global Variable Name
8) YESTERDAY: How many times Juul or other pod e-cigarette?	SINGLE_CHOICE	juulyest2	G:DD:juulyest2

- Display Conditions: If YESTERDAY: Did you use Juul or other pod e-cigarette? IS Yes
- Question Groups: None
- Response Required: Yes
- Randomize Choice: No Choices
- Seconds to Answer: 0
- Seconds Until Next Button Becomes Active: 0

Breakdown of Each Trigger

Name	Type	Settings
1)	ONCE	Runs from 2024-04-08T11:12:00 UNTIL 2025-04-08T00:00:00.000Z
2)	ONCE	Runs from 2024-09-27T13:26:00 UNTIL 2025-09-27T00:00:00.000Z
3)	ONCE	Runs from 2024-09-27T13:27:00 UNTIL 2025-09-27T00:00:00.000Z
4) Daily Diary	schedule	false
5) Daily Diary	schedule	false
6) Daily Diary - intervention group (training phrase)	schedule	false
7) Daily Diary - intervention group (intervention phrase)	schedule	false
8) Daily Diary - test geofences group	schedule	false

Question & Settings	Question Type	Variable Name	Global Variable Name
9) YESTERDAY: Did you use another type of e-cigarette (e.g., vape pen, tank)?	SINGLE_CHOICE	vapeyest1	G:DD:vapeyest1

- Display Conditions: None
- Question Groups: None
- Response Required: Yes
- Randomize Choice: No Choices
- Seconds to Answer: 0
- Seconds Until Next Button Becomes Active: 0

Breakdown of Each Trigger

Name	Type	Settings
1)	ONCE	Runs from 2024-04-08T11:12:00 UNTIL 2025-04-08T00:00:00.000Z
2)	ONCE	Runs from 2024-09-27T13:26:00 UNTIL 2025-09-27T00:00:00.000Z
3)	ONCE	Runs from 2024-09-27T13:27:00 UNTIL 2025-09-27T00:00:00.000Z
4) Daily Diary	schedule	false
5) Daily Diary	schedule	false
6) Daily Diary - intervention group (training phrase)	schedule	false
7) Daily Diary - intervention group (intervention phrase)	schedule	false
8) Daily Diary - test geofences group	schedule	false

Question & Settings	Question Type	Variable Name	Global Variable Name
10) YESTERDAY: How many times other type e-cigarette (e.g., vape pen, tank)?	DROPDOWN	vapeyest2_dropdown	G:DD:vapeyest2_dropdown

- Display Conditions: If YESTERDAY: Did you use Juul or other pod e-cigarette? IS Yes
- Question Groups: None
- Response Required: Yes
- Randomize Choice: No Choices
- Seconds to Answer: 0
- Seconds Until Next Button Becomes Active: 0

Breakdown of Each Trigger

Name	Type	Settings
1)	ONCE	Runs from 2024-04-08T11:12:00 UNTIL 2025-04-08T00:00:00.000Z
2)	ONCE	Runs from 2024-09-27T13:26:00 UNTIL 2025-09-27T00:00:00.000Z
3)	ONCE	Runs from 2024-09-27T13:27:00 UNTIL 2025-09-27T00:00:00.000Z
4) Daily Diary	schedule	false
5) Daily Diary	schedule	false
6) Daily Diary - intervention group (training phrase)	schedule	false
7) Daily Diary - intervention group (intervention phrase)	schedule	false
8) Daily Diary - test geofences group	schedule	false

Question & Settings	Question Type	Variable Name	Global Variable Name
11) YESTERDAY: How many times other type e-cigarette (e.g., vape pen, tank)?	SINGLE_CHOICE	vapeyest2	G:DD:vapeyest2

- Display Conditions: If YESTERDAY: Did you use another type of e-cigarette (e.g., vape pen, tank)? IS Yes
- Question Groups: None
- Response Required: Yes
- Randomize Choice: No Choices
- Seconds to Answer: 0
- Seconds Until Next Button Becomes Active: 0

Breakdown of Each Trigger

Name	Type	Settings
1)	ONCE	Runs from 2024-04-08T11:12:00 UNTIL 2025-04-08T00:00:00.000Z
2)	ONCE	Runs from 2024-09-27T13:26:00 UNTIL 2025-09-27T00:00:00.000Z
3)	ONCE	Runs from 2024-09-27T13:27:00 UNTIL 2025-09-27T00:00:00.000Z
4) Daily Diary	schedule	false
5) Daily Diary	schedule	false
6) Daily Diary - intervention group (training phrase)	schedule	false
7) Daily Diary - intervention group (intervention phrase)	schedule	false
8) Daily Diary - test geofences group	schedule	false

Question & Settings	Question Type	Variable Name	Global Variable Name
12) Did your e-cigarette/vape contain nicotine YESTERDAY?	SINGLE_CHOICE	vapeyest3	G:DD:vapeyest3

- Display Conditions: (If YESTERDAY: Did you use Juul or other pod e-cigarette? IS Yes) OR (If YESTERDAY: Did you use another type of e-cigarette (e.g., vape pen, tank)? IS Yes)
- Question Groups: None
- Response Required: Yes
- Randomize Choice: No Choices
- Seconds to Answer: 0
- Seconds Until Next Button Becomes Active: 0

Breakdown of Each Trigger

Name	Type	Settings
1)	ONCE	Runs from 2024-04-08T11:12:00 UNTIL 2025-04-08T00:00:00.000Z
2)	ONCE	Runs from 2024-09-27T13:26:00 UNTIL 2025-09-27T00:00:00.000Z
3)	ONCE	Runs from 2024-09-27T13:27:00 UNTIL 2025-09-27T00:00:00.000Z
4) Daily Diary	schedule	false
5) Daily Diary	schedule	false
6) Daily Diary - intervention group (training phrase)	schedule	false
7) Daily Diary - intervention group (intervention phrase)	schedule	false
8) Daily Diary - test geofences group	schedule	false

Question & Settings	Question Type	Variable Name	Global Variable Name
13) YESTERDAY: Did you use any other tobacco or nicotine product?	SINGLE_CHOICE	tobaccoyest1	G:DD:tobaccoyest1

- Display Conditions: None
- Question Groups: None
- Response Required: Yes
- Randomize Choice: No Choices
- Seconds to Answer: 0
- Seconds Until Next Button Becomes Active: 0

Breakdown of Each Trigger

Name	Type	Settings
1)	ONCE	Runs from 2024-04-08T11:12:00 UNTIL 2025-04-08T00:00:00.000Z
2)	ONCE	Runs from 2024-09-27T13:26:00 UNTIL 2025-09-27T00:00:00.000Z
3)	ONCE	Runs from 2024-09-27T13:27:00 UNTIL 2025-09-27T00:00:00.000Z
4) Daily Diary	schedule	false
5) Daily Diary	schedule	false
6) Daily Diary - intervention group (training phrase)	schedule	false
7) Daily Diary - intervention group (intervention phrase)	schedule	false
8) Daily Diary - test geofences group	schedule	false

Question & Settings	Question Type	Variable Name	Global Variable Name
14) Which tobacco or nicotine product?	MULTIPLE_CHOICE	tobaccoyest2	G:DD:tobaccoyest2

- Display Conditions: If YESTERDAY: Did you use any other tobacco or nicotine product? IS Yes
- Question Groups: None
- Response Required: Yes
- Randomize Choice: No Choices
- Seconds to Answer: 0
- Seconds Until Next Button Becomes Active: 0

Breakdown of Each Trigger

Name	Type	Settings
1)	ONCE	Runs from 2024-04-08T11:12:00 UNTIL 2025-04-08T00:00:00.000Z
2)	ONCE	Runs from 2024-09-27T13:26:00 UNTIL 2025-09-27T00:00:00.000Z
3)	ONCE	Runs from 2024-09-27T13:27:00 UNTIL 2025-09-27T00:00:00.000Z
4) Daily Diary	schedule	false
5) Daily Diary	schedule	false
6) Daily Diary - intervention group (training phrase)	schedule	false
7) Daily Diary - intervention group (intervention phrase)	schedule	false
8) Daily Diary - test geofences group	schedule	false

Question & Settings	Question Type	Variable Name	Global Variable Name
15) Which tobacco or nicotine product?	TEXT	tobaccoyest3	G:DD:tobaccoyest3

- Display Conditions: If Which tobacco or nicotine product? IS Other
- Question Groups: None
- Response Required: Yes
- Randomize Choice: No Choices
- Seconds to Answer: 0
- Seconds Until Next Button Becomes Active: 0

Breakdown of Each Trigger

Name	Type	Settings
1)	ONCE	Runs from 2024-04-08T11:12:00 UNTIL 2025-04-08T00:00:00.000Z
2)	ONCE	Runs from 2024-09-27T13:26:00 UNTIL 2025-09-27T00:00:00.000Z
3)	ONCE	Runs from 2024-09-27T13:27:00 UNTIL 2025-09-27T00:00:00.000Z
4) Daily Diary	schedule	false
5) Daily Diary	schedule	false
6) Daily Diary - intervention group (training phrase)	schedule	false
7) Daily Diary - intervention group (intervention phrase)	schedule	false
8) Daily Diary - test geofences group	schedule	false

Question & Settings	Question Type	Variable Name	Global Variable Name
16) YESTERDAY: Did you use a marijuana or cannabis product?	SINGLE_CHOICE	cannabisyest1	G:DD:cannabisyest1

- Display Conditions: None
- Question Groups: None
- Response Required: Yes
- Randomize Choice: No Choices
- Seconds to Answer: 0
- Seconds Until Next Button Becomes Active: 0

Breakdown of Each Trigger

Name	Type	Settings
1)	ONCE	Runs from 2024-04-08T11:12:00 UNTIL 2025-04-08T00:00:00.000Z
2)	ONCE	Runs from 2024-09-27T13:26:00 UNTIL 2025-09-27T00:00:00.000Z
3)	ONCE	Runs from 2024-09-27T13:27:00 UNTIL 2025-09-27T00:00:00.000Z
4) Daily Diary	schedule	false
5) Daily Diary	schedule	false
6) Daily Diary - intervention group (training phrase)	schedule	false
7) Daily Diary - intervention group (intervention phrase)	schedule	false
8) Daily Diary - test geofences group	schedule	false

Question & Settings	Question Type	Variable Name	Global Variable Name
17) How many times marijuana or cannabis product?	SINGLE_CHOICE	cannabisyest2	G:DD:cannabisyest2

- Display Conditions: If YESTERDAY: Did you use a marijuana or cannabis product? IS Yes
- Question Groups: None
- Response Required: Yes
- Randomize Choice: No Choices
- Seconds to Answer: 0
- Seconds Until Next Button Becomes Active: 0

Breakdown of Each Trigger

Name	Type	Settings
1)	ONCE	Runs from 2024-04-08T11:12:00 UNTIL 2025-04-08T00:00:00.000Z
2)	ONCE	Runs from 2024-09-27T13:26:00 UNTIL 2025-09-27T00:00:00.000Z
3)	ONCE	Runs from 2024-09-27T13:27:00 UNTIL 2025-09-27T00:00:00.000Z
4) Daily Diary	schedule	false
5) Daily Diary	schedule	false
6) Daily Diary - intervention group (training phrase)	schedule	false
7) Daily Diary - intervention group (intervention phrase)	schedule	false
8) Daily Diary - test geofences group	schedule	false

Question & Settings	Question Type	Variable Name	Global Variable Name
18) YESTERDAY: How did you use marijuana or cannabis?	MULTIPLE_CHOICE	cannabisyest3	G:DD:cannabisyest3

- Display Conditions: If YESTERDAY: Did you use a marijuana or cannabis product? IS Yes
- Question Groups: None
- Response Required: Yes
- Randomize Choice: No Choices
- Seconds to Answer: 0
- Seconds Until Next Button Becomes Active: 0

Breakdown of Each Trigger

Name	Type	Settings
1)	ONCE	Runs from 2024-04-08T11:12:00 UNTIL 2025-04-08T00:00:00.000Z
2)	ONCE	Runs from 2024-09-27T13:26:00 UNTIL 2025-09-27T00:00:00.000Z
3)	ONCE	Runs from 2024-09-27T13:27:00 UNTIL 2025-09-27T00:00:00.000Z
4) Daily Diary	schedule	false
5) Daily Diary	schedule	false
6) Daily Diary - intervention group (training phrase)	schedule	false
7) Daily Diary - intervention group (intervention phrase)	schedule	false
8) Daily Diary - test geofences group	schedule	false

Question & Settings	Question Type	Variable Name	Global Variable Name
19) YESTERDAY: How much marijuana did you personally use? (Do NOT include other forms of cannabis you may use (such as concentrates))	SINGLE_CHOICE	cannabisyest4	G:DD:cannabisyest4

- Display Conditions: (If YESTERDAY: Did you use a marijuana or cannabis product? IS Yes) AND ((If YESTERDAY: How did you use marijuana or cannabis? IS Smoked (pipe, joint, blunt, etc.)) OR (If YESTERDAY: How did you use marijuana or cannabis? IS Vape (vaporizer)))
- Question Groups: None
- Response Required: Yes
- Randomize Choice: No Choices
- Seconds to Answer: 0
- Seconds Until Next Button Becomes Active: 0

Breakdown of Each Trigger

Name	Type	Settings
1)	ONCE	Runs from 2024-04-08T11:12:00 UNTIL 2025-04-08T00:00:00.000Z
2)	ONCE	Runs from 2024-09-27T13:26:00 UNTIL 2025-09-27T00:00:00.000Z
3)	ONCE	Runs from 2024-09-27T13:27:00 UNTIL 2025-09-27T00:00:00.000Z
4) Daily Diary	schedule	false
5) Daily Diary	schedule	false
6) Daily Diary - intervention group (training phrase)	schedule	false
7) Daily Diary - intervention group (intervention phrase)	schedule	false
8) Daily Diary - test geofences group	schedule	false

Question & Settings	Question Type	Variable Name	Global Variable Name
20) YESTERDAY: Did you combine tobacco and marijuana or cannabis (e.g., blunt, spliff, mixing and smoking in a pipe or bong)?	SINGLE_CHOICE	combineyset1	G:DD:combineyset1

- Display Conditions: (If YESTERDAY: Did you use a marijuana or cannabis product? IS Yes) AND (If YESTERDAY: How much marijuana did you personally use? (Do NOT include other forms of cannabis you may use (such as concentrates)) IS NOT Did not use marijuana)
- Question Groups: None
- Response Required: Yes

- Randomize Choice: No Choices
- Seconds to Answer: 0
- Seconds Until Next Button Becomes Active: 0

Breakdown of Each Trigger

Name	Type	Settings
1)	ONCE	Runs from 2024-04-08T11:12:00 UNTIL 2025-04-08T00:00:00.000Z
2)	ONCE	Runs from 2024-09-27T13:26:00 UNTIL 2025-09-27T00:00:00.000Z
3)	ONCE	Runs from 2024-09-27T13:27:00 UNTIL 2025-09-27T00:00:00.000Z
4) Daily Diary	schedule	false
5) Daily Diary	schedule	false
6) Daily Diary - intervention group (training phrase)	schedule	false
7) Daily Diary - intervention group (intervention phrase)	schedule	false
8) Daily Diary - test geofences group	schedule	false

Question & Settings	Question Type	Variable Name	Global Variable Name
21) YESTERDAY: How did you combine tobacco and marijuana or cannabis?	MULTIPLE_CHOICE	combineyest2	G:DD:combineyest2

- Display Conditions: If YESTERDAY: Did you combine tobacco and marijuana or cannabis (e.g., blunt, spliff, mixing and smoking in a pipe or bong)? IS Yes
- Question Groups: None

- Response Required: Yes
- Randomize Choice: No Choices
- Seconds to Answer: 0
- Seconds Until Next Button Becomes Active: 0

Breakdown of Each Trigger

Name	Type	Settings
1)	ONCE	Runs from 2024-04-08T11:12:00 UNTIL 2025-04-08T00:00:00.000Z
2)	ONCE	Runs from 2024-09-27T13:26:00 UNTIL 2025-09-27T00:00:00.000Z
3)	ONCE	Runs from 2024-09-27T13:27:00 UNTIL 2025-09-27T00:00:00.000Z
4) Daily Diary	schedule	false
5) Daily Diary	schedule	false
6) Daily Diary - intervention group (training phrase)	schedule	false
7) Daily Diary - intervention group (intervention phrase)	schedule	false
8) Daily Diary - test geofences group	schedule	false

Question & Settings	Question Type	Variable Name	Global Variable Name
22) YESTERDAY: Did you drink any alcohol?	SINGLE_CHOICE	alcoholyest1	G:DD:alcoholyest1

- Display Conditions: None
- Question Groups: None
- Response Required: Yes

- Randomize Choice: No Choices
- Seconds to Answer: 0
- Seconds Until Next Button Becomes Active: 0

Breakdown of Each Trigger

Name	Type	Settings
1)	ONCE	Runs from 2024-04-08T11:12:00 UNTIL 2025-04-08T00:00:00.000Z
2)	ONCE	Runs from 2024-09-27T13:26:00 UNTIL 2025-09-27T00:00:00.000Z
3)	ONCE	Runs from 2024-09-27T13:27:00 UNTIL 2025-09-27T00:00:00.000Z
4) Daily Diary	schedule	false
5) Daily Diary	schedule	false
6) Daily Diary - intervention group (training phrase)	schedule	false
7) Daily Diary - intervention group (intervention phrase)	schedule	false
8) Daily Diary - test geofences group	schedule	false

Question & Settings	Question Type	Variable Name	Global Variable Name
23) YESTERDAY: Number of alcoholic drinks?	SINGLE_CHOICE	alcoholyest2	G:DD:alcoholyest2

- Display Conditions: If YESTERDAY: Did you drink any alcohol? IS Yes
- Question Groups: None
- Response Required: Yes
- Randomize Choice: No Choices

- Seconds to Answer: 0
- Seconds Until Next Button Becomes Active: 0

Breakdown of Each Trigger

Name	Type	Settings
1)	ONCE	Runs from 2024-04-08T11:12:00 UNTIL 2025-04-08T00:00:00.000Z
2)	ONCE	Runs from 2024-09-27T13:26:00 UNTIL 2025-09-27T00:00:00.000Z
3)	ONCE	Runs from 2024-09-27T13:27:00 UNTIL 2025-09-27T00:00:00.000Z
4) Daily Diary	schedule	false
5) Daily Diary	schedule	false
6) Daily Diary - intervention group (training phrase)	schedule	false
7) Daily Diary - intervention group (intervention phrase)	schedule	false
8) Daily Diary - test geofences group	schedule	false

Question & Settings	Question Type	Variable Name	Global Variable Name
24) YESTERDAY: While drinking ALCOHOL, did you also use CIGARETTES?	SINGLE_CHOICE	combineyest3	G:DD:combineyest3

- Display Conditions: (If YESTERDAY: How many cigarettes? IS NOT 0 - I did not smoke yesterday (not even a puff)) AND (If YESTERDAY: Did you drink any alcohol? IS Yes)
- Question Groups: None
- Response Required: Yes

- Randomize Choice: No Choices
- Seconds to Answer: 0
- Seconds Until Next Button Becomes Active: 0

Breakdown of Each Trigger

Name	Type	Settings
1)	ONCE	Runs from 2024-04-08T11:12:00 UNTIL 2025-04-08T00:00:00.000Z
2)	ONCE	Runs from 2024-09-27T13:26:00 UNTIL 2025-09-27T00:00:00.000Z
3)	ONCE	Runs from 2024-09-27T13:27:00 UNTIL 2025-09-27T00:00:00.000Z
4) Daily Diary	schedule	false
5) Daily Diary	schedule	false
6) Daily Diary - intervention group (training phrase)	schedule	false
7) Daily Diary - intervention group (intervention phrase)	schedule	false
8) Daily Diary - test geofences group	schedule	false

Question & Settings	Question Type	Variable Name	Global Variable Name
25) YESTERDAY: While drinking ALCOHOL, did you also use E-CIGS/VAPES with nicotine?	SINGLE_CHOICE	combineyest4	G:DD:combineyest4

- Display Conditions: ((If YESTERDAY: Did you use Juul or other pod e-cigarette? IS Yes) OR (If YESTERDAY: Did you use another type of e-cigarette (e.g., vape pen, tank)? IS Yes)) AND (If YESTERDAY: Did you drink any alcohol? IS Yes)
- Question Groups: None

- Response Required: Yes
- Randomize Choice: No Choices
- Seconds to Answer: 0
- Seconds Until Next Button Becomes Active: 0

Breakdown of Each Trigger

Name	Type	Settings
1)	ONCE	Runs from 2024-04-08T11:12:00 UNTIL 2025-04-08T00:00:00.000Z
2)	ONCE	Runs from 2024-09-27T13:26:00 UNTIL 2025-09-27T00:00:00.000Z
3)	ONCE	Runs from 2024-09-27T13:27:00 UNTIL 2025-09-27T00:00:00.000Z
4) Daily Diary	schedule	false
5) Daily Diary	schedule	false
6) Daily Diary - intervention group (training phrase)	schedule	false
7) Daily Diary - intervention group (intervention phrase)	schedule	false
8) Daily Diary - test geofences group	schedule	false

Question & Settings	Question Type	Variable Name	Global Variable Name
26) YESTERDAY: While using CANNABIS, did you also use CIGARETTES?	SINGLE_CHOICE	combineyest5	G:DD:combineyest5

- Display Conditions: (If YESTERDAY: How many cigarettes? IS NOT 0 - I did not smoke yesterday (not even a puff)) AND (If YESTERDAY: Did you use a marijuana or cannabis product? IS Yes)

- Question Groups: None
- Response Required: Yes
- Randomize Choice: No Choices
- Seconds to Answer: 0
- Seconds Until Next Button Becomes Active: 0

Breakdown of Each Trigger

Name	Type	Settings
1)	ONCE	Runs from 2024-04-08T11:12:00 UNTIL 2025-04-08T00:00:00.000Z
2)	ONCE	Runs from 2024-09-27T13:26:00 UNTIL 2025-09-27T00:00:00.000Z
3)	ONCE	Runs from 2024-09-27T13:27:00 UNTIL 2025-09-27T00:00:00.000Z
4) Daily Diary	schedule	false
5) Daily Diary	schedule	false
6) Daily Diary - intervention group (training phrase)	schedule	false
7) Daily Diary - intervention group (intervention phrase)	schedule	false
8) Daily Diary - test geofences group	schedule	false

Question & Settings	Question Type	Variable Name	Global Variable Name
27) YESTERDAY: While using CANNABIS, did you also use E-CIGS/VAPES with nicotine?	SINGLE_CHOICE	combineyest6	G:DD:combineyest6

- Display Conditions: ((If YESTERDAY: Did you use Juul or other pod e-cigarette? IS Yes) OR (If YESTERDAY: Did you use another type of e-

cigarette (e.g., vape pen, tank)? IS Yes)) AND (If YESTERDAY: Did you use a marijuana or cannabis product? IS Yes)
- Question Groups: None
- Response Required: Yes
- Randomize Choice: No Choices
- Seconds to Answer: 0
- Seconds Until Next Button Becomes Active: 0

Breakdown of Each Trigger

Name	Type	Settings
1)	ONCE	Runs from 2024-04-08T11:12:00 UNTIL 2025-04-08T00:00:00.000Z
2)	ONCE	Runs from 2024-09-27T13:26:00 UNTIL 2025-09-27T00:00:00.000Z
3)	ONCE	Runs from 2024-09-27T13:27:00 UNTIL 2025-09-27T00:00:00.000Z
4) Daily Diary	schedule	false
5) Daily Diary	schedule	false
6) Daily Diary - intervention group (training phrase)	schedule	false
7) Daily Diary - intervention group (intervention phrase)	schedule	false
8) Daily Diary - test geofences group	schedule	false

Question & Settings	Question Type	Variable Name	Global Variable Name
28) YESTERDAY: How many of your CIGARETTES did you smoke while drinking ALCOHOL?	SINGLE_CHOICE	combineyest7	G:DD:combineyest7

- Display Conditions: (If YESTERDAY: While drinking ALCOHOL, did you also use CIGARETTES? IS Yes)
- Question Groups: None
- Response Required: Yes
- Randomize Choice: No Choices
- Seconds to Answer: 0
- Seconds Until Next Button Becomes Active: 0

Breakdown of Each Trigger

Name	Type	Settings
1)	ONCE	Runs from 2024-04-08T11:12:00 UNTIL 2025-04-08T00:00:00.000Z
2)	ONCE	Runs from 2024-09-27T13:26:00 UNTIL 2025-09-27T00:00:00.000Z
3)	ONCE	Runs from 2024-09-27T13:27:00 UNTIL 2025-09-27T00:00:00.000Z
4) Daily Diary	schedule	false
5) Daily Diary	schedule	false
6) Daily Diary - intervention group (training phrase)	schedule	false
7) Daily Diary - intervention group (intervention phrase)	schedule	false
8) Daily Diary - test geofences group	schedule	false

Question & Settings	Question Type	Variable Name	Global Variable Name
29) YESTERDAY: How many of your CIGARETTES did you smoke while using CANNABIS?	SINGLE_CHOICE	combineyest8	G:DD:combineyest8

- Display Conditions: (If YESTERDAY: While using CANNABIS, did you also use CIGARETTES? IS Yes)
- Question Groups: None
- Response Required: Yes
- Randomize Choice: No Choices
- Seconds to Answer: 0
- Seconds Until Next Button Becomes Active: 0

Breakdown of Each Trigger

Name	Type	Settings
1)	ONCE	Runs from 2024-04-08T11:12:00 UNTIL 2025-04-08T00:00:00.000Z
2)	ONCE	Runs from 2024-09-27T13:26:00 UNTIL 2025-09-27T00:00:00.000Z
3)	ONCE	Runs from 2024-09-27T13:27:00 UNTIL 2025-09-27T00:00:00.000Z
4) Daily Diary	schedule	false
5) Daily Diary	schedule	false
6) Daily Diary - intervention group (training phrase)	schedule	false
7) Daily Diary - intervention group (intervention phrase)	schedule	false
8) Daily Diary - test geofences group	schedule	false

Question & Settings	Question Type	Variable Name	Global Variable Name
30) YESTERDAY: How much of your E-CIG/VAPE use with nicotine was while drinking ALCOHOL?	SINGLE_CHOICE	combineyest9	G:DD:combineyest9

- Display Conditions: (If YESTERDAY: While drinking ALCOHOL, did you also use E-CIGS/VAPES with nicotine? IS Yes)
- Question Groups: None
- Response Required: Yes
- Randomize Choice: No Choices
- Seconds to Answer: 0
- Seconds Until Next Button Becomes Active: 0

Breakdown of Each Trigger

Name	Type	Settings
1)	ONCE	Runs from 2024-04-08T11:12:00 UNTIL 2025-04-08T00:00:00.000Z
2)	ONCE	Runs from 2024-09-27T13:26:00 UNTIL 2025-09-27T00:00:00.000Z
3)	ONCE	Runs from 2024-09-27T13:27:00 UNTIL 2025-09-27T00:00:00.000Z
4) Daily Diary	schedule	false
5) Daily Diary	schedule	false
6) Daily Diary - intervention group (training phrase)	schedule	false
7) Daily Diary - intervention group (intervention phrase)	schedule	false
8) Daily Diary - test geofences group	schedule	false

Question & Settings	Question Type	Variable Name	Global Variable Name
31) YESTERDAY: How much of your E-CIG/VAPE use with nicotine was while using	SINGLE_CHOICE	combineyest10	G:DD:combineyest10

CANNABIS?

- Display Conditions: (If YESTERDAY: While using CANNABIS, did you also use E-CIGS/VAPES with nicotine? IS Yes)
- Question Groups: None
- Response Required: Yes
- Randomize Choice: No Choices
- Seconds to Answer: 0
- Seconds Until Next Button Becomes Active: 0

Breakdown of Each Trigger

Name	Type	Settings
1)	ONCE	Runs from 2024-04-08T11:12:00 UNTIL 2025-04-08T00:00:00.000Z
2)	ONCE	Runs from 2024-09-27T13:26:00 UNTIL 2025-09-27T00:00:00.000Z
3)	ONCE	Runs from 2024-09-27T13:27:00 UNTIL 2025-09-27T00:00:00.000Z
4) Daily Diary	schedule	false
5) Daily Diary	schedule	false
6) Daily Diary - intervention group (training phrase)	schedule	false
7) Daily Diary - intervention group (intervention phrase)	schedule	false
8) Daily Diary - test geofences group	schedule	false

Question & Settings	Question Type	Variable Name	Global Variable Name
32) YESTERDAY: I felt	SINGLE_CHOICE	discriminationyest1	G:DD:discriminationyest1

discriminated against.

- Display Conditions: None
- Question Groups: None
- Response Required: Yes
- Randomize Choice: No Choices
- Seconds to Answer: 0
- Seconds Until Next Button Becomes Active: 0

Breakdown of Each Trigger

Name	Type	Settings
1)	ONCE	Runs from 2024-04-08T11:12:00 UNTIL 2025-04-08T00:00:00.000Z
2)	ONCE	Runs from 2024-09-27T13:26:00 UNTIL 2025-09-27T00:00:00.000Z
3)	ONCE	Runs from 2024-09-27T13:27:00 UNTIL 2025-09-27T00:00:00.000Z
4) Daily Diary	schedule	false
5) Daily Diary	schedule	false
6) Daily Diary - intervention group (training phrase)	schedule	false
7) Daily Diary - intervention group (intervention phrase)	schedule	false
8) Daily Diary - test geofences group	schedule	false

Question & Settings	Question Type	Variable Name	Global Variable Name
33) YESTERDAY: What was the	MULTIPLE_CHOICE	discriminationyest2	G:DD:discriminationyest2

main reason(s) for the discrimination that you experienced?

- Display Conditions: If YESTERDAY: I felt discriminated against. IS Yes
- Question Groups: None
- Response Required: Yes
- Randomize Choice: No Choices
- Seconds to Answer: 0
- Seconds Until Next Button Becomes Active: 0

Breakdown of Each Trigger

Name	Type	Settings
1)	ONCE	Runs from 2024-04-08T11:12:00 UNTIL 2025-04-08T00:00:00.000Z
2)	ONCE	Runs from 2024-09-27T13:26:00 UNTIL 2025-09-27T00:00:00.000Z
3)	ONCE	Runs from 2024-09-27T13:27:00 UNTIL 2025-09-27T00:00:00.000Z
4) Daily Diary	schedule	false
5) Daily Diary	schedule	false
6) Daily Diary - intervention group (training phrase)	schedule	false
7) Daily Diary - intervention group (intervention phrase)	schedule	false
8) Daily Diary - test geofences group	schedule	false

Question & Settings	Question Type	Variable Name	Global Variable Name
34) YESTERDAY: Who discriminated against you? Check all that apply	MULTIPLE_CHOICE	discriminationyest3	G:DD:discriminationyest3

- Display Conditions: If YESTERDAY: I felt discriminated against. IS Yes
- Question Groups: None
- Response Required: Yes
- Randomize Choice: No Choices
- Seconds to Answer: 0
- Seconds Until Next Button Becomes Active: 0

Breakdown of Each Trigger

Name	Type	Settings
1)	ONCE	Runs from 2024-04-08T11:12:00 UNTIL 2025-04-08T00:00:00.000Z
2)	ONCE	Runs from 2024-09-27T13:26:00 UNTIL 2025-09-27T00:00:00.000Z
3)	ONCE	Runs from 2024-09-27T13:27:00 UNTIL 2025-09-27T00:00:00.000Z
4) Daily Diary	schedule	false
5) Daily Diary	schedule	false
6) Daily Diary - intervention group (training phrase)	schedule	false
7) Daily Diary - intervention group (intervention phrase)	schedule	false
8) Daily Diary - test geofences group	schedule	false

Survey Information for Location Assessment

- Name: Location Assessment
- Type: MOBILE
- Enable Survey End Screen: No
- Randomized Question Order: No
- Disable Review Question Screen: Yes
- Randomly Trigger a Single Group: No
- Number of Survey Triggers: 50
- Number of Questions: 26

Data Format

Variable Name	Format	Position	Description
Response ID	Character	1	Unique Response Identifier
User ID	Character	2	Unique Participant Identifier
Survey Started Date	Character	3	Date participant started to answer the survey
Survey Started Time	Character	4	Time participant started to answer the survey
Survey Submitted Date	Character	5	Date participant submitted survey
Survey Submitted Time	Character	6	Time participant submitted survey
Time Zone	Character	7	Identifies the time zone in UTC of the paricipant at the time of survey submission

Submission Location	Numeric	8	Location of the user at the time of response submission
Device OS	Character	9	Identifies the device operating system at the time of submission
Device OS Version	Character	10	Identifies the version device operating system at the time of submission
App Version	Numeric	11	Identifies the app version downloaded on the participant's device at the time of submission
Trigger Date	Character	12	Identifies the date that the trigger was prompted to participant
Trigger Time	Character	13	Identifies the time that the trigger was prompted to participant
Trigger Type	Character	14	Identifies the type of trigger that was prompted to participant
Trigger Name	Character	15	Identifies the name of trigger that was prompted to participant
Trigger Index	Numeric	16	Identifies the order of the trigger based on the parent prompt that the trigger was based on

Question Level Response Variables

Question	Variable Name	Format	Position	Question Type	Choices: Coded Value
Overall feeling right now?	gf_feeling	Character	17	SINGLE_CHOICE	- 1 - Very unpleasant : 1<br>- 2 : 2<br>- 3 : 3

					- 4 : 4<br>- 5 - Very pleasant : 5
Overall arousal/energy level right now?	gf_arousal	Character	18	SINGLE_CHOICE	- 1 - Very low : 1<br>- 2 : 2<br>- 3 : 3<br>- 4 : 4<br>- 5 - Very high : 5
Overall stress level right now?	gf_stress	Character	19	SINGLE_CHOICE	- 1 - Very low : 1<br>- 2 : 2<br>- 3 : 3<br>- 4 : 4<br>- 5 - Very high : 5
Overall anxiety level right now?	gf_anxiety	Character	20	SINGLE_CHOICE	- 1 - Very low : 1<br>- 2 : 2<br>- 3 : 3<br>- 4 : 4<br>- 5 - Very high : 5
Craving a cigarette or tobacco product?	gf_craving	Character	21	SINGLE_CHOICE	- 1 - Very low : 1<br>- 2 : 2<br>- 3 : 3<br>- 4 : 4<br>- 5 - Very high : 5
Where are you?	gf_location	Character	22	SINGLE_CHOICE	- Home : 1<br>- Workplace/School : 2<br>- Other's home : 3<br>- Bar : 4<br>- Restaurant : 5<br>- Vehicle : 6<br>- Walking between places : 7<br>- Public transit stop : 8

					- Other location : 9
Which other location?	gf_locationother	Character	23	TEXT	
Location? Inside/outside.	gf_insideoutside	Character	24	SINGLE_CHOICE	- Inside : 1<br>- Outside (patio, entrance, street, etc.) : 2
Smoking cigarettes allowed?	gf_cigsallowed	Character	25	SINGLE_CHOICE	- Forbidden : 1<br>- Discouraged : 2<br>- Allowed : 3
With others?	gf_social1	Character	26	SINGLE_CHOICE	- Yes : 1<br>- No : 0
Who are you with?	gf_social2	Character	27	MULTIPLE_CHOICE	- Friends : 2<br>- Acquaintances : 3<br>- Family members : 4<br>- Coworkers : 5<br>- Romantic partner : 6<br>- Unknown person : 7
With how many people?	gf_social3	Character	28	SINGLE_CHOICE	- 1 : 1<br>- 2-4 : 2<br>- 5-20 : 3<br>- 21+ : 4
With how many people?	gf_social3_dropdown	Character	29	DROPDOWN	-<br>1 : 1<br>- 2 : 2<br>- 3 : 3<br>- 4 : 4<br>- 5 : 5<br>- 6 : 6<br>- 7 : 7<br>- 8 : 8<br>- 9 : 9

					- 10 : 10<br>- 11 : 11<br>- 12 : 12<br>- 13 : 13<br>- 14 : 14<br>- 15 : 15<br>- 16 : 16<br>- 17 : 17<br>- 18 : 18<br>- 19 : 19<br>- 20 : 20<br>- 21+ : 21
Other people smoking cigarettes or using other tobacco products?	gf_social4	Character	30	SINGLE_CHOICE	- Yes : 1<br>- No : 0
Who was smoking cigarettes or using other tobacco products?	gf_social5	Character	31	MULTIPLE_CHOICE	- Friends : 1<br>- Acquaintances : 2<br>- Family members : 3<br>- Coworkers : 4<br>- Romantic partner : 5<br>- Unknown person : 6
How many people were smoking cigarettes or using other tobacco products?	gf_social6	Character	32	SINGLE_CHOICE	- 1 : 1<br>- 2-4 : 2<br>- 5-20 : 3<br>- 21+ : 4
How many people were smoking cigarettes or using other tobacco products?	gf_social6_dropdown	Character	33	DROPDOWN	-<br>1 : 1<br>- 2 : 2<br>- 3 : 3<br>- 4 : 4<br>- 5 : 5<br>- 6 : 6

					- 7 : 7<br>- 8 : 8<br>- 9 : 9<br>- 10 : 10<br>- 11 : 11<br>- 12 : 12<br>- 13 : 13<br>- 14 : 14<br>- 15 : 15<br>- 16 : 16<br>- 17 : 17<br>- 18 : 18<br>- 19 : 19<br>- 20 : 20<br>- 21+ : 21
What were you doing?	gf_activity	Character	34	SINGLE_CHOICE	- Working/Chores : 1<br>- Inactive/leisure : 2<br>- Interacting with others : 3<br>- Eating/drinking : 4<br>- Between activities : 5<br>- Other activities : 6
What other activities?	gf_activityother	Character	35	TEXT	
Consume any of the following in last hour?	gf_consume	Character	36	MULTIPLE_CHOICE	- Food : 1<br>- Caffeinated drink : 2<br>- Non-caffeinated drink : 3<br>- Alcohol : 4<br>- Cigarettes : 5<br>- Other tobacco product : 6<br>- Marijuana or Cannabis : 7<br>- Other substance or drug : 8

Intoxicated and/or drunk?	gf_intoxicated	Character	37	SINGLE_CHOICE	- 1- No!! : 1<br>- 2 : 2<br>- 3 : 3<br>- 4 : 4<br>- 5 - Yes!! : 5
Saw any of the following?	gf_exposed	Character	38	MULTIPLE_CHOICE	- Cigarette or other tobacco product : 1<br>- Lighter/matches : 2<br>- Cigarette or tobacco product pack : 3<br>- Ashtray : 4<br>- Cigarette or tobacco product in the media : 5<br>- Someone smoking or using tobacco product : 6<br>- Other things related to smoking or tobacco products : 7
SINCE THE LAST SURVEY: I felt discriminated against.	gf_discrimination1	Character	39	SINGLE_CHOICE	- Yes : 1<br>- No : 0
SINCE THE LAST SURVEY: What was the main reason(s) for the discrimination that you experienced?	gf_discrimination2	Character	40	MULTIPLE_CHOICE	- Your age : 1<br>- Your gender : 2<br>- Your race : 3<br>- Your ethnicity or nationality : 4<br>- Your religion : 5<br>- Your height or weight : 6<br>- Some other aspect of your appearance : 7<br>- A physical disability : 8<br>- Your sexual orientation : 9<br>- Being a smoker : 10

					- Being poor : 11<br>- Other : 12
SINCE THE LAST SURVEY: Who discriminated against you?	gf_discrimination3	Character	41	MULTIPLE_CHOICE	- Family member : 1<br>- Romantic partner : 2<br>- Stranger : 3<br>- Acquaintance : 4<br>- Friend : 5<br>- Employer : 6<br>- Other : 7
	int		42	FIELD_GROUP	

Breakdown of Each Question

Question & Settings	Question Type	Variable Name	Global Variable Name
1) Overall feeling right now?	SINGLE_CHOICE	gf_feeling	G:LA:gf_feeling

- Display Conditions: None
- Question Groups: None
- Response Required: Yes
- Randomize Choice: No Choices
- Seconds to Answer: 0
- Seconds Until Next Button Becomes Active: 0

Breakdown of Each Trigger

Name	Type	Settings
1)	ONCE	Runs from 2024-04-08T11:49:00 UNTIL 2025-04-08T00:00:00.000Z

2) Geofence example trigger	schedule	false
3) UserId: 6567c7d46b8a38a70237d196 - Geofences	geolocation	false
4) UserId: 6567cad06b8a38a70237d4dd - Geofences	geolocation	false
5) UserId: 6567cbae6b8a38a70237d532 - Geofences	geolocation	false
6) UserId: 6568bdd76b8a38a70238648b - Geofences	geolocation	false
7) UserId: 6584306828f1dc791eae5abb - Geofences	geolocation	false
8) UserId: 65b0080e29fd6a9d3466f2b5 - Geofences	geolocation	false
9) UserId: 65d0bc2f38a1f8159ed8253b - Geofences	geolocation	false
10) UserId: 65d8d947f7c6ed301283576d - Geofences	geolocation	false
11) UserId: 65dacd5217ddbc9fec6fdade - Geofences	geolocation	false
12) UserId: 65dc2026cc4061e42221d67a - Geofences	geolocation	false
13) UserId: 65de57eb405d00d76d2f03ae - Geofences	geolocation	false
14) UserId: 6660a99c4bbb1eceb296728f - Geofences	geolocation	false
15) UserId: 6658a32b70d761aec4591e9a - Geofences	geolocation	false
16) UserId: 6660a3014bbb1eceb29666bd -	geolocation	false

Geofences		
17) JT - UserId: 66914ab785c52222d31cd6d1- Geofences	geolocation	false
18) JW Geofences - 8/13/24	geolocation	false
19) JT Geofences - 8/13/24	geolocation	false
20) JH Geofences - 8/29/24	geolocation	false
21) JW Geofences - 8/30/24	geolocation	false
22) JD iOS Geofences - 8/30/24	geolocation	false
23) JD Android Geofences - 8/30/24	geolocation	false
24) JD Android Geofences - 8/30/24 (enter)	geolocation	false
25) JD iOS Geofences - 8/30/24 (enter)	geolocation	false
26) JH Geofences - 8/29/24 (enter)	geolocation	false
27) JT Geofences - 8/13/24 (enter)	geolocation	false
28) JT Geofences - 09/4/24 (enter)	geolocation	false
29) JW Geofences - 8/30/24 (enter)	geolocation	false
30) JD Android Geofences - 8/30/24 (enter) (Copy)	geolocation	false
31) JT Geofences - 10/11/24 (enter)	geolocation	false
32) JH Geofences - 10/11/24 (enter)	geolocation	false
33) JW Geofences - 10/11/24 (enter)	geolocation	false
34) JH Geofences - 10/16/24 (enter)	geolocation	false
35) JD Android Geofences - 10/16/24 (enter)	geolocation	false
36) JT Geofences - 10/16/24 (enter)	geolocation	false
37) spbm_ftp102 _Geofences	geolocation	false
38) spbm_ftp112_Geofences	geolocation	false

39) spbm_ftp113_Geofences	geolocation	false
40) spbm_ftp121_Geofences	geolocation	false
41) spbm_ftp275_Geofences	geolocation	false
42) SPBM_FTP179_Geofences	geolocation	false
43) SPBM_FTP172_Geofences	geolocation	false
44) SPBM_FTP477_Geofences	geolocation	false
45) SPBM_FTP449_Geofences	geolocation	false
46) SPBM_FTP482_Geofences	geolocation	false
47) SPBM_FTP483_Geofences	geolocation	false
48) SPBM_FTP479_Geofences	geolocation	false
49) SPBM_FTP495_Geofences	geolocation	false
50) SPBM_FTP271_Geofences	geolocation	false

Question & Settings	Question Type	Variable Name	Global Variable Name
2) Overall arousal/energy level right now?	SINGLE_CHOICE	gf_arousal	G:LA:gf_arousal

- Display Conditions: None
- Question Groups: None
- Response Required: Yes
- Randomize Choice: No Choices
- Seconds to Answer: 0
- Seconds Until Next Button Becomes Active: 0

Breakdown of Each Trigger

Name	Type	Settings

1)	ONCE	Runs from 2024-04-08T11:49:00 UNTIL 2025-04-08T00:00:00.000Z
2) Geofence example trigger	schedule	false
3) UserId: 6567c7d46b8a38a70237d196 - Geofences	geolocation	false
4) UserId: 6567cad06b8a38a70237d4dd - Geofences	geolocation	false
5) UserId: 6567cbae6b8a38a70237d532 - Geofences	geolocation	false
6) UserId: 6568bdd76b8a38a70238648b - Geofences	geolocation	false
7) UserId: 6584306828f1dc791eae5abb - Geofences	geolocation	false
8) UserId: 65b0080e29fd6a9d3466f2b5 - Geofences	geolocation	false
9) UserId: 65d0bc2f38a1f8159ed8253b - Geofences	geolocation	false
10) UserId: 65d8d947f7c6ed301283576d - Geofences	geolocation	false
11) UserId: 65dacd5217ddbc9fec6fdade - Geofences	geolocation	false
12) UserId: 65dc2026cc4061e42221d67a - Geofences	geolocation	false
13) UserId: 65de57eb405d00d76d2f03ae - Geofences	geolocation	false
14) UserId: 6660a99c4bbb1eceb296728f - Geofences	geolocation	false
15) UserId: 6658a32b70d761aec4591e9a -	geolocation	false

Geofences		
16) UserId: 6660a3014bbb1eceb29666bd - Geofences	geolocation	false
17) JT - UserId: 66914ab785c52222d31cd6d1- Geofences	geolocation	false
18) JW Geofences - 8/13/24	geolocation	false
19) JT Geofences - 8/13/24	geolocation	false
20) JH Geofences - 8/29/24	geolocation	false
21) JW Geofences - 8/30/24	geolocation	false
22) JD iOS Geofences - 8/30/24	geolocation	false
23) JD Android Geofences - 8/30/24	geolocation	false
24) JD Android Geofences - 8/30/24 (enter)	geolocation	false
25) JD iOS Geofences - 8/30/24 (enter)	geolocation	false
26) JH Geofences - 8/29/24 (enter)	geolocation	false
27) JT Geofences - 8/13/24 (enter)	geolocation	false
28) JT Geofences - 09/4/24 (enter)	geolocation	false
29) JW Geofences - 8/30/24 (enter)	geolocation	false
30) JD Android Geofences - 8/30/24 (enter) (Copy)	geolocation	false
31) JT Geofences - 10/11/24 (enter)	geolocation	false
32) JH Geofences - 10/11/24 (enter)	geolocation	false
33) JW Geofences - 10/11/24 (enter)	geolocation	false
34) JH Geofences - 10/16/24 (enter)	geolocation	false
35) JD Android Geofences - 10/16/24 (enter)	geolocation	false
36) JT Geofences - 10/16/24 (enter)	geolocation	false

37) spbm_ftp102 _Geofences	geolocation	false
38) spbm_ftp112_Geofences	geolocation	false
39) spbm_ftp113_Geofences	geolocation	false
40) spbm_ftp121_Geofences	geolocation	false
41) spbm_ftp275_Geofences	geolocation	false
42) SPBM_FTP179_Geofences	geolocation	false
43) SPBM_FTP172_Geofences	geolocation	false
44) SPBM_FTP477_Geofences	geolocation	false
45) SPBM_FTP449_Geofences	geolocation	false
46) SPBM_FTP482_Geofences	geolocation	false
47) SPBM_FTP483_Geofences	geolocation	false
48) SPBM_FTP479_Geofences	geolocation	false
49) SPBM_FTP495_Geofences	geolocation	false
50) SPBM_FTP271_Geofences	geolocation	false

Question & Settings	Question Type	Variable Name	Global Variable Name
3) Overall stress level right now?	SINGLE_CHOICE	gf_stress	G:LA:gf_stress

- Display Conditions: None
- Question Groups: None
- Response Required: Yes
- Randomize Choice: No Choices
- Seconds to Answer: 0
- Seconds Until Next Button Becomes Active: 0

Breakdown of Each Trigger

Name	Type	Settings
1)	ONCE	Runs from 2024-04-08T11:49:00 UNTIL 2025-04-08T00:00:00.000Z
2) Geofence example trigger	schedule	false
3) UserId: 6567c7d46b8a38a70237d196 - Geofences	geolocation	false
4) UserId: 6567cad06b8a38a70237d4dd - Geofences	geolocation	false
5) UserId: 6567cbae6b8a38a70237d532 - Geofences	geolocation	false
6) UserId: 6568bdd76b8a38a70238648b - Geofences	geolocation	false
7) UserId: 6584306828f1dc791eae5abb - Geofences	geolocation	false
8) UserId: 65b0080e29fd6a9d3466f2b5 - Geofences	geolocation	false
9) UserId: 65d0bc2f38a1f8159ed8253b - Geofences	geolocation	false
10) UserId: 65d8d947f7c6ed301283576d - Geofences	geolocation	false
11) UserId: 65dacd5217ddbc9fec6fdade - Geofences	geolocation	false
12) UserId: 65dc2026cc4061e42221d67a - Geofences	geolocation	false
13) UserId: 65de57eb405d00d76d2f03ae - Geofences	geolocation	false
14) UserId: 6660a99c4bbb1eceb296728f - Geofences	geolocation	false

15) UserId: 6658a32b70d761aec4591e9a - Geofences	geolocation	false
16) UserId: 6660a3014bbb1eceb29666bd - Geofences	geolocation	false
17) JT - UserId: 66914ab785c52222d31cd6d1- Geofences	geolocation	false
18) JW Geofences - 8/13/24	geolocation	false
19) JT Geofences - 8/13/24	geolocation	false
20) JH Geofences - 8/29/24	geolocation	false
21) JW Geofences - 8/30/24	geolocation	false
22) JD iOS Geofences - 8/30/24	geolocation	false
23) JD Android Geofences - 8/30/24	geolocation	false
24) JD Android Geofences - 8/30/24 (enter)	geolocation	false
25) JD iOS Geofences - 8/30/24 (enter)	geolocation	false
26) JH Geofences - 8/29/24 (enter)	geolocation	false
27) JT Geofences - 8/13/24 (enter)	geolocation	false
28) JT Geofences - 09/4/24 (enter)	geolocation	false
29) JW Geofences - 8/30/24 (enter)	geolocation	false
30) JD Android Geofences - 8/30/24 (enter) (Copy)	geolocation	false
31) JT Geofences - 10/11/24 (enter)	geolocation	false
32) JH Geofences - 10/11/24 (enter)	geolocation	false
33) JW Geofences - 10/11/24 (enter)	geolocation	false
34) JH Geofences - 10/16/24 (enter)	geolocation	false
35) JD Android Geofences - 10/16/24 (enter)	geolocation	false

36) JT Geofences - 10/16/24 (enter)	geolocation	false
37) spbm_ftp102 _Geofences	geolocation	false
38) spbm_ftp112_Geofences	geolocation	false
39) spbm_ftp113_Geofences	geolocation	false
40) spbm_ftp121_Geofences	geolocation	false
41) spbm_ftp275_Geofences	geolocation	false
42) SPBM_FTP179_Geofences	geolocation	false
43) SPBM_FTP172_Geofences	geolocation	false
44) SPBM_FTP477_Geofences	geolocation	false
45) SPBM_FTP449_Geofences	geolocation	false
46) SPBM_FTP482_Geofences	geolocation	false
47) SPBM_FTP483_Geofences	geolocation	false
48) SPBM_FTP479_Geofences	geolocation	false
49) SPBM_FTP495_Geofences	geolocation	false
50) SPBM_FTP271_Geofences	geolocation	false

Question & Settings	Question Type	Variable Name	Global Variable Name
4) Overall anxiety level right now?	SINGLE_CHOICE	gf_anxiety	G:LA:gf_anxiety

- Display Conditions: None
- Question Groups: None
- Response Required: Yes
- Randomize Choice: No Choices
- Seconds to Answer: 0
- Seconds Until Next Button Becomes Active: 0

Breakdown of Each Trigger

Name	Type	Settings
1)	ONCE	Runs from 2024-04-08T11:49:00 UNTIL 2025-04-08T00:00:00.000Z
2) Geofence example trigger	schedule	false
3) UserId: 6567c7d46b8a38a70237d196 - Geofences	geolocation	false
4) UserId: 6567cad06b8a38a70237d4dd - Geofences	geolocation	false
5) UserId: 6567cbae6b8a38a70237d532 - Geofences	geolocation	false
6) UserId: 6568bdd76b8a38a70238648b - Geofences	geolocation	false
7) UserId: 6584306828f1dc791eae5abb - Geofences	geolocation	false
8) UserId: 65b0080e29fd6a9d3466f2b5 - Geofences	geolocation	false
9) UserId: 65d0bc2f38a1f8159ed8253b - Geofences	geolocation	false
10) UserId: 65d8d947f7c6ed301283576d - Geofences	geolocation	false
11) UserId: 65dacd5217ddbc9fec6fdade - Geofences	geolocation	false
12) UserId: 65dc2026cc4061e42221d67a - Geofences	geolocation	false
13) UserId: 65de57eb405d00d76d2f03ae - Geofences	geolocation	false
14) UserId: 6660a99c4bbb1eceb296728f -	geolocation	false

Geofences		
15) UserId: 6658a32b70d761aec4591e9a - Geofences	geolocation	false
16) UserId: 6660a3014bbb1eceb29666bd - Geofences	geolocation	false
17) JT - UserId: 66914ab785c52222d31cd6d1- Geofences	geolocation	false
18) JW Geofences - 8/13/24	geolocation	false
19) JT Geofences - 8/13/24	geolocation	false
20) JH Geofences - 8/29/24	geolocation	false
21) JW Geofences - 8/30/24	geolocation	false
22) JD iOS Geofences - 8/30/24	geolocation	false
23) JD Android Geofences - 8/30/24	geolocation	false
24) JD Android Geofences - 8/30/24 (enter)	geolocation	false
25) JD iOS Geofences - 8/30/24 (enter)	geolocation	false
26) JH Geofences - 8/29/24 (enter)	geolocation	false
27) JT Geofences - 8/13/24 (enter)	geolocation	false
28) JT Geofences - 09/4/24 (enter)	geolocation	false
29) JW Geofences - 8/30/24 (enter)	geolocation	false
30) JD Android Geofences - 8/30/24 (enter) (Copy)	geolocation	false
31) JT Geofences - 10/11/24 (enter)	geolocation	false
32) JH Geofences - 10/11/24 (enter)	geolocation	false
33) JW Geofences - 10/11/24 (enter)	geolocation	false
34) JH Geofences - 10/16/24 (enter)	geolocation	false

35) JD Android Geofences - 10/16/24 (enter)	geolocation	false
36) JT Geofences - 10/16/24 (enter)	geolocation	false
37) spbm_ftp102 _Geofences	geolocation	false
38) spbm_ftp112_Geofences	geolocation	false
39) spbm_ftp113_Geofences	geolocation	false
40) spbm_ftp121_Geofences	geolocation	false
41) spbm_ftp275_Geofences	geolocation	false
42) SPBM_FTP179_Geofences	geolocation	false
43) SPBM_FTP172_Geofences	geolocation	false
44) SPBM_FTP477_Geofences	geolocation	false
45) SPBM_FTP449_Geofences	geolocation	false
46) SPBM_FTP482_Geofences	geolocation	false
47) SPBM_FTP483_Geofences	geolocation	false
48) SPBM_FTP479_Geofences	geolocation	false
49) SPBM_FTP495_Geofences	geolocation	false
50) SPBM_FTP271_Geofences	geolocation	false

Question & Settings	Question Type	Variable Name	Global Variable Name
5) Craving a cigarette or tobacco product?	SINGLE_CHOICE	gf_craving	G:LA:gf_craving

- Display Conditions: None
- Question Groups: None
- Response Required: Yes
- Randomize Choice: No Choices
- Seconds to Answer: 0
- Seconds Until Next Button Becomes Active: 0

Breakdown of Each Trigger

Name	Type	Settings
1)	ONCE	Runs from 2024-04-08T11:49:00 UNTIL 2025-04-08T00:00:00.000Z
2) Geofence example trigger	schedule	false
3) UserId: 6567c7d46b8a38a70237d196 - Geofences	geolocation	false
4) UserId: 6567cad06b8a38a70237d4dd - Geofences	geolocation	false
5) UserId: 6567cbae6b8a38a70237d532 - Geofences	geolocation	false
6) UserId: 6568bdd76b8a38a70238648b - Geofences	geolocation	false
7) UserId: 6584306828f1dc791eae5abb - Geofences	geolocation	false
8) UserId: 65b0080e29fd6a9d3466f2b5 - Geofences	geolocation	false
9) UserId: 65d0bc2f38a1f8159ed8253b - Geofences	geolocation	false
10) UserId: 65d8d947f7c6ed301283576d - Geofences	geolocation	false
11) UserId: 65dacd5217ddbc9fec6fdade - Geofences	geolocation	false
12) UserId: 65dc2026cc4061e42221d67a - Geofences	geolocation	false

13) UserId: 65de57eb405d00d76d2f03ae - Geofences	geolocation	false
14) UserId: 6660a99c4bbb1eceb296728f - Geofences	geolocation	false
15) UserId: 6658a32b70d761aec4591e9a - Geofences	geolocation	false
16) UserId: 6660a3014bbb1eceb29666bd - Geofences	geolocation	false
17) JT - UserId: 66914ab785c52222d31cd6d1- Geofences	geolocation	false
18) JW Geofences - 8/13/24	geolocation	false
19) JT Geofences - 8/13/24	geolocation	false
20) JH Geofences - 8/29/24	geolocation	false
21) JW Geofences - 8/30/24	geolocation	false
22) JD iOS Geofences - 8/30/24	geolocation	false
23) JD Android Geofences - 8/30/24	geolocation	false
24) JD Android Geofences - 8/30/24 (enter)	geolocation	false
25) JD iOS Geofences - 8/30/24 (enter)	geolocation	false
26) JH Geofences - 8/29/24 (enter)	geolocation	false
27) JT Geofences - 8/13/24 (enter)	geolocation	false
28) JT Geofences - 09/4/24 (enter)	geolocation	false
29) JW Geofences - 8/30/24 (enter)	geolocation	false
30) JD Android Geofences - 8/30/24 (enter) (Copy)	geolocation	false
31) JT Geofences - 10/11/24 (enter)	geolocation	false
32) JH Geofences - 10/11/24 (enter)	geolocation	false

33) JW Geofences - 10/11/24 (enter)	geolocation	false
34) JH Geofences - 10/16/24 (enter)	geolocation	false
35) JD Android Geofences - 10/16/24 (enter)	geolocation	false
36) JT Geofences - 10/16/24 (enter)	geolocation	false
37) spbm_ftp102 _Geofences	geolocation	false
38) spbm_ftp112_Geofences	geolocation	false
39) spbm_ftp113_Geofences	geolocation	false
40) spbm_ftp121_Geofences	geolocation	false
41) spbm_ftp275_Geofences	geolocation	false
42) SPBM_FTP179_Geofences	geolocation	false
43) SPBM_FTP172_Geofences	geolocation	false
44) SPBM_FTP477_Geofences	geolocation	false
45) SPBM_FTP449_Geofences	geolocation	false
46) SPBM_FTP482_Geofences	geolocation	false
47) SPBM_FTP483_Geofences	geolocation	false
48) SPBM_FTP479_Geofences	geolocation	false
49) SPBM_FTP495_Geofences	geolocation	false
50) SPBM_FTP271_Geofences	geolocation	false

Question & Settings	Question Type	Variable Name	Global Variable Name
6) Where are you?	SINGLE_CHOICE	gf_location	G:LA:gf_location

- Display Conditions: None
- Question Groups: None
- Response Required: Yes
- Randomize Choice: No Choices

- Seconds to Answer: 0
- Seconds Until Next Button Becomes Active: 0

Breakdown of Each Trigger

Name	Type	Settings
1)	ONCE	Runs from 2024-04-08T11:49:00 UNTIL 2025-04-08T00:00:00.000Z
2) Geofence example trigger	schedule	false
3) UserId: 6567c7d46b8a38a70237d196 - Geofences	geolocation	false
4) UserId: 6567cad06b8a38a70237d4dd - Geofences	geolocation	false
5) UserId: 6567cbae6b8a38a70237d532 - Geofences	geolocation	false
6) UserId: 6568bdd76b8a38a70238648b - Geofences	geolocation	false
7) UserId: 6584306828f1dc791eae5abb - Geofences	geolocation	false
8) UserId: 65b0080e29fd6a9d3466f2b5 - Geofences	geolocation	false
9) UserId: 65d0bc2f38a1f8159ed8253b - Geofences	geolocation	false
10) UserId: 65d8d947f7c6ed301283576d - Geofences	geolocation	false
11) UserId: 65dacd5217ddbc9fec6fdade - Geofences	geolocation	false
12) UserId: 65dc2026cc4061e42221d67a -	geolocation	false

Geofences		
13) UserId: 65de57eb405d00d76d2f03ae - Geofences	geolocation	false
14) UserId: 6660a99c4bbb1eceb296728f - Geofences	geolocation	false
15) UserId: 6658a32b70d761aec4591e9a - Geofences	geolocation	false
16) UserId: 6660a3014bbb1eceb29666bd - Geofences	geolocation	false
17) JT - UserId: 66914ab785c52222d31cd6d1- Geofences	geolocation	false
18) JW Geofences - 8/13/24	geolocation	false
19) JT Geofences - 8/13/24	geolocation	false
20) JH Geofences - 8/29/24	geolocation	false
21) JW Geofences - 8/30/24	geolocation	false
22) JD iOS Geofences - 8/30/24	geolocation	false
23) JD Android Geofences - 8/30/24	geolocation	false
24) JD Android Geofences - 8/30/24 (enter)	geolocation	false
25) JD iOS Geofences - 8/30/24 (enter)	geolocation	false
26) JH Geofences - 8/29/24 (enter)	geolocation	false
27) JT Geofences - 8/13/24 (enter)	geolocation	false
28) JT Geofences - 09/4/24 (enter)	geolocation	false
29) JW Geofences - 8/30/24 (enter)	geolocation	false
30) JD Android Geofences - 8/30/24 (enter) (Copy)	geolocation	false
31) JT Geofences - 10/11/24 (enter)	geolocation	false

32) JH Geofences - 10/11/24 (enter)	geolocation	false
33) JW Geofences - 10/11/24 (enter)	geolocation	false
34) JH Geofences - 10/16/24 (enter)	geolocation	false
35) JD Android Geofences - 10/16/24 (enter)	geolocation	false
36) JT Geofences - 10/16/24 (enter)	geolocation	false
37) spbm_ftp102 _Geofences	geolocation	false
38) spbm_ftp112_Geofences	geolocation	false
39) spbm_ftp113_Geofences	geolocation	false
40) spbm_ftp121_Geofences	geolocation	false
41) spbm_ftp275_Geofences	geolocation	false
42) SPBM_FTP179_Geofences	geolocation	false
43) SPBM_FTP172_Geofences	geolocation	false
44) SPBM_FTP477_Geofences	geolocation	false
45) SPBM_FTP449_Geofences	geolocation	false
46) SPBM_FTP482_Geofences	geolocation	false
47) SPBM_FTP483_Geofences	geolocation	false
48) SPBM_FTP479_Geofences	geolocation	false
49) SPBM_FTP495_Geofences	geolocation	false
50) SPBM_FTP271_Geofences	geolocation	false

Question & Settings	Question Type	Variable Name	Global Variable Name
7) Which other location?	TEXT	gf_locationother	G:LA:gf_locationother

- Display Conditions: If Where are you? IS Other location
- Question Groups: None
- Response Required: Yes

- Randomize Choice: No Choices
- Seconds to Answer: 0
- Seconds Until Next Button Becomes Active: 0

Breakdown of Each Trigger

Name	Type	Settings
1)	ONCE	Runs from 2024-04-08T11:49:00 UNTIL 2025-04-08T00:00:00.000Z
2) Geofence example trigger	schedule	false
3) UserId: 6567c7d46b8a38a70237d196 - Geofences	geolocation	false
4) UserId: 6567cad06b8a38a70237d4dd - Geofences	geolocation	false
5) UserId: 6567cbae6b8a38a70237d532 - Geofences	geolocation	false
6) UserId: 6568bdd76b8a38a70238648b - Geofences	geolocation	false
7) UserId: 6584306828f1dc791eae5abb - Geofences	geolocation	false
8) UserId: 65b0080e29fd6a9d3466f2b5 - Geofences	geolocation	false
9) UserId: 65d0bc2f38a1f8159ed8253b - Geofences	geolocation	false
10) UserId: 65d8d947f7c6ed301283576d - Geofences	geolocation	false
11) UserId: 65dacd5217ddbc9fec6fdade - Geofences	geolocation	false

12) UserId: 65dc2026cc4061e42221d67a - Geofences	geolocation	false
13) UserId: 65de57eb405d00d76d2f03ae - Geofences	geolocation	false
14) UserId: 6660a99c4bbb1eceb296728f - Geofences	geolocation	false
15) UserId: 6658a32b70d761aec4591e9a - Geofences	geolocation	false
16) UserId: 6660a3014bbb1eceb29666bd - Geofences	geolocation	false
17) JT - UserId: 66914ab785c52222d31cd6d1- Geofences	geolocation	false
18) JW Geofences - 8/13/24	geolocation	false
19) JT Geofences - 8/13/24	geolocation	false
20) JH Geofences - 8/29/24	geolocation	false
21) JW Geofences - 8/30/24	geolocation	false
22) JD iOS Geofences - 8/30/24	geolocation	false
23) JD Android Geofences - 8/30/24	geolocation	false
24) JD Android Geofences - 8/30/24 (enter)	geolocation	false
25) JD iOS Geofences - 8/30/24 (enter)	geolocation	false
26) JH Geofences - 8/29/24 (enter)	geolocation	false
27) JT Geofences - 8/13/24 (enter)	geolocation	false
28) JT Geofences - 09/4/24 (enter)	geolocation	false
29) JW Geofences - 8/30/24 (enter)	geolocation	false
30) JD Android Geofences - 8/30/24 (enter) (Copy)	geolocation	false

31) JT Geofences - 10/11/24 (enter)	geolocation	false
32) JH Geofences - 10/11/24 (enter)	geolocation	false
33) JW Geofences - 10/11/24 (enter)	geolocation	false
34) JH Geofences - 10/16/24 (enter)	geolocation	false
35) JD Android Geofences - 10/16/24 (enter)	geolocation	false
36) JT Geofences - 10/16/24 (enter)	geolocation	false
37) spbm_ftp102 _Geofences	geolocation	false
38) spbm_ftp112_Geofences	geolocation	false
39) spbm_ftp113_Geofences	geolocation	false
40) spbm_ftp121_Geofences	geolocation	false
41) spbm_ftp275_Geofences	geolocation	false
42) SPBM_FTP179_Geofences	geolocation	false
43) SPBM_FTP172_Geofences	geolocation	false
44) SPBM_FTP477_Geofences	geolocation	false
45) SPBM_FTP449_Geofences	geolocation	false
46) SPBM_FTP482_Geofences	geolocation	false
47) SPBM_FTP483_Geofences	geolocation	false
48) SPBM_FTP479_Geofences	geolocation	false
49) SPBM_FTP495_Geofences	geolocation	false
50) SPBM_FTP271_Geofences	geolocation	false

Question & Settings	Question Type	Variable Name	Global Variable Name
8) Location? Inside/outside.	SINGLE_CHOICE	gf_insideoutside	G:LA:gf_insideoutside

- Display Conditions: None

- Question Groups: None
- Response Required: Yes
- Randomize Choice: No Choices
- Seconds to Answer: 0
- Seconds Until Next Button Becomes Active: 0

Breakdown of Each Trigger

Name	Type	Settings
1)	ONCE	Runs from 2024-04-08T11:49:00 UNTIL 2025-04-08T00:00:00.000Z
2) Geofence example trigger	schedule	false
3) UserId: 6567c7d46b8a38a70237d196 - Geofences	geolocation	false
4) UserId: 6567cad06b8a38a70237d4dd - Geofences	geolocation	false
5) UserId: 6567cbae6b8a38a70237d532 - Geofences	geolocation	false
6) UserId: 6568bdd76b8a38a70238648b - Geofences	geolocation	false
7) UserId: 6584306828f1dc791eae5abb - Geofences	geolocation	false
8) UserId: 65b0080e29fd6a9d3466f2b5 - Geofences	geolocation	false
9) UserId: 65d0bc2f38a1f8159ed8253b - Geofences	geolocation	false
10) UserId: 65d8d947f7c6ed301283576d - Geofences	geolocation	false

11) UserId: 65dacd5217ddbc9fec6fdade - Geofences	geolocation	false
12) UserId: 65dc2026cc4061e42221d67a - Geofences	geolocation	false
13) UserId: 65de57eb405d00d76d2f03ae - Geofences	geolocation	false
14) UserId: 6660a99c4bbb1eceb296728f - Geofences	geolocation	false
15) UserId: 6658a32b70d761aec4591e9a - Geofences	geolocation	false
16) UserId: 6660a3014bbb1eceb29666bd - Geofences	geolocation	false
17) JT - UserId: 66914ab785c52222d31cd6d1- Geofences	geolocation	false
18) JW Geofences - 8/13/24	geolocation	false
19) JT Geofences - 8/13/24	geolocation	false
20) JH Geofences - 8/29/24	geolocation	false
21) JW Geofences - 8/30/24	geolocation	false
22) JD iOS Geofences - 8/30/24	geolocation	false
23) JD Android Geofences - 8/30/24	geolocation	false
24) JD Android Geofences - 8/30/24 (enter)	geolocation	false
25) JD iOS Geofences - 8/30/24 (enter)	geolocation	false
26) JH Geofences - 8/29/24 (enter)	geolocation	false
27) JT Geofences - 8/13/24 (enter)	geolocation	false
28) JT Geofences - 09/4/24 (enter)	geolocation	false
29) JW Geofences - 8/30/24 (enter)	geolocation	false

30) JD Android Geofences - 8/30/24 (enter) (Copy)	geolocation	false	
31) JT Geofences - 10/11/24 (enter)	geolocation	false	
32) JH Geofences - 10/11/24 (enter)	geolocation	false	
33) JW Geofences - 10/11/24 (enter)	geolocation	false	
34) JH Geofences - 10/16/24 (enter)	geolocation	false	
35) JD Android Geofences - 10/16/24 (enter)	geolocation	false	
36) JT Geofences - 10/16/24 (enter)	geolocation	false	
37) spbm_ftp102 _Geofences	geolocation	false	
38) spbm_ftp112_Geofences	geolocation	false	
39) spbm_ftp113_Geofences	geolocation	false	
40) spbm_ftp121_Geofences	geolocation	false	
41) spbm_ftp275_Geofences	geolocation	false	
42) SPBM_FTP179_Geofences	geolocation	false	
43) SPBM_FTP172_Geofences	geolocation	false	
44) SPBM_FTP477_Geofences	geolocation	false	
45) SPBM_FTP449_Geofences	geolocation	false	
46) SPBM_FTP482_Geofences	geolocation	false	
47) SPBM_FTP483_Geofences	geolocation	false	
48) SPBM_FTP479_Geofences	geolocation	false	
49) SPBM_FTP495_Geofences	geolocation	false	
50) SPBM_FTP271_Geofences	geolocation	false	

Question & Settings	Question Type	Variable Name	Global Variable Name
9) Smoking cigarettes allowed?	SINGLE_CHOICE	gf_cigsallowed	G:LA:gf_cigsallowed

- Display Conditions: None
- Question Groups: None
- Response Required: Yes
- Randomize Choice: No Choices
- Seconds to Answer: 0
- Seconds Until Next Button Becomes Active: 0

Breakdown of Each Trigger

Name	Type	Settings
1)	ONCE	Runs from 2024-04-08T11:49:00 UNTIL 2025-04-08T00:00:00.000Z
2) Geofence example trigger	schedule	false
3) UserId: 6567c7d46b8a38a70237d196 - Geofences	geolocation	false
4) UserId: 6567cad06b8a38a70237d4dd - Geofences	geolocation	false
5) UserId: 6567cbae6b8a38a70237d532 - Geofences	geolocation	false
6) UserId: 6568bdd76b8a38a70238648b - Geofences	geolocation	false
7) UserId: 6584306828f1dc791eae5abb - Geofences	geolocation	false
8) UserId: 65b0080e29fd6a9d3466f2b5 - Geofences	geolocation	false
9) UserId: 65d0bc2f38a1f8159ed8253b - Geofences	geolocation	false

10) UserId: 65d8d947f7c6ed301283576d - Geofences	geolocation	false
11) UserId: 65dacd5217ddbc9fec6fdade - Geofences	geolocation	false
12) UserId: 65dc2026cc4061e42221d67a - Geofences	geolocation	false
13) UserId: 65de57eb405d00d76d2f03ae - Geofences	geolocation	false
14) UserId: 6660a99c4bbb1eceb296728f - Geofences	geolocation	false
15) UserId: 6658a32b70d761aec4591e9a - Geofences	geolocation	false
16) UserId: 6660a3014bbb1eceb29666bd - Geofences	geolocation	false
17) JT - UserId: 66914ab785c52222d31cd6d1- Geofences	geolocation	false
18) JW Geofences - 8/13/24	geolocation	false
19) JT Geofences - 8/13/24	geolocation	false
20) JH Geofences - 8/29/24	geolocation	false
21) JW Geofences - 8/30/24	geolocation	false
22) JD iOS Geofences - 8/30/24	geolocation	false
23) JD Android Geofences - 8/30/24	geolocation	false
24) JD Android Geofences - 8/30/24 (enter)	geolocation	false
25) JD iOS Geofences - 8/30/24 (enter)	geolocation	false
26) JH Geofences - 8/29/24 (enter)	geolocation	false
27) JT Geofences - 8/13/24 (enter)	geolocation	false

28) JT Geofences - 09/4/24 (enter)	geolocation	false
29) JW Geofences - 8/30/24 (enter)	geolocation	false
30) JD Android Geofences - 8/30/24 (enter) (Copy)	geolocation	false
31) JT Geofences - 10/11/24 (enter)	geolocation	false
32) JH Geofences - 10/11/24 (enter)	geolocation	false
33) JW Geofences - 10/11/24 (enter)	geolocation	false
34) JH Geofences - 10/16/24 (enter)	geolocation	false
35) JD Android Geofences - 10/16/24 (enter)	geolocation	false
36) JT Geofences - 10/16/24 (enter)	geolocation	false
37) spbm_ftp102 _Geofences	geolocation	false
38) spbm_ftp112_Geofences	geolocation	false
39) spbm_ftp113_Geofences	geolocation	false
40) spbm_ftp121_Geofences	geolocation	false
41) spbm_ftp275_Geofences	geolocation	false
42) SPBM_FTP179_Geofences	geolocation	false
43) SPBM_FTP172_Geofences	geolocation	false
44) SPBM_FTP477_Geofences	geolocation	false
45) SPBM_FTP449_Geofences	geolocation	false
46) SPBM_FTP482_Geofences	geolocation	false
47) SPBM_FTP483_Geofences	geolocation	false
48) SPBM_FTP479_Geofences	geolocation	false
49) SPBM_FTP495_Geofences	geolocation	false
50) SPBM_FTP271_Geofences	geolocation	false

Question & Settings	Question Type	Variable Name	Global Variable Name
10) With others?	SINGLE_CHOICE	gf_social1	G:LA:gf_social1

- Display Conditions: None
- Question Groups: None
- Response Required: Yes
- Randomize Choice: No Choices
- Seconds to Answer: 0
- Seconds Until Next Button Becomes Active: 0

Breakdown of Each Trigger

Name	Type	Settings
1)	ONCE	Runs from 2024-04-08T11:49:00 UNTIL 2025-04-08T00:00:00.000Z
2) Geofence example trigger	schedule	false
3) UserId: 6567c7d46b8a38a70237d196 - Geofences	geolocation	false
4) UserId: 6567cad06b8a38a70237d4dd - Geofences	geolocation	false
5) UserId: 6567cbae6b8a38a70237d532 - Geofences	geolocation	false
6) UserId: 6568bdd76b8a38a70238648b - Geofences	geolocation	false
7) UserId: 6584306828f1dc791eae5abb - Geofences	geolocation	false
8) UserId: 65b0080e29fd6a9d3466f2b5 - Geofences	geolocation	false

9) UserId: 65d0bc2f38a1f8159ed8253b - Geofences	geolocation	false
10) UserId: 65d8d947f7c6ed301283576d - Geofences	geolocation	false
11) UserId: 65dacd5217ddbc9fec6fdade - Geofences	geolocation	false
12) UserId: 65dc2026cc4061e42221d67a - Geofences	geolocation	false
13) UserId: 65de57eb405d00d76d2f03ae - Geofences	geolocation	false
14) UserId: 6660a99c4bbb1eceb296728f - Geofences	geolocation	false
15) UserId: 6658a32b70d761aec4591e9a - Geofences	geolocation	false
16) UserId: 6660a3014bbb1eceb29666bd - Geofences	geolocation	false
17) JT - UserId: 66914ab785c52222d31cd6d1- Geofences	geolocation	false
18) JW Geofences - 8/13/24	geolocation	false
19) JT Geofences - 8/13/24	geolocation	false
20) JH Geofences - 8/29/24	geolocation	false
21) JW Geofences - 8/30/24	geolocation	false
22) JD iOS Geofences - 8/30/24	geolocation	false
23) JD Android Geofences - 8/30/24	geolocation	false
24) JD Android Geofences - 8/30/24 (enter)	geolocation	false
25) JD iOS Geofences - 8/30/24 (enter)	geolocation	false
26) JH Geofences - 8/29/24 (enter)	geolocation	false

27) JT Geofences - 8/13/24 (enter)	geolocation	false
28) JT Geofences - 09/4/24 (enter)	geolocation	false
29) JW Geofences - 8/30/24 (enter)	geolocation	false
30) JD Android Geofences - 8/30/24 (enter) (Copy)	geolocation	false
31) JT Geofences - 10/11/24 (enter)	geolocation	false
32) JH Geofences - 10/11/24 (enter)	geolocation	false
33) JW Geofences - 10/11/24 (enter)	geolocation	false
34) JH Geofences - 10/16/24 (enter)	geolocation	false
35) JD Android Geofences - 10/16/24 (enter)	geolocation	false
36) JT Geofences - 10/16/24 (enter)	geolocation	false
37) spbm_ftp102 _Geofences	geolocation	false
38) spbm_ftp112_Geofences	geolocation	false
39) spbm_ftp113_Geofences	geolocation	false
40) spbm_ftp121_Geofences	geolocation	false
41) spbm_ftp275_Geofences	geolocation	false
42) SPBM_FTP179_Geofences	geolocation	false
43) SPBM_FTP172_Geofences	geolocation	false
44) SPBM_FTP477_Geofences	geolocation	false
45) SPBM_FTP449_Geofences	geolocation	false
46) SPBM_FTP482_Geofences	geolocation	false
47) SPBM_FTP483_Geofences	geolocation	false
48) SPBM_FTP479_Geofences	geolocation	false
49) SPBM_FTP495_Geofences	geolocation	false
50) SPBM_FTP271_Geofences	geolocation	false

Question & Settings	Question Type	Variable Name	Global Variable Name
11) Who are you with?	MULTIPLE_CHOICE	gf_social2	G:LA:gf_social2

- Display Conditions: If With others? IS Yes
- Question Groups: None
- Response Required: Yes
- Randomize Choice: No Choices
- Seconds to Answer: 0
- Seconds Until Next Button Becomes Active: 0

Breakdown of Each Trigger

Name	Type	Settings
1)	ONCE	Runs from 2024-04-08T11:49:00 UNTIL 2025-04-08T00:00:00.000Z
2) Geofence example trigger	schedule	false
3) UserId: 6567c7d46b8a38a70237d196 - Geofences	geolocation	false
4) UserId: 6567cad06b8a38a70237d4dd - Geofences	geolocation	false
5) UserId: 6567cbae6b8a38a70237d532 - Geofences	geolocation	false
6) UserId: 6568bdd76b8a38a70238648b - Geofences	geolocation	false
7) UserId: 6584306828f1dc791eae5abb - Geofences	geolocation	false
8) UserId: 65b0080e29fd6a9d3466f2b5 -	geolocation	false

Geofences		
9) UserId: 65d0bc2f38a1f8159ed8253b - Geofences	geolocation	false
10) UserId: 65d8d947f7c6ed301283576d - Geofences	geolocation	false
11) UserId: 65dacd5217ddbc9fec6fdade - Geofences	geolocation	false
12) UserId: 65dc2026cc4061e42221d67a - Geofences	geolocation	false
13) UserId: 65de57eb405d00d76d2f03ae - Geofences	geolocation	false
14) UserId: 6660a99c4bbb1eceb296728f - Geofences	geolocation	false
15) UserId: 6658a32b70d761aec4591e9a - Geofences	geolocation	false
16) UserId: 6660a3014bbb1eceb29666bd - Geofences	geolocation	false
17) JT - UserId: 66914ab785c52222d31cd6d1- Geofences	geolocation	false
18) JW Geofences - 8/13/24	geolocation	false
19) JT Geofences - 8/13/24	geolocation	false
20) JH Geofences - 8/29/24	geolocation	false
21) JW Geofences - 8/30/24	geolocation	false
22) JD iOS Geofences - 8/30/24	geolocation	false
23) JD Android Geofences - 8/30/24	geolocation	false
24) JD Android Geofences - 8/30/24 (enter)	geolocation	false
25) JD iOS Geofences - 8/30/24 (enter)	geolocation	false

26) JH Geofences - 8/29/24 (enter)	geolocation	false
27) JT Geofences - 8/13/24 (enter)	geolocation	false
28) JT Geofences - 09/4/24 (enter)	geolocation	false
29) JW Geofences - 8/30/24 (enter)	geolocation	false
30) JD Android Geofences - 8/30/24 (enter) (Copy)	geolocation	false
31) JT Geofences - 10/11/24 (enter)	geolocation	false
32) JH Geofences - 10/11/24 (enter)	geolocation	false
33) JW Geofences - 10/11/24 (enter)	geolocation	false
34) JH Geofences - 10/16/24 (enter)	geolocation	false
35) JD Android Geofences - 10/16/24 (enter)	geolocation	false
36) JT Geofences - 10/16/24 (enter)	geolocation	false
37) spbm_ftp102 _Geofences	geolocation	false
38) spbm_ftp112_Geofences	geolocation	false
39) spbm_ftp113_Geofences	geolocation	false
40) spbm_ftp121_Geofences	geolocation	false
41) spbm_ftp275_Geofences	geolocation	false
42) SPBM_FTP179_Geofences	geolocation	false
43) SPBM_FTP172_Geofences	geolocation	false
44) SPBM_FTP477_Geofences	geolocation	false
45) SPBM_FTP449_Geofences	geolocation	false
46) SPBM_FTP482_Geofences	geolocation	false
47) SPBM_FTP483_Geofences	geolocation	false
48) SPBM_FTP479_Geofences	geolocation	false
49) SPBM_FTP495_Geofences	geolocation	false

50) SPBM_FTP271_Geofences	geolocation	false

Question & Settings	Question Type	Variable Name	Global Variable Name
12) With how many people?	SINGLE_CHOICE	gf_social3	G:LA:gf_social3

- Display Conditions: (If With others? IS Yes)
- Question Groups: None
- Response Required: Yes
- Randomize Choice: No Choices
- Seconds to Answer: 0
- Seconds Until Next Button Becomes Active: 0

Breakdown of Each Trigger

Name	Type	Settings
1)	ONCE	Runs from 2024-04-08T11:49:00 UNTIL 2025-04-08T00:00:00.000Z
2) Geofence example trigger	schedule	false
3) UserId: 6567c7d46b8a38a70237d196 - Geofences	geolocation	false
4) UserId: 6567cad06b8a38a70237d4dd - Geofences	geolocation	false
5) UserId: 6567cbae6b8a38a70237d532 - Geofences	geolocation	false
6) UserId: 6568bdd76b8a38a70238648b - Geofences	geolocation	false
7) UserId: 6584306828f1dc791eae5abb - Geofences	geolocation	false

8) UserId: 65b0080e29fd6a9d3466f2b5 - Geofences	geolocation	false
9) UserId: 65d0bc2f38a1f8159ed8253b - Geofences	geolocation	false
10) UserId: 65d8d947f7c6ed301283576d - Geofences	geolocation	false
11) UserId: 65dacd5217ddbc9fec6fdade - Geofences	geolocation	false
12) UserId: 65dc2026cc4061e42221d67a - Geofences	geolocation	false
13) UserId: 65de57eb405d00d76d2f03ae - Geofences	geolocation	false
14) UserId: 6660a99c4bbb1eceb296728f - Geofences	geolocation	false
15) UserId: 6658a32b70d761aec4591e9a - Geofences	geolocation	false
16) UserId: 6660a3014bbb1eceb29666bd - Geofences	geolocation	false
17) JT - UserId: 66914ab785c52222d31cd6d1- Geofences	geolocation	false
18) JW Geofences - 8/13/24	geolocation	false
19) JT Geofences - 8/13/24	geolocation	false
20) JH Geofences - 8/29/24	geolocation	false
21) JW Geofences - 8/30/24	geolocation	false
22) JD iOS Geofences - 8/30/24	geolocation	false
23) JD Android Geofences - 8/30/24	geolocation	false
24) JD Android Geofences - 8/30/24 (enter)	geolocation	false

25) JD iOS Geofences - 8/30/24 (enter)	geolocation	false
26) JH Geofences - 8/29/24 (enter)	geolocation	false
27) JT Geofences - 8/13/24 (enter)	geolocation	false
28) JT Geofences - 09/4/24 (enter)	geolocation	false
29) JW Geofences - 8/30/24 (enter)	geolocation	false
30) JD Android Geofences - 8/30/24 (enter) (Copy)	geolocation	false
31) JT Geofences - 10/11/24 (enter)	geolocation	false
32) JH Geofences - 10/11/24 (enter)	geolocation	false
33) JW Geofences - 10/11/24 (enter)	geolocation	false
34) JH Geofences - 10/16/24 (enter)	geolocation	false
35) JD Android Geofences - 10/16/24 (enter)	geolocation	false
36) JT Geofences - 10/16/24 (enter)	geolocation	false
37) spbm_ftp102 _Geofences	geolocation	false
38) spbm_ftp112_Geofences	geolocation	false
39) spbm_ftp113_Geofences	geolocation	false
40) spbm_ftp121_Geofences	geolocation	false
41) spbm_ftp275_Geofences	geolocation	false
42) SPBM_FTP179_Geofences	geolocation	false
43) SPBM_FTP172_Geofences	geolocation	false
44) SPBM_FTP477_Geofences	geolocation	false
45) SPBM_FTP449_Geofences	geolocation	false
46) SPBM_FTP482_Geofences	geolocation	false
47) SPBM_FTP483_Geofences	geolocation	false
48) SPBM_FTP479_Geofences	geolocation	false

49) SPBM_FTP495_Geofences	geolocation	false
50) SPBM_FTP271_Geofences	geolocation	false

Question & Settings	Question Type	Variable Name	Global Variable Name
13) With how many people?	DROPDOWN	gf_social3_dropdown	G:LA:gf_social3_dropdown

- Display Conditions: If With others? IS Yes
- Question Groups: None
- Response Required: Yes
- Randomize Choice: No Choices
- Seconds to Answer: 0
- Seconds Until Next Button Becomes Active: 0

Breakdown of Each Trigger

Name	Type	Settings
1)	ONCE	Runs from 2024-04-08T11:49:00 UNTIL 2025-04-08T00:00:00.000Z
2) Geofence example trigger	schedule	false
3) UserId: 6567c7d46b8a38a70237d196 - Geofences	geolocation	false
4) UserId: 6567cad06b8a38a70237d4dd - Geofences	geolocation	false
5) UserId: 6567cbae6b8a38a70237d532 - Geofences	geolocation	false
6) UserId: 6568bdd76b8a38a70238648b - Geofences	geolocation	false
7) UserId: 6584306828f1dc791eae5abb -	geolocation	false

Geofences		
8) UserId: 65b0080e29fd6a9d3466f2b5 - Geofences	geolocation	false
9) UserId: 65d0bc2f38a1f8159ed8253b - Geofences	geolocation	false
10) UserId: 65d8d947f7c6ed301283576d - Geofences	geolocation	false
11) UserId: 65dacd5217ddbc9fec6fdade - Geofences	geolocation	false
12) UserId: 65dc2026cc4061e42221d67a - Geofences	geolocation	false
13) UserId: 65de57eb405d00d76d2f03ae - Geofences	geolocation	false
14) UserId: 6660a99c4bbb1eceb296728f - Geofences	geolocation	false
15) UserId: 6658a32b70d761aec4591e9a - Geofences	geolocation	false
16) UserId: 6660a3014bbb1eceb29666bd - Geofences	geolocation	false
17) JT - UserId: 66914ab785c52222d31cd6d1- Geofences	geolocation	false
18) JW Geofences - 8/13/24	geolocation	false
19) JT Geofences - 8/13/24	geolocation	false
20) JH Geofences - 8/29/24	geolocation	false
21) JW Geofences - 8/30/24	geolocation	false
22) JD iOS Geofences - 8/30/24	geolocation	false
23) JD Android Geofences - 8/30/24	geolocation	false

24) JD Android Geofences - 8/30/24 (enter)	geolocation	false
25) JD iOS Geofences - 8/30/24 (enter)	geolocation	false
26) JH Geofences - 8/29/24 (enter)	geolocation	false
27) JT Geofences - 8/13/24 (enter)	geolocation	false
28) JT Geofences - 09/4/24 (enter)	geolocation	false
29) JW Geofences - 8/30/24 (enter)	geolocation	false
30) JD Android Geofences - 8/30/24 (enter) (Copy)	geolocation	false
31) JT Geofences - 10/11/24 (enter)	geolocation	false
32) JH Geofences - 10/11/24 (enter)	geolocation	false
33) JW Geofences - 10/11/24 (enter)	geolocation	false
34) JH Geofences - 10/16/24 (enter)	geolocation	false
35) JD Android Geofences - 10/16/24 (enter)	geolocation	false
36) JT Geofences - 10/16/24 (enter)	geolocation	false
37) spbm_ftp102 _Geofences	geolocation	false
38) spbm_ftp112_Geofences	geolocation	false
39) spbm_ftp113_Geofences	geolocation	false
40) spbm_ftp121_Geofences	geolocation	false
41) spbm_ftp275_Geofences	geolocation	false
42) SPBM_FTP179_Geofences	geolocation	false
43) SPBM_FTP172_Geofences	geolocation	false
44) SPBM_FTP477_Geofences	geolocation	false
45) SPBM_FTP449_Geofences	geolocation	false
46) SPBM_FTP482_Geofences	geolocation	false
47) SPBM_FTP483_Geofences	geolocation	false

48) SPBM_FTP479_Geofences	geolocation	false
49) SPBM_FTP495_Geofences	geolocation	false
50) SPBM_FTP271_Geofences	geolocation	false

Question & Settings	Question Type	Variable Name	Global Variable Name
14) Other people smoking cigarettes or using other tobacco products?	SINGLE_CHOICE	gf_social4	G:LA:gf_social4

- Display Conditions: If With others? IS Yes
- Question Groups: None
- Response Required: Yes
- Randomize Choice: No Choices
- Seconds to Answer: 0
- Seconds Until Next Button Becomes Active: 0

Breakdown of Each Trigger

Name	Type	Settings
1)	ONCE	Runs from 2024-04-08T11:49:00 UNTIL 2025-04-08T00:00:00.000Z
2) Geofence example trigger	schedule	false
3) UserId: 6567c7d46b8a38a70237d196 - Geofences	geolocation	false
4) UserId: 6567cad06b8a38a70237d4dd - Geofences	geolocation	false
5) UserId: 6567cbae6b8a38a70237d532 - Geofences	geolocation	false

6) UserId: 6568bdd76b8a38a70238648b - Geofences	geolocation	false
7) UserId: 6584306828f1dc791eae5abb - Geofences	geolocation	false
8) UserId: 65b0080e29fd6a9d3466f2b5 - Geofences	geolocation	false
9) UserId: 65d0bc2f38a1f8159ed8253b - Geofences	geolocation	false
10) UserId: 65d8d947f7c6ed301283576d - Geofences	geolocation	false
11) UserId: 65dacd5217ddbc9fec6fdade - Geofences	geolocation	false
12) UserId: 65dc2026cc4061e42221d67a - Geofences	geolocation	false
13) UserId: 65de57eb405d00d76d2f03ae - Geofences	geolocation	false
14) UserId: 6660a99c4bbb1eceb296728f - Geofences	geolocation	false
15) UserId: 6658a32b70d761aec4591e9a - Geofences	geolocation	false
16) UserId: 6660a3014bbb1eceb29666bd - Geofences	geolocation	false
17) JT - UserId: 66914ab785c52222d31cd6d1- Geofences	geolocation	false
18) JW Geofences - 8/13/24	geolocation	false
19) JT Geofences - 8/13/24	geolocation	false
20) JH Geofences - 8/29/24	geolocation	false

21) JW Geofences - 8/30/24	geolocation	false
22) JD iOS Geofences - 8/30/24	geolocation	false
23) JD Android Geofences - 8/30/24	geolocation	false
24) JD Android Geofences - 8/30/24 (enter)	geolocation	false
25) JD iOS Geofences - 8/30/24 (enter)	geolocation	false
26) JH Geofences - 8/29/24 (enter)	geolocation	false
27) JT Geofences - 8/13/24 (enter)	geolocation	false
28) JT Geofences - 09/4/24 (enter)	geolocation	false
29) JW Geofences - 8/30/24 (enter)	geolocation	false
30) JD Android Geofences - 8/30/24 (enter) (Copy)	geolocation	false
31) JT Geofences - 10/11/24 (enter)	geolocation	false
32) JH Geofences - 10/11/24 (enter)	geolocation	false
33) JW Geofences - 10/11/24 (enter)	geolocation	false
34) JH Geofences - 10/16/24 (enter)	geolocation	false
35) JD Android Geofences - 10/16/24 (enter)	geolocation	false
36) JT Geofences - 10/16/24 (enter)	geolocation	false
37) spbm_ftp102 _Geofences	geolocation	false
38) spbm_ftp112_Geofences	geolocation	false
39) spbm_ftp113_Geofences	geolocation	false
40) spbm_ftp121_Geofences	geolocation	false
41) spbm_ftp275_Geofences	geolocation	false
42) SPBM_FTP179_Geofences	geolocation	false
43) SPBM_FTP172_Geofences	geolocation	false
44) SPBM_FTP477_Geofences	geolocation	false

45) SPBM_FTP449_Geofences	geolocation	false
46) SPBM_FTP482_Geofences	geolocation	false
47) SPBM_FTP483_Geofences	geolocation	false
48) SPBM_FTP479_Geofences	geolocation	false
49) SPBM_FTP495_Geofences	geolocation	false
50) SPBM_FTP271_Geofences	geolocation	false

Question & Settings	Question Type	Variable Name	Global Variable Name
15) Who was smoking cigarettes or using other tobacco products?	MULTIPLE_CHOICE	gf_social5	G:LA:gf_social5

- Display Conditions: If Other people smoking cigarettes or using other tobacco products? IS Yes
- Question Groups: None
- Response Required: Yes
- Randomize Choice: No Choices
- Seconds to Answer: 0
- Seconds Until Next Button Becomes Active: 0

Breakdown of Each Trigger

Name	Type	Settings
1)	ONCE	Runs from 2024-04-08T11:49:00 UNTIL 2025-04-08T00:00:00.000Z
2) Geofence example trigger	schedule	false
3) UserId: 6567c7d46b8a38a70237d196 - Geofences	geolocation	false
4) UserId: 6567cad06b8a38a70237d4dd - Geofences	geolocation	false

5) UserId: 6567cbae6b8a38a70237d532 - Geofences	geolocation	false
6) UserId: 6568bdd76b8a38a70238648b - Geofences	geolocation	false
7) UserId: 6584306828f1dc791eae5abb - Geofences	geolocation	false
8) UserId: 65b0080e29fd6a9d3466f2b5 - Geofences	geolocation	false
9) UserId: 65d0bc2f38a1f8159ed8253b - Geofences	geolocation	false
10) UserId: 65d8d947f7c6ed301283576d - Geofences	geolocation	false
11) UserId: 65dacd5217ddbc9fec6fdade - Geofences	geolocation	false
12) UserId: 65dc2026cc4061e42221d67a - Geofences	geolocation	false
13) UserId: 65de57eb405d00d76d2f03ae - Geofences	geolocation	false
14) UserId: 6660a99c4bbb1eceb296728f - Geofences	geolocation	false
15) UserId: 6658a32b70d761aec4591e9a - Geofences	geolocation	false
16) UserId: 6660a3014bbb1eceb29666bd - Geofences	geolocation	false
17) JT - UserId: 66914ab785c52222d31cd6d1- Geofences	geolocation	false
18) JW Geofences - 8/13/24	geolocation	false
19) JT Geofences - 8/13/24	geolocation	false

20) JH Geofences - 8/29/24	geolocation	false
21) JW Geofences - 8/30/24	geolocation	false
22) JD iOS Geofences - 8/30/24	geolocation	false
23) JD Android Geofences - 8/30/24	geolocation	false
24) JD Android Geofences - 8/30/24 (enter)	geolocation	false
25) JD iOS Geofences - 8/30/24 (enter)	geolocation	false
26) JH Geofences - 8/29/24 (enter)	geolocation	false
27) JT Geofences - 8/13/24 (enter)	geolocation	false
28) JT Geofences - 09/4/24 (enter)	geolocation	false
29) JW Geofences - 8/30/24 (enter)	geolocation	false
30) JD Android Geofences - 8/30/24 (enter) (Copy)	geolocation	false
31) JT Geofences - 10/11/24 (enter)	geolocation	false
32) JH Geofences - 10/11/24 (enter)	geolocation	false
33) JW Geofences - 10/11/24 (enter)	geolocation	false
34) JH Geofences - 10/16/24 (enter)	geolocation	false
35) JD Android Geofences - 10/16/24 (enter)	geolocation	false
36) JT Geofences - 10/16/24 (enter)	geolocation	false
37) spbm_ftp102 _Geofences	geolocation	false
38) spbm_ftp112_Geofences	geolocation	false
39) spbm_ftp113_Geofences	geolocation	false
40) spbm_ftp121_Geofences	geolocation	false
41) spbm_ftp275_Geofences	geolocation	false
42) SPBM_FTP179_Geofences	geolocation	false
43) SPBM_FTP172_Geofences	geolocation	false

44) SPBM_FTP477_Geofences	geolocation	false
45) SPBM_FTP449_Geofences	geolocation	false
46) SPBM_FTP482_Geofences	geolocation	false
47) SPBM_FTP483_Geofences	geolocation	false
48) SPBM_FTP479_Geofences	geolocation	false
49) SPBM_FTP495_Geofences	geolocation	false
50) SPBM_FTP271_Geofences	geolocation	false

Question & Settings	Question Type	Variable Name	Global Variable Name
16) How many people were smoking cigarettes or using other tobacco products?	SINGLE_CHOICE	gf_social6	G:LA:gf_social6

- Display Conditions: If Other people smoking cigarettes or using other tobacco products? IS Yes
- Question Groups: None
- Response Required: Yes
- Randomize Choice: No Choices
- Seconds to Answer: 0
- Seconds Until Next Button Becomes Active: 0

Breakdown of Each Trigger

Name	Type	Settings
1)	ONCE	Runs from 2024-04-08T11:49:00 UNTIL 2025-04-08T00:00:00.000Z
2) Geofence example trigger	schedule	false
3) UserId: 6567c7d46b8a38a70237d196 - Geofences	geolocation	false

4) UserId: 6567cad06b8a38a70237d4dd - Geofences	geolocation	false
5) UserId: 6567cbae6b8a38a70237d532 - Geofences	geolocation	false
6) UserId: 6568bdd76b8a38a70238648b - Geofences	geolocation	false
7) UserId: 6584306828f1dc791eae5abb - Geofences	geolocation	false
8) UserId: 65b0080e29fd6a9d3466f2b5 - Geofences	geolocation	false
9) UserId: 65d0bc2f38a1f8159ed8253b - Geofences	geolocation	false
10) UserId: 65d8d947f7c6ed301283576d - Geofences	geolocation	false
11) UserId: 65dacd5217ddbc9fec6fdade - Geofences	geolocation	false
12) UserId: 65dc2026cc4061e42221d67a - Geofences	geolocation	false
13) UserId: 65de57eb405d00d76d2f03ae - Geofences	geolocation	false
14) UserId: 6660a99c4bbb1eceb296728f - Geofences	geolocation	false
15) UserId: 6658a32b70d761aec4591e9a - Geofences	geolocation	false
16) UserId: 6660a3014bbb1eceb29666bd - Geofences	geolocation	false
17) JT - UserId: 66914ab785c52222d31cd6d1- Geofences	geolocation	false

18) JW Geofences - 8/13/24	geolocation	false
19) JT Geofences - 8/13/24	geolocation	false
20) JH Geofences - 8/29/24	geolocation	false
21) JW Geofences - 8/30/24	geolocation	false
22) JD iOS Geofences - 8/30/24	geolocation	false
23) JD Android Geofences - 8/30/24	geolocation	false
24) JD Android Geofences - 8/30/24 (enter)	geolocation	false
25) JD iOS Geofences - 8/30/24 (enter)	geolocation	false
26) JH Geofences - 8/29/24 (enter)	geolocation	false
27) JT Geofences - 8/13/24 (enter)	geolocation	false
28) JT Geofences - 09/4/24 (enter)	geolocation	false
29) JW Geofences - 8/30/24 (enter)	geolocation	false
30) JD Android Geofences - 8/30/24 (enter) (Copy)	geolocation	false
31) JT Geofences - 10/11/24 (enter)	geolocation	false
32) JH Geofences - 10/11/24 (enter)	geolocation	false
33) JW Geofences - 10/11/24 (enter)	geolocation	false
34) JH Geofences - 10/16/24 (enter)	geolocation	false
35) JD Android Geofences - 10/16/24 (enter)	geolocation	false
36) JT Geofences - 10/16/24 (enter)	geolocation	false
37) spbm_ftp102 _Geofences	geolocation	false
38) spbm_ftp112_Geofences	geolocation	false
39) spbm_ftp113_Geofences	geolocation	false
40) spbm_ftp121_Geofences	geolocation	false
41) spbm_ftp275_Geofences	geolocation	false

42) SPBM_FTP179_Geofences	geolocation	false
43) SPBM_FTP172_Geofences	geolocation	false
44) SPBM_FTP477_Geofences	geolocation	false
45) SPBM_FTP449_Geofences	geolocation	false
46) SPBM_FTP482_Geofences	geolocation	false
47) SPBM_FTP483_Geofences	geolocation	false
48) SPBM_FTP479_Geofences	geolocation	false
49) SPBM_FTP495_Geofences	geolocation	false
50) SPBM_FTP271_Geofences	geolocation	false

Question & Settings	Question Type	Variable Name	Global Variable Name
17) How many people were smoking cigarettes or using other tobacco products?	DROPDOWN	gf_social6_dropdown	G:LA:gf_social6_dropdown

- Display Conditions: If Other people smoking cigarettes or using other tobacco products? IS Yes
- Question Groups: None
- Response Required: Yes
- Randomize Choice: No Choices
- Seconds to Answer: 0
- Seconds Until Next Button Becomes Active: 0

Breakdown of Each Trigger

Name	Type	Settings
1)	ONCE	Runs from 2024-04-08T11:49:00 UNTIL 2025-04-08T00:00:00.000Z

2) Geofence example trigger	schedule	false
3) UserId: 6567c7d46b8a38a70237d196 - Geofences	geolocation	false
4) UserId: 6567cad06b8a38a70237d4dd - Geofences	geolocation	false
5) UserId: 6567cbae6b8a38a70237d532 - Geofences	geolocation	false
6) UserId: 6568bdd76b8a38a70238648b - Geofences	geolocation	false
7) UserId: 6584306828f1dc791eae5abb - Geofences	geolocation	false
8) UserId: 65b0080e29fd6a9d3466f2b5 - Geofences	geolocation	false
9) UserId: 65d0bc2f38a1f8159ed8253b - Geofences	geolocation	false
10) UserId: 65d8d947f7c6ed301283576d - Geofences	geolocation	false
11) UserId: 65dacd5217ddbc9fec6fdade - Geofences	geolocation	false
12) UserId: 65dc2026cc4061e42221d67a - Geofences	geolocation	false
13) UserId: 65de57eb405d00d76d2f03ae - Geofences	geolocation	false
14) UserId: 6660a99c4bbb1eceb296728f - Geofences	geolocation	false
15) UserId: 6658a32b70d761aec4591e9a - Geofences	geolocation	false
16) UserId: 6660a3014bbb1eceb29666bd -	geolocation	false

Geofences		
17) JT - UserId: 66914ab785c52222d31cd6d1- Geofences	geolocation	false
18) JW Geofences - 8/13/24	geolocation	false
19) JT Geofences - 8/13/24	geolocation	false
20) JH Geofences - 8/29/24	geolocation	false
21) JW Geofences - 8/30/24	geolocation	false
22) JD iOS Geofences - 8/30/24	geolocation	false
23) JD Android Geofences - 8/30/24	geolocation	false
24) JD Android Geofences - 8/30/24 (enter)	geolocation	false
25) JD iOS Geofences - 8/30/24 (enter)	geolocation	false
26) JH Geofences - 8/29/24 (enter)	geolocation	false
27) JT Geofences - 8/13/24 (enter)	geolocation	false
28) JT Geofences - 09/4/24 (enter)	geolocation	false
29) JW Geofences - 8/30/24 (enter)	geolocation	false
30) JD Android Geofences - 8/30/24 (enter) (Copy)	geolocation	false
31) JT Geofences - 10/11/24 (enter)	geolocation	false
32) JH Geofences - 10/11/24 (enter)	geolocation	false
33) JW Geofences - 10/11/24 (enter)	geolocation	false
34) JH Geofences - 10/16/24 (enter)	geolocation	false
35) JD Android Geofences - 10/16/24 (enter)	geolocation	false
36) JT Geofences - 10/16/24 (enter)	geolocation	false
37) spbm_ftp102 _Geofences	geolocation	false
38) spbm_ftp112_Geofences	geolocation	false

39) spbm_ftp113_Geofences	geolocation	false
40) spbm_ftp121_Geofences	geolocation	false
41) spbm_ftp275_Geofences	geolocation	false
42) SPBM_FTP179_Geofences	geolocation	false
43) SPBM_FTP172_Geofences	geolocation	false
44) SPBM_FTP477_Geofences	geolocation	false
45) SPBM_FTP449_Geofences	geolocation	false
46) SPBM_FTP482_Geofences	geolocation	false
47) SPBM_FTP483_Geofences	geolocation	false
48) SPBM_FTP479_Geofences	geolocation	false
49) SPBM_FTP495_Geofences	geolocation	false
50) SPBM_FTP271_Geofences	geolocation	false

Question & Settings	Question Type	Variable Name	Global Variable Name
18) What were you doing?	SINGLE_CHOICE	gf_activity	G:LA:gf_activity

- Display Conditions: None
- Question Groups: None
- Response Required: Yes
- Randomize Choice: No Choices
- Seconds to Answer: 0
- Seconds Until Next Button Becomes Active: 0

Breakdown of Each Trigger

Name	Type	Settings
1)	ONCE	Runs from 2024-04-08T11:49:00 UNTIL

		2025-04-08T00:00:00.000Z
2) Geofence example trigger	schedule	false
3) UserId: 6567c7d46b8a38a70237d196 - Geofences	geolocation	false
4) UserId: 6567cad06b8a38a70237d4dd - Geofences	geolocation	false
5) UserId: 6567cbae6b8a38a70237d532 - Geofences	geolocation	false
6) UserId: 6568bdd76b8a38a70238648b - Geofences	geolocation	false
7) UserId: 6584306828f1dc791eae5abb - Geofences	geolocation	false
8) UserId: 65b0080e29fd6a9d3466f2b5 - Geofences	geolocation	false
9) UserId: 65d0bc2f38a1f8159ed8253b - Geofences	geolocation	false
10) UserId: 65d8d947f7c6ed301283576d - Geofences	geolocation	false
11) UserId: 65dacd5217ddbc9fec6fdade - Geofences	geolocation	false
12) UserId: 65dc2026cc4061e42221d67a - Geofences	geolocation	false
13) UserId: 65de57eb405d00d76d2f03ae - Geofences	geolocation	false
14) UserId: 6660a99c4bbb1eceb296728f - Geofences	geolocation	false
15) UserId: 6658a32b70d761aec4591e9a - Geofences	geolocation	false

16) UserId: 6660a3014bbb1eceb29666bd - Geofences	geolocation	false
17) JT - UserId: 66914ab785c52222d31cd6d1- Geofences	geolocation	false
18) JW Geofences - 8/13/24	geolocation	false
19) JT Geofences - 8/13/24	geolocation	false
20) JH Geofences - 8/29/24	geolocation	false
21) JW Geofences - 8/30/24	geolocation	false
22) JD iOS Geofences - 8/30/24	geolocation	false
23) JD Android Geofences - 8/30/24	geolocation	false
24) JD Android Geofences - 8/30/24 (enter)	geolocation	false
25) JD iOS Geofences - 8/30/24 (enter)	geolocation	false
26) JH Geofences - 8/29/24 (enter)	geolocation	false
27) JT Geofences - 8/13/24 (enter)	geolocation	false
28) JT Geofences - 09/4/24 (enter)	geolocation	false
29) JW Geofences - 8/30/24 (enter)	geolocation	false
30) JD Android Geofences - 8/30/24 (enter) (Copy)	geolocation	false
31) JT Geofences - 10/11/24 (enter)	geolocation	false
32) JH Geofences - 10/11/24 (enter)	geolocation	false
33) JW Geofences - 10/11/24 (enter)	geolocation	false
34) JH Geofences - 10/16/24 (enter)	geolocation	false
35) JD Android Geofences - 10/16/24 (enter)	geolocation	false
36) JT Geofences - 10/16/24 (enter)	geolocation	false
37) spbm_ftp102 _Geofences	geolocation	false

38) spbm_ftp112_Geofences	geolocation	false
39) spbm_ftp113_Geofences	geolocation	false
40) spbm_ftp121_Geofences	geolocation	false
41) spbm_ftp275_Geofences	geolocation	false
42) SPBM_FTP179_Geofences	geolocation	false
43) SPBM_FTP172_Geofences	geolocation	false
44) SPBM_FTP477_Geofences	geolocation	false
45) SPBM_FTP449_Geofences	geolocation	false
46) SPBM_FTP482_Geofences	geolocation	false
47) SPBM_FTP483_Geofences	geolocation	false
48) SPBM_FTP479_Geofences	geolocation	false
49) SPBM_FTP495_Geofences	geolocation	false
50) SPBM_FTP271_Geofences	geolocation	false

Question & Settings	Question Type	Variable Name	Global Variable Name
19) What other activities?	TEXT	gf_activityother	G:LA:gf_activityother

- Display Conditions: If IS
- Question Groups: None
- Response Required: Yes
- Randomize Choice: No Choices
- Seconds to Answer: 0
- Seconds Until Next Button Becomes Active: 0

Breakdown of Each Trigger

Name	Type	Settings

1)	ONCE	Runs from 2024-04-08T11:49:00 UNTIL 2025-04-08T00:00:00.000Z
2) Geofence example trigger	schedule	false
3) UserId: 6567c7d46b8a38a70237d196 - Geofences	geolocation	false
4) UserId: 6567cad06b8a38a70237d4dd - Geofences	geolocation	false
5) UserId: 6567cbae6b8a38a70237d532 - Geofences	geolocation	false
6) UserId: 6568bdd76b8a38a70238648b - Geofences	geolocation	false
7) UserId: 6584306828f1dc791eae5abb - Geofences	geolocation	false
8) UserId: 65b0080e29fd6a9d3466f2b5 - Geofences	geolocation	false
9) UserId: 65d0bc2f38a1f8159ed8253b - Geofences	geolocation	false
10) UserId: 65d8d947f7c6ed301283576d - Geofences	geolocation	false
11) UserId: 65dacd5217ddbc9fec6fdade - Geofences	geolocation	false
12) UserId: 65dc2026cc4061e42221d67a - Geofences	geolocation	false
13) UserId: 65de57eb405d00d76d2f03ae - Geofences	geolocation	false
14) UserId: 6660a99c4bbb1eceb296728f - Geofences	geolocation	false
15) UserId: 6658a32b70d761aec4591e9a -	geolocation	false

Geofences		
16) UserId: 6660a3014bbb1eceb29666bd - Geofences	geolocation	false
17) JT - UserId: 66914ab785c52222d31cd6d1- Geofences	geolocation	false
18) JW Geofences - 8/13/24	geolocation	false
19) JT Geofences - 8/13/24	geolocation	false
20) JH Geofences - 8/29/24	geolocation	false
21) JW Geofences - 8/30/24	geolocation	false
22) JD iOS Geofences - 8/30/24	geolocation	false
23) JD Android Geofences - 8/30/24	geolocation	false
24) JD Android Geofences - 8/30/24 (enter)	geolocation	false
25) JD iOS Geofences - 8/30/24 (enter)	geolocation	false
26) JH Geofences - 8/29/24 (enter)	geolocation	false
27) JT Geofences - 8/13/24 (enter)	geolocation	false
28) JT Geofences - 09/4/24 (enter)	geolocation	false
29) JW Geofences - 8/30/24 (enter)	geolocation	false
30) JD Android Geofences - 8/30/24 (enter) (Copy)	geolocation	false
31) JT Geofences - 10/11/24 (enter)	geolocation	false
32) JH Geofences - 10/11/24 (enter)	geolocation	false
33) JW Geofences - 10/11/24 (enter)	geolocation	false
34) JH Geofences - 10/16/24 (enter)	geolocation	false
35) JD Android Geofences - 10/16/24 (enter)	geolocation	false
36) JT Geofences - 10/16/24 (enter)	geolocation	false

37) spbm_ftp102 _Geofences	geolocation	false
38) spbm_ftp112_Geofences	geolocation	false
39) spbm_ftp113_Geofences	geolocation	false
40) spbm_ftp121_Geofences	geolocation	false
41) spbm_ftp275_Geofences	geolocation	false
42) SPBM_FTP179_Geofences	geolocation	false
43) SPBM_FTP172_Geofences	geolocation	false
44) SPBM_FTP477_Geofences	geolocation	false
45) SPBM_FTP449_Geofences	geolocation	false
46) SPBM_FTP482_Geofences	geolocation	false
47) SPBM_FTP483_Geofences	geolocation	false
48) SPBM_FTP479_Geofences	geolocation	false
49) SPBM_FTP495_Geofences	geolocation	false
50) SPBM_FTP271_Geofences	geolocation	false

Question & Settings	Question Type	Variable Name	Global Variable Name
20) Consume any of the following in last hour?	MULTIPLE_CHOICE	gf_consume	G:LA:gf_consume

- Display Conditions: None
- Question Groups: None
- Response Required: Yes
- Randomize Choice: No Choices
- Seconds to Answer: 0
- Seconds Until Next Button Becomes Active: 0

Breakdown of Each Trigger

Name	Type	Settings
1)	ONCE	Runs from 2024-04-08T11:49:00 UNTIL 2025-04-08T00:00:00.000Z
2) Geofence example trigger	schedule	false
3) UserId: 6567c7d46b8a38a70237d196 - Geofences	geolocation	false
4) UserId: 6567cad06b8a38a70237d4dd - Geofences	geolocation	false
5) UserId: 6567cbae6b8a38a70237d532 - Geofences	geolocation	false
6) UserId: 6568bdd76b8a38a70238648b - Geofences	geolocation	false
7) UserId: 6584306828f1dc791eae5abb - Geofences	geolocation	false
8) UserId: 65b0080e29fd6a9d3466f2b5 - Geofences	geolocation	false
9) UserId: 65d0bc2f38a1f8159ed8253b - Geofences	geolocation	false
10) UserId: 65d8d947f7c6ed301283576d - Geofences	geolocation	false
11) UserId: 65dacd5217ddbc9fec6fdade - Geofences	geolocation	false
12) UserId: 65dc2026cc4061e42221d67a - Geofences	geolocation	false
13) UserId: 65de57eb405d00d76d2f03ae - Geofences	geolocation	false
14) UserId: 6660a99c4bbb1eceb296728f -	geolocation	false

Geofences		
15) UserId: 6658a32b70d761aec4591e9a - Geofences	geolocation	false
16) UserId: 6660a3014bbb1eceb29666bd - Geofences	geolocation	false
17) JT - UserId: 66914ab785c52222d31cd6d1- Geofences	geolocation	false
18) JW Geofences - 8/13/24	geolocation	false
19) JT Geofences - 8/13/24	geolocation	false
20) JH Geofences - 8/29/24	geolocation	false
21) JW Geofences - 8/30/24	geolocation	false
22) JD iOS Geofences - 8/30/24	geolocation	false
23) JD Android Geofences - 8/30/24	geolocation	false
24) JD Android Geofences - 8/30/24 (enter)	geolocation	false
25) JD iOS Geofences - 8/30/24 (enter)	geolocation	false
26) JH Geofences - 8/29/24 (enter)	geolocation	false
27) JT Geofences - 8/13/24 (enter)	geolocation	false
28) JT Geofences - 09/4/24 (enter)	geolocation	false
29) JW Geofences - 8/30/24 (enter)	geolocation	false
30) JD Android Geofences - 8/30/24 (enter) (Copy)	geolocation	false
31) JT Geofences - 10/11/24 (enter)	geolocation	false
32) JH Geofences - 10/11/24 (enter)	geolocation	false
33) JW Geofences - 10/11/24 (enter)	geolocation	false
34) JH Geofences - 10/16/24 (enter)	geolocation	false

35) JD Android Geofences - 10/16/24 (enter)	geolocation	false
36) JT Geofences - 10/16/24 (enter)	geolocation	false
37) spbm_ftp102 _Geofences	geolocation	false
38) spbm_ftp112_Geofences	geolocation	false
39) spbm_ftp113_Geofences	geolocation	false
40) spbm_ftp121_Geofences	geolocation	false
41) spbm_ftp275_Geofences	geolocation	false
42) SPBM_FTP179_Geofences	geolocation	false
43) SPBM_FTP172_Geofences	geolocation	false
44) SPBM_FTP477_Geofences	geolocation	false
45) SPBM_FTP449_Geofences	geolocation	false
46) SPBM_FTP482_Geofences	geolocation	false
47) SPBM_FTP483_Geofences	geolocation	false
48) SPBM_FTP479_Geofences	geolocation	false
49) SPBM_FTP495_Geofences	geolocation	false
50) SPBM_FTP271_Geofences	geolocation	false

Question & Settings	Question Type	Variable Name	Global Variable Name
21) Intoxicated and/or drunk?	SINGLE_CHOICE	gf_intoxicated	G:LA:gf_intoxicated

- Display Conditions: None
- Question Groups: None
- Response Required: Yes
- Randomize Choice: No Choices
- Seconds to Answer: 0
- Seconds Until Next Button Becomes Active: 0

Breakdown of Each Trigger

Name	Type	Settings
1)	ONCE	Runs from 2024-04-08T11:49:00 UNTIL 2025-04-08T00:00:00.000Z
2) Geofence example trigger	schedule	false
3) UserId: 6567c7d46b8a38a70237d196 - Geofences	geolocation	false
4) UserId: 6567cad06b8a38a70237d4dd - Geofences	geolocation	false
5) UserId: 6567cbae6b8a38a70237d532 - Geofences	geolocation	false
6) UserId: 6568bdd76b8a38a70238648b - Geofences	geolocation	false
7) UserId: 6584306828f1dc791eae5abb - Geofences	geolocation	false
8) UserId: 65b0080e29fd6a9d3466f2b5 - Geofences	geolocation	false
9) UserId: 65d0bc2f38a1f8159ed8253b - Geofences	geolocation	false
10) UserId: 65d8d947f7c6ed301283576d - Geofences	geolocation	false
11) UserId: 65dacd5217ddbc9fec6fdade - Geofences	geolocation	false
12) UserId: 65dc2026cc4061e42221d67a - Geofences	geolocation	false
13) UserId: 65de57eb405d00d76d2f03ae - Geofences	geolocation	false

14) UserId: 6660a99c4bbb1eceb296728f - Geofences	geolocation	false
15) UserId: 6658a32b70d761aec4591e9a - Geofences	geolocation	false
16) UserId: 6660a3014bbb1eceb29666bd - Geofences	geolocation	false
17) JT - UserId: 66914ab785c52222d31cd6d1- Geofences	geolocation	false
18) JW Geofences - 8/13/24	geolocation	false
19) JT Geofences - 8/13/24	geolocation	false
20) JH Geofences - 8/29/24	geolocation	false
21) JW Geofences - 8/30/24	geolocation	false
22) JD iOS Geofences - 8/30/24	geolocation	false
23) JD Android Geofences - 8/30/24	geolocation	false
24) JD Android Geofences - 8/30/24 (enter)	geolocation	false
25) JD iOS Geofences - 8/30/24 (enter)	geolocation	false
26) JH Geofences - 8/29/24 (enter)	geolocation	false
27) JT Geofences - 8/13/24 (enter)	geolocation	false
28) JT Geofences - 09/4/24 (enter)	geolocation	false
29) JW Geofences - 8/30/24 (enter)	geolocation	false
30) JD Android Geofences - 8/30/24 (enter) (Copy)	geolocation	false
31) JT Geofences - 10/11/24 (enter)	geolocation	false
32) JH Geofences - 10/11/24 (enter)	geolocation	false
33) JW Geofences - 10/11/24 (enter)	geolocation	false
34) JH Geofences - 10/16/24 (enter)	geolocation	false

35) JD Android Geofences - 10/16/24 (enter)	geolocation	false
36) JT Geofences - 10/16/24 (enter)	geolocation	false
37) spbm_ftp102 _Geofences	geolocation	false
38) spbm_ftp112_Geofences	geolocation	false
39) spbm_ftp113_Geofences	geolocation	false
40) spbm_ftp121_Geofences	geolocation	false
41) spbm_ftp275_Geofences	geolocation	false
42) SPBM_FTP179_Geofences	geolocation	false
43) SPBM_FTP172_Geofences	geolocation	false
44) SPBM_FTP477_Geofences	geolocation	false
45) SPBM_FTP449_Geofences	geolocation	false
46) SPBM_FTP482_Geofences	geolocation	false
47) SPBM_FTP483_Geofences	geolocation	false
48) SPBM_FTP479_Geofences	geolocation	false
49) SPBM_FTP495_Geofences	geolocation	false
50) SPBM_FTP271_Geofences	geolocation	false

Question & Settings	Question Type	Variable Name	Global Variable Name
22) Saw any of the following?	MULTIPLE_CHOICE	gf_exposed	G:LA:gf_exposed

- Display Conditions: None
- Question Groups: None
- Response Required: Yes
- Randomize Choice: No Choices
- Seconds to Answer: 0
- Seconds Until Next Button Becomes Active: 0

Breakdown of Each Trigger

Name	Type	Settings
1)	ONCE	Runs from 2024-04-08T11:49:00 UNTIL 2025-04-08T00:00:00.000Z
2) Geofence example trigger	schedule	false
3) UserId: 6567c7d46b8a38a70237d196 - Geofences	geolocation	false
4) UserId: 6567cad06b8a38a70237d4dd - Geofences	geolocation	false
5) UserId: 6567cbae6b8a38a70237d532 - Geofences	geolocation	false
6) UserId: 6568bdd76b8a38a70238648b - Geofences	geolocation	false
7) UserId: 6584306828f1dc791eae5abb - Geofences	geolocation	false
8) UserId: 65b0080e29fd6a9d3466f2b5 - Geofences	geolocation	false
9) UserId: 65d0bc2f38a1f8159ed8253b - Geofences	geolocation	false
10) UserId: 65d8d947f7c6ed301283576d - Geofences	geolocation	false
11) UserId: 65dacd5217ddbc9fec6fdade - Geofences	geolocation	false
12) UserId: 65dc2026cc4061e42221d67a - Geofences	geolocation	false
13) UserId: 65de57eb405d00d76d2f03ae - Geofences	geolocation	false

14) UserId: 6660a99c4bbb1eceb296728f - Geofences	geolocation	false
15) UserId: 6658a32b70d761aec4591e9a - Geofences	geolocation	false
16) UserId: 6660a3014bbb1eceb29666bd - Geofences	geolocation	false
17) JT - UserId: 66914ab785c52222d31cd6d1- Geofences	geolocation	false
18) JW Geofences - 8/13/24	geolocation	false
19) JT Geofences - 8/13/24	geolocation	false
20) JH Geofences - 8/29/24	geolocation	false
21) JW Geofences - 8/30/24	geolocation	false
22) JD iOS Geofences - 8/30/24	geolocation	false
23) JD Android Geofences - 8/30/24	geolocation	false
24) JD Android Geofences - 8/30/24 (enter)	geolocation	false
25) JD iOS Geofences - 8/30/24 (enter)	geolocation	false
26) JH Geofences - 8/29/24 (enter)	geolocation	false
27) JT Geofences - 8/13/24 (enter)	geolocation	false
28) JT Geofences - 09/4/24 (enter)	geolocation	false
29) JW Geofences - 8/30/24 (enter)	geolocation	false
30) JD Android Geofences - 8/30/24 (enter) (Copy)	geolocation	false
31) JT Geofences - 10/11/24 (enter)	geolocation	false
32) JH Geofences - 10/11/24 (enter)	geolocation	false
33) JW Geofences - 10/11/24 (enter)	geolocation	false
34) JH Geofences - 10/16/24 (enter)	geolocation	false

35) JD Android Geofences - 10/16/24 (enter)	geolocation	false
36) JT Geofences - 10/16/24 (enter)	geolocation	false
37) spbm_ftp102 _Geofences	geolocation	false
38) spbm_ftp112_Geofences	geolocation	false
39) spbm_ftp113_Geofences	geolocation	false
40) spbm_ftp121_Geofences	geolocation	false
41) spbm_ftp275_Geofences	geolocation	false
42) SPBM_FTP179_Geofences	geolocation	false
43) SPBM_FTP172_Geofences	geolocation	false
44) SPBM_FTP477_Geofences	geolocation	false
45) SPBM_FTP449_Geofences	geolocation	false
46) SPBM_FTP482_Geofences	geolocation	false
47) SPBM_FTP483_Geofences	geolocation	false
48) SPBM_FTP479_Geofences	geolocation	false
49) SPBM_FTP495_Geofences	geolocation	false
50) SPBM_FTP271_Geofences	geolocation	false

Question & Settings	Question Type	Variable Name	Global Variable Name
23) SINCE THE LAST SURVEY: I felt discriminated against.	SINGLE_CHOICE	gf_discrimination1	G:LA:gf_discrimination1

- Display Conditions: None
- Question Groups: None
- Response Required: Yes
- Randomize Choice: No Choices
- Seconds to Answer: 0
- Seconds Until Next Button Becomes Active: 0

Breakdown of Each Trigger

Name	Type	Settings
1)	ONCE	Runs from 2024-04-08T11:49:00 UNTIL 2025-04-08T00:00:00.000Z
2) Geofence example trigger	schedule	false
3) UserId: 6567c7d46b8a38a70237d196 - Geofences	geolocation	false
4) UserId: 6567cad06b8a38a70237d4dd - Geofences	geolocation	false
5) UserId: 6567cbae6b8a38a70237d532 - Geofences	geolocation	false
6) UserId: 6568bdd76b8a38a70238648b - Geofences	geolocation	false
7) UserId: 6584306828f1dc791eae5abb - Geofences	geolocation	false
8) UserId: 65b0080e29fd6a9d3466f2b5 - Geofences	geolocation	false
9) UserId: 65d0bc2f38a1f8159ed8253b - Geofences	geolocation	false
10) UserId: 65d8d947f7c6ed301283576d - Geofences	geolocation	false
11) UserId: 65dacd5217ddbc9fec6fdade - Geofences	geolocation	false
12) UserId: 65dc2026cc4061e42221d67a - Geofences	geolocation	false

13) UserId: 65de57eb405d00d76d2f03ae - Geofences	geolocation	false
14) UserId: 6660a99c4bbb1eceb296728f - Geofences	geolocation	false
15) UserId: 6658a32b70d761aec4591e9a - Geofences	geolocation	false
16) UserId: 6660a3014bbb1eceb29666bd - Geofences	geolocation	false
17) JT - UserId: 66914ab785c52222d31cd6d1- Geofences	geolocation	false
18) JW Geofences - 8/13/24	geolocation	false
19) JT Geofences - 8/13/24	geolocation	false
20) JH Geofences - 8/29/24	geolocation	false
21) JW Geofences - 8/30/24	geolocation	false
22) JD iOS Geofences - 8/30/24	geolocation	false
23) JD Android Geofences - 8/30/24	geolocation	false
24) JD Android Geofences - 8/30/24 (enter)	geolocation	false
25) JD iOS Geofences - 8/30/24 (enter)	geolocation	false
26) JH Geofences - 8/29/24 (enter)	geolocation	false
27) JT Geofences - 8/13/24 (enter)	geolocation	false
28) JT Geofences - 09/4/24 (enter)	geolocation	false
29) JW Geofences - 8/30/24 (enter)	geolocation	false
30) JD Android Geofences - 8/30/24 (enter) (Copy)	geolocation	false
31) JT Geofences - 10/11/24 (enter)	geolocation	false
32) JH Geofences - 10/11/24 (enter)	geolocation	false

33) JW Geofences - 10/11/24 (enter)	geolocation	false	
34) JH Geofences - 10/16/24 (enter)	geolocation	false	
35) JD Android Geofences - 10/16/24 (enter)	geolocation	false	
36) JT Geofences - 10/16/24 (enter)	geolocation	false	
37) spbm_ftp102 _Geofences	geolocation	false	
38) spbm_ftp112_Geofences	geolocation	false	
39) spbm_ftp113_Geofences	geolocation	false	
40) spbm_ftp121_Geofences	geolocation	false	
41) spbm_ftp275_Geofences	geolocation	false	
42) SPBM_FTP179_Geofences	geolocation	false	
43) SPBM_FTP172_Geofences	geolocation	false	
44) SPBM_FTP477_Geofences	geolocation	false	
45) SPBM_FTP449_Geofences	geolocation	false	
46) SPBM_FTP482_Geofences	geolocation	false	
47) SPBM_FTP483_Geofences	geolocation	false	
48) SPBM_FTP479_Geofences	geolocation	false	
49) SPBM_FTP495_Geofences	geolocation	false	
50) SPBM_FTP271_Geofences	geolocation	false	

Question & Settings	Question Type	Variable Name	Global Variable Name
24) SINCE THE LAST SURVEY: What was the main reason(s) for the discrimination that you experienced?	MULTIPLE_CHOICE	gf_discrimination2	G:LA:gf_discrimination2

- Display Conditions: If SINCE THE LAST SURVEY: I felt discriminated against. IS Yes

- Question Groups: None
- Response Required: Yes
- Randomize Choice: No Choices
- Seconds to Answer: 0
- Seconds Until Next Button Becomes Active: 0

Breakdown of Each Trigger

Name	Type	Settings
1)	ONCE	Runs from 2024-04-08T11:49:00 UNTIL 2025-04-08T00:00:00.000Z
2) Geofence example trigger	schedule	false
3) UserId: 6567c7d46b8a38a70237d196 - Geofences	geolocation	false
4) UserId: 6567cad06b8a38a70237d4dd - Geofences	geolocation	false
5) UserId: 6567cbae6b8a38a70237d532 - Geofences	geolocation	false
6) UserId: 6568bdd76b8a38a70238648b - Geofences	geolocation	false
7) UserId: 6584306828f1dc791eae5abb - Geofences	geolocation	false
8) UserId: 65b0080e29fd6a9d3466f2b5 - Geofences	geolocation	false
9) UserId: 65d0bc2f38a1f8159ed8253b - Geofences	geolocation	false
10) UserId: 65d8d947f7c6ed301283576d - Geofences	geolocation	false

11) UserId: 65dacd5217ddbc9fec6fdade - Geofences	geolocation	false
12) UserId: 65dc2026cc4061e42221d67a - Geofences	geolocation	false
13) UserId: 65de57eb405d00d76d2f03ae - Geofences	geolocation	false
14) UserId: 6660a99c4bbb1eceb296728f - Geofences	geolocation	false
15) UserId: 6658a32b70d761aec4591e9a - Geofences	geolocation	false
16) UserId: 6660a3014bbb1eceb29666bd - Geofences	geolocation	false
17) JT - UserId: 66914ab785c52222d31cd6d1- Geofences	geolocation	false
18) JW Geofences - 8/13/24	geolocation	false
19) JT Geofences - 8/13/24	geolocation	false
20) JH Geofences - 8/29/24	geolocation	false
21) JW Geofences - 8/30/24	geolocation	false
22) JD iOS Geofences - 8/30/24	geolocation	false
23) JD Android Geofences - 8/30/24	geolocation	false
24) JD Android Geofences - 8/30/24 (enter)	geolocation	false
25) JD iOS Geofences - 8/30/24 (enter)	geolocation	false
26) JH Geofences - 8/29/24 (enter)	geolocation	false
27) JT Geofences - 8/13/24 (enter)	geolocation	false
28) JT Geofences - 09/4/24 (enter)	geolocation	false
29) JW Geofences - 8/30/24 (enter)	geolocation	false

30) JD Android Geofences - 8/30/24 (enter) (Copy)	geolocation	false	
31) JT Geofences - 10/11/24 (enter)	geolocation	false	
32) JH Geofences - 10/11/24 (enter)	geolocation	false	
33) JW Geofences - 10/11/24 (enter)	geolocation	false	
34) JH Geofences - 10/16/24 (enter)	geolocation	false	
35) JD Android Geofences - 10/16/24 (enter)	geolocation	false	
36) JT Geofences - 10/16/24 (enter)	geolocation	false	
37) spbm_ftp102 _Geofences	geolocation	false	
38) spbm_ftp112_Geofences	geolocation	false	
39) spbm_ftp113_Geofences	geolocation	false	
40) spbm_ftp121_Geofences	geolocation	false	
41) spbm_ftp275_Geofences	geolocation	false	
42) SPBM_FTP179_Geofences	geolocation	false	
43) SPBM_FTP172_Geofences	geolocation	false	
44) SPBM_FTP477_Geofences	geolocation	false	
45) SPBM_FTP449_Geofences	geolocation	false	
46) SPBM_FTP482_Geofences	geolocation	false	
47) SPBM_FTP483_Geofences	geolocation	false	
48) SPBM_FTP479_Geofences	geolocation	false	
49) SPBM_FTP495_Geofences	geolocation	false	
50) SPBM_FTP271_Geofences	geolocation	false	

Question & Settings	Question Type	Variable Name	Global Variable Name
25) SINCE THE LAST SURVEY:	MULTIPLE_CHOICE	gf_discrimination3	G:LA:gf_discrimination3

Who discriminated against you?

- Display Conditions: If SINCE THE LAST SURVEY: I felt discriminated against. IS Yes
- Question Groups: None
- Response Required: Yes
- Randomize Choice: No Choices
- Seconds to Answer: 0
- Seconds Until Next Button Becomes Active: 0

Breakdown of Each Trigger

Name	Type	Settings
1)	ONCE	Runs from 2024-04-08T11:49:00 UNTIL 2025-04-08T00:00:00.000Z
2) Geofence example trigger	schedule	false
3) UserId: 6567c7d46b8a38a70237d196 - Geofences	geolocation	false
4) UserId: 6567cad06b8a38a70237d4dd - Geofences	geolocation	false
5) UserId: 6567cbae6b8a38a70237d532 - Geofences	geolocation	false
6) UserId: 6568bdd76b8a38a70238648b - Geofences	geolocation	false
7) UserId: 6584306828f1dc791eae5abb - Geofences	geolocation	false
8) UserId: 65b0080e29fd6a9d3466f2b5 - Geofences	geolocation	false
9) UserId: 65d0bc2f38a1f8159ed8253b -	geolocation	false

Geofences		
10) UserId: 65d8d947f7c6ed301283576d - Geofences	geolocation	false
11) UserId: 65dacd5217ddbc9fec6fdade - Geofences	geolocation	false
12) UserId: 65dc2026cc4061e42221d67a - Geofences	geolocation	false
13) UserId: 65de57eb405d00d76d2f03ae - Geofences	geolocation	false
14) UserId: 6660a99c4bbb1eceb296728f - Geofences	geolocation	false
15) UserId: 6658a32b70d761aec4591e9a - Geofences	geolocation	false
16) UserId: 6660a3014bbb1eceb29666bd - Geofences	geolocation	false
17) JT - UserId: 66914ab785c52222d31cd6d1- Geofences	geolocation	false
18) JW Geofences - 8/13/24	geolocation	false
19) JT Geofences - 8/13/24	geolocation	false
20) JH Geofences - 8/29/24	geolocation	false
21) JW Geofences - 8/30/24	geolocation	false
22) JD iOS Geofences - 8/30/24	geolocation	false
23) JD Android Geofences - 8/30/24	geolocation	false
24) JD Android Geofences - 8/30/24 (enter)	geolocation	false
25) JD iOS Geofences - 8/30/24 (enter)	geolocation	false
26) JH Geofences - 8/29/24 (enter)	geolocation	false

27) JT Geofences - 8/13/24 (enter)	geolocation	false
28) JT Geofences - 09/4/24 (enter)	geolocation	false
29) JW Geofences - 8/30/24 (enter)	geolocation	false
30) JD Android Geofences - 8/30/24 (enter) (Copy)	geolocation	false
31) JT Geofences - 10/11/24 (enter)	geolocation	false
32) JH Geofences - 10/11/24 (enter)	geolocation	false
33) JW Geofences - 10/11/24 (enter)	geolocation	false
34) JH Geofences - 10/16/24 (enter)	geolocation	false
35) JD Android Geofences - 10/16/24 (enter)	geolocation	false
36) JT Geofences - 10/16/24 (enter)	geolocation	false
37) spbm_ftp102 _Geofences	geolocation	false
38) spbm_ftp112_Geofences	geolocation	false
39) spbm_ftp113_Geofences	geolocation	false
40) spbm_ftp121_Geofences	geolocation	false
41) spbm_ftp275_Geofences	geolocation	false
42) SPBM_FTP179_Geofences	geolocation	false
43) SPBM_FTP172_Geofences	geolocation	false
44) SPBM_FTP477_Geofences	geolocation	false
45) SPBM_FTP449_Geofences	geolocation	false
46) SPBM_FTP482_Geofences	geolocation	false
47) SPBM_FTP483_Geofences	geolocation	false
48) SPBM_FTP479_Geofences	geolocation	false
49) SPBM_FTP495_Geofences	geolocation	false
50) SPBM_FTP271_Geofences	geolocation	false

Question & Settings	Question Type	Variable Name	Global Variable Name
26)	FIELD_GROUP	int	G:LA:int

- Display Conditions: None
- Question Groups: None
- Response Required: No
- Randomize Choice: No Choices
- Seconds to Answer: 0
- Seconds Until Next Button Becomes Active: 0

Breakdown of Each Trigger

Name	Type	Settings
1)	ONCE	Runs from 2024-04-08T11:49:00 UNTIL 2025-04-08T00:00:00.000Z
2) Geofence example trigger	schedule	false
3) UserId: 6567c7d46b8a38a70237d196 - Geofences	geolocation	false
4) UserId: 6567cad06b8a38a70237d4dd - Geofences	geolocation	false
5) UserId: 6567cbae6b8a38a70237d532 - Geofences	geolocation	false
6) UserId: 6568bdd76b8a38a70238648b - Geofences	geolocation	false
7) UserId: 6584306828f1dc791eae5abb - Geofences	geolocation	false
8) UserId: 65b0080e29fd6a9d3466f2b5 -	geolocation	false

Geofences		
9) UserId: 65d0bc2f38a1f8159ed8253b - Geofences	geolocation	false
10) UserId: 65d8d947f7c6ed301283576d - Geofences	geolocation	false
11) UserId: 65dacd5217ddbc9fec6fdade - Geofences	geolocation	false
12) UserId: 65dc2026cc4061e42221d67a - Geofences	geolocation	false
13) UserId: 65de57eb405d00d76d2f03ae - Geofences	geolocation	false
14) UserId: 6660a99c4bbb1eceb296728f - Geofences	geolocation	false
15) UserId: 6658a32b70d761aec4591e9a - Geofences	geolocation	false
16) UserId: 6660a3014bbb1eceb29666bd - Geofences	geolocation	false
17) JT - UserId: 66914ab785c52222d31cd6d1- Geofences	geolocation	false
18) JW Geofences - 8/13/24	geolocation	false
19) JT Geofences - 8/13/24	geolocation	false
20) JH Geofences - 8/29/24	geolocation	false
21) JW Geofences - 8/30/24	geolocation	false
22) JD iOS Geofences - 8/30/24	geolocation	false
23) JD Android Geofences - 8/30/24	geolocation	false
24) JD Android Geofences - 8/30/24 (enter)	geolocation	false
25) JD iOS Geofences - 8/30/24 (enter)	geolocation	false

26) JH Geofences - 8/29/24 (enter)	geolocation	false
27) JT Geofences - 8/13/24 (enter)	geolocation	false
28) JT Geofences - 09/4/24 (enter)	geolocation	false
29) JW Geofences - 8/30/24 (enter)	geolocation	false
30) JD Android Geofences - 8/30/24 (enter) (Copy)	geolocation	false
31) JT Geofences - 10/11/24 (enter)	geolocation	false
32) JH Geofences - 10/11/24 (enter)	geolocation	false
33) JW Geofences - 10/11/24 (enter)	geolocation	false
34) JH Geofences - 10/16/24 (enter)	geolocation	false
35) JD Android Geofences - 10/16/24 (enter)	geolocation	false
36) JT Geofences - 10/16/24 (enter)	geolocation	false
37) spbm_ftp102 _Geofences	geolocation	false
38) spbm_ftp112_Geofences	geolocation	false
39) spbm_ftp113_Geofences	geolocation	false
40) spbm_ftp121_Geofences	geolocation	false
41) spbm_ftp275_Geofences	geolocation	false
42) SPBM_FTP179_Geofences	geolocation	false
43) SPBM_FTP172_Geofences	geolocation	false
44) SPBM_FTP477_Geofences	geolocation	false
45) SPBM_FTP449_Geofences	geolocation	false
46) SPBM_FTP482_Geofences	geolocation	false
47) SPBM_FTP483_Geofences	geolocation	false
48) SPBM_FTP479_Geofences	geolocation	false
49) SPBM_FTP495_Geofences	geolocation	false

50) SPBM_FTP271_Geofences	geolocation	false

Diary Information

Diary Name	Trigger Name / Type	Responses
Log cigarette	Number of Triggers: 7	0
Log cigarette	Number of Triggers: 9	0
Log other tobacco	Number of Triggers: 5	0
Log smoking locations	Number of Triggers: 13	0

Resource Information

Resource Name	Trigger Name / Type	Responses
Study Contacts	Number of Triggers: 1	0
