# Supplementary material for: App-Based Smoking Urge Reduction Intervention for Young Adults: Protocol Combining a Microrandomized Trial and Conventional Between-Subject Randomized Trial
Source: JMIR Res Protoc. 2025 Sep 23;14:e74388. doi: 10.2196/74388 (PMC12504902; doi:10.2196/74388)
Supplement: Multimedia Appendix 4 [file resprot_v14i1e74388_app4.pdf]

SUMMARY STATEMENT
(Privileged Communication) *Release Date:* 03/24/2020
Revised Date:

Application Number: 1 R01 CA246590-01A1

Principal Investigator

THRUL, JOHANNES

Applicant Organization: JOHNS HOPKINS UNIVERSITY

Review Group:

Meeting Date: 03/05/2020
Council: MAY 2020
Requested Start: 07/01/2020

RFA/PA: PAR18-869
PCC: Y2TC

Project Title: Testing the impact of smartphone-based messaging to support young adult smoking cessation
SRG Action: Impact Score:31 Percentile:16 #
Next Steps: Visit https://grants.nih.gov/grants/next_steps.htm
Human Subjects: 30-Human subjects involved - Certified, no SRG concerns
Animal Subjects: 10-No live vertebrate animals involved for competing appl.
Gender: 1A-Both genders, scientifically acceptable
Minority: 1A-Minorities and non-minorities, scientifically acceptable
Age: 7A-Only Adults, scientifically acceptable

Project Year	Direct Costs Requested	Estimated Total Cost
1		
2		
3		
4		
5		
TOTAL		

ADMINISTRATIVE BUDGET NOTE: The budget shown is the requested budget and has not been adjusted to reflect any recommendations made by reviewers. If an award is planned, the costs will be calculated by Institute grants management staff based on the recommendations outlined below in the COMMITTEE BUDGET RECOMMENDATIONS section.
EARLY STAGE INVESTIGATOR
NEW INVESTIGATOR

1R01CA246590-01A1 Thrul, Johannes

EARLY STAGE INVESTIGATOR
NEW INVESTIGATOR
COMMITTEE BUDGET RECOMMENDATIONS

RESUME AND SUMMARY OF DISCUSSION: This resubmitted application from an Early Stage Investigator seeks to use a clinical trial to test the effectiveness of an intervention using text messages to reduce smoking urges and support quit attempts among young adult smokers. Panel members varied in their assessment of the application's significance. Some considered the application to be highly significant, based on the lack of any existing evidence-based smartphone apps, the high translational potential of the proposed intervention, and the application's strong conceptual foundation that includes an innovative use of location-based messaging. Other reviewers questioned the significance of the application, based on the small sample size in the prior study, evidence suggesting the magnitude of change would be relatively modest, and inadequate evidence supporting the use of the geolocation-based prompts for smoking cessation. The application has a number of strengths, including its well-developed approach for using ecological momentary assessments (EMA) to identify smoking locations, its use of micro-randomization to assess the within-person effects of specific messages, and the resubmission's improved description of the proposed recruitment plans. The inclusion of additional expertise on the investigative team for the EMA component was an additional strength in this resubmission. Some weaknesses were also noted, including concerns about the feasibility of the proposed approach for biochemical verification of smoking status and the potential effects on participant retention due to the intrusive nature of the geolocation data. Following the discussion, a majority of the panel agreed that the application's strengths outweighed its weaknesses, resulting in a study with a high potential impact on the field.

DESCRIPTION (provided by applicant): Tobacco use remains the most important cause of morbidity and premature mortality in the United States. Young adults have high smoking rates and low use of evidence-based smoking cessation services. Smartphones are widely used among young adults and offer a promising strategy to deliver smoking cessation treatment to a large, diverse audience of young adult smokers. Available smoking cessation apps for smartphones are rarely evidence-based and able to deliver intervention content that is tailored to the specific needs of the individual smoker. Little is known how smartphone-based interventions need to be designed and what kind of tailored intervention content they should deliver. While there is evidence for the efficacy of both Cognitive Behavioral Therapy (CBT) and Mindfulness/ Acceptance and Commitment Therapy (ACT) smoking cessation interventions, it is unclear if these approaches are efficacious when implemented in real-time and with young adults. The overall goal of this proposal is to evaluate the efficacy of CBT and ACT-based messages for young adults targeted at specific high-risk situations for smoking. Our team has experience in using smartphones and Ecological Momentary Assessment (EMA) to understand situational predictors of smoking in everyday life, and smoking cessation trials with young adults using Facebook. We have demonstrated feasibility of determining high-risk situations for smoking and delivering tailored messages based on geofence triggers. The specific aims are to: 1) To test CBT and Mindfulness/ACT intervention message efficacy for reducing momentary smoking urges. We will conduct a micro-randomized trial (within-subject randomization) to test the efficacy of CBT and Mindfulness/ACT compared to control messages for reducing smoking urge 15 minutes after message delivery. 2) To test if exposure to urge reduction messages results in changes in smoking behavior over time compared to an EMA only control group. A control group of participants that will complete EMA only without intervention messages will allow us to test, if messages reduce cigarettes per day at end of treatment, 3-, and 6-months follow-up. 3) Explore moderation effects of substance co-use (cannabis, alcohol, other drugs) and exposure to specific location (home, work, bars) on urge reduction message

efficacy. Among intervention group participants, we will explore how message efficacy may be moderated by substance co-use and exposure to specific settings. Smoking onset is now more common among young adults than adolescents and early cessation substantially reduces morbidity and mortality from smoking, making age-appropriate, tailored, and scalable interventions for this high priority population ever more important.

PUBLIC HEALTH RELEVANCE: Effective smoking cessation treatments that reach the target population of young adults are direly needed and smartphone interventions are a promising strategy. Few of the available smoking cessation apps are based on evidence or deliver intervention content tailored to participant-specific high-risk situations for smoking. The aim of this proposal is to test smartphone-delivered messages for smoking cessation and urge reduction among young adults that target specific high-risk situations for smoking and have the potential to curb smoking in this underserved age group.

CRITIQUE 1

Significance: 4
Investigator(s): 1
Innovation: 3
Approach: 5
Environment: 1

Overall Impact: This revised application from an Early Stage Investigator proposes testing of an ecological momentary assessment (EMA)-facilitated cognitive-behavioral therapy (CBT)-based versus acceptance and commitment therapy (ACT)-based tailored messaging mobile application for reducing smoking urges and for reducing smoking among young adult cigarette smokers. This line of work is significant, in that it investigates novel means to intervene with a particularly vulnerable group of smokers. It is innovative in leveraging GPS-based geofencing as a means to identify high-risk locations for smoking via EMA procedures. The design is generally rigorous, including micro-randomization of CBT vs ACT vs control messaging, as well as inclusion of an EMA-only control group. Factors that reduce enthusiasm include the dearth of information on magnitude of change in smoking urges and smoking behavior from these interventions. The closest analogous work cited indicated a 3.8 cigarettes per day reduction with a 14-day mindfulness-based approach. Particularly given that the proposed study would recruit quit-motivated participants, it is unclear whether a modest reduction goal is provides sufficient benefit and value. Additionally, there are limitations inherent in the design, leaving only post-hoc comparisons that could elucidate the effect of the various messaging strategies on post-treatment smoking outcomes. There are also limitations to consideration of assessment of smoking, given that the main outcomes are related to reduction rather than cessation.

1. Significance:

Strengths

- Tobacco smoking remains common and problematic in young adults, and this population rarely engages in evidence-based tobacco cessation programming.
- Evidence suggests that mobile technology messaging, with CBT or ACT approaches, may yield reductions in smoking urges and smoking behavior; delivering this messaging during high-risk situations may potentially yield added benefit. If that is the case, then the proposed work would be a significant advance to the field.

- While smoking cessation apps are common and accessible by young people, few apps have been rigorously tested.

Weaknesses

- Interventions to reduce, rather than cease, smoking may have limited impact on distal health outcomes.
- The added value, compared to embedded burden and potential privacy concerns, of geolocation-based interventions is not clear; this could limit dissemination and uptake.

2. Investigator(s):

Strengths

- The investigative team, led by an Early Stage Investigator, and complemented by experienced researchers with strong track records, appears well-qualified to conduct this line of work.
- A co-I with statistical expertise with EMA-based research has been added.

Weaknesses

- None noted.

3. Innovation:

Strengths

- The use of “geofencing” (based on EMA data on locations where a participant tends to smoke) to trigger just-in-time messaging is innovative.
- The mix of micro-randomization of message types within participants, layered onto a randomization of messaging versus EMA-only groups is an innovative approach to test hypotheses.

Weaknesses

- None noted by reviewer.

4. Approach:

Strengths

- Use of a 2-week EMA observation period to determine locations that are high-risk for smoking is a sound and rigorous approach.
- Sex as a biological variable is considered in the application.
- The proposal’s inclusion of micro-randomization allows for comparison within participants between message types, while overall allowing for the effect of messaging versus the EMA-only control group.
- Consideration of other substance use, which is common in the target age group, is a notable strength.

Weaknesses

- While there is value in biochemical verification of abstinence, this is not among the targeted/anticipated outcomes, and objective assessment of smoking/tobacco reduction is not adequately considered.

- While the study’s design will allow for comparison of message types on proximally-related smoking urges, the main comparison for post-intervention smoking outcomes will be between messaging overall versus EMA-only; the message type comparisons for smoking outcomes are limited to post-hoc dose-response, limiting the potential for interpretation for tailored design of subsequent work.
- The pilot study most informative for the proposed work was a small N=8 proof-of-concept, with little to lend toward potential effect sizes of the intervention compared to less innovative but analogous (at least in CBT, ACT, etc., approach) interventions.

5. Environment:

Strengths

- The environment appears very well suited to the successful completion of the proposed work.

Weaknesses

- None are noted.

Study Timeline:

Strengths

- The proposed timeline appears reasonable for the aims and procedures as described.

Weaknesses

- None are noted.

Protections for Human Subjects:

Acceptable Risks and/or Adequate Protections

- Acceptable consideration of risks.

Data and Safety Monitoring Plan (Applicable for Clinical Trials Only):

Acceptable

- Acceptable DSM plan; no formal DSMB in light of risk considerations.

Inclusion Plans:

- Sex/Gender: Distribution justified scientifically
- Race/Ethnicity: Distribution justified scientifically
- For NIH-Defined Phase III trials, Plans for valid design and analysis:
- Inclusion/Exclusion Based on Age: Distribution justified scientifically
- Acceptable.

Vertebrate Animals:

Not Applicable (No Vertebrate Animals)

Biohazards:

Not Applicable (No Biohazards)

Resubmission:

- The revised application is strengthened via responsiveness to many of the concerns raised on initial review.

Resource Sharing Plans:

Acceptable

Budget and Period of Support:

Recommend as Requested

CRITIQUE 2

Significance: 1
Investigator(s): 1
Innovation: 3
Approach: 3
Environment: 1

Overall Impact: This is a revised R01 application from an Early Stage Investigator. The purpose of this clinical trial is to evaluate the efficacy of a smartphone-based intervention that draws from Cognitive Behavior Therapy (CBT) and Mindfulness/Acceptance and Commitment Therapy (ACT) in effort to reduce smoking urges in real time and in high-risk situations in the population young adults. Ecological momentary assessment (EMA) will be used to evaluate message effectiveness in randomized treatment and control conditions at the end of treatment and at 3- and 6-month follow-ups (aim 2). Within the treatment group, a micro-randomized trial will be conducted that tests within-subjects response within 15 minutes of message receipt, as well (aim 1). Moderation by substance co-use and location is also accounted for (aim 3). The revised application has been responsive to most of the reviews. An additional statistical Co-I and consultant have been added to help with the extensive EMA data management and analysis. Eligibility requirements have been expanded from 18 to 25 to 18 to 30. More detail has been provided on the feasibility of recruitment. The app proposed is one that the research team has used in the past, thus maximizes resources based on existing infrastructure. The justification for using geofencing data, however, is not well demonstrated given their prior work shows urge is more relevant than location in predicting smoking behavior and due to the invasiveness of the measure. Given the lack of evidenced-based smartphone applications aimed at reducing smoking (and other substance co-use, including cannabis) and the large population that could be reached, if specific aims are met, results from this study would have strong impact in efforts towards smoking cessation. Strengths far outweigh the weaknesses noted.

1. Significance:

Strengths

- Age-appropriate, tailored, and scalable intervention proposed for a high-risk population of smokers.

- Strong scientific premise for the intervention-based app – expands prior research on text messaging with little evidenced-based content with CBT- and ACT-based messaging.
- CBT and ACT have been successful in smoking cessation efforts, but unclear if this could generalize to an app-based format. If specific aims are met, results could guide large efforts to reduce smoking cessation.
- Accounts for possible effects on other nicotine use and moderation of intervention success based on poly substance use (e.g., cannabis).

Weaknesses

- None noted.

2. Investigator(s):

Strengths

- PI Thruhl is an Early Stage Investigator with a strong publication record and ample experience in smoking cessation research and EMA.
- Co-I team bring strong expertise (Mendelson on CBT and ACT, Latkin is a senior investigator with over 20-years experience with randomized control designs and EMA, Moran in health education, Co-I Zipunnikov on biostatistics).

Weaknesses

- None noted.

3. Innovation:

Strengths

- Smartphone-based app with empirically-based messaging approach (CBT, ACT).
- Will identify whether CBT vs. ACT-based messaging are more impactful rather than just one or the other in relation to the control condition.
- EMA assessment will be used to identify if the situation is high-risk.
- Inclusion of geofencing data (although, see approach).

Weaknesses

- Ecological momentary assessment is not necessarily novel, but using EMA to evaluate smartphone-based CBT and ACT messaging is innovative.

4. Approach:

Strengths

- Demonstrated feasibility to recruit a diverse, young adult sample from Facebook with strong retention.
- Consideration of sex as a biological variable.
- Use of self-report and biochemical tests to identify substance use.
- Power analyses support proposed sample size.

- Micro-randomization to message within treatment group as well as randomization to treatment and control groups.

Weaknesses

- The application refers to prior research this team has conducted that evaluated geofencing data but that urges were the strongest predictor of smoking behavior and not necessarily locations. Thus, it is unclear why geofencing data is justified and seems particularly invasive.
- Only 65 of the 250 evidenced-based messages have been tested on a sample of only 8 participants using EMA but plans in place to review messages with an advisory board and consultant are acceptable.

5. Environment:

Strengths

- The Johns Hopkins School of Public Health provides all necessary requirements for space, grant management, and researcher needs to execute the study as proposed.

Weaknesses

- None noted.

Study Timeline:

Strengths

- Adequate plan in place.

Weaknesses

- None noted.

Protections for Human Subjects:

Acceptable Risks and/or Adequate Protections

- Adequate protections are in place, although geofencing data proposed is invasive and does not seem to offer particular predictive power in reference to specific aims.

Data and Safety Monitoring Plan (Applicable for Clinical Trials Only):

Acceptable

- Adequate plan in place.

Inclusion Plans:

- Sex/Gender: Distribution justified scientifically
- Race/Ethnicity: Distribution justified scientifically
- For NIH-Defined Phase III trials, Plans for valid design and analysis: Scientifically acceptable
- Inclusion/Exclusion Based on Age: Distribution justified scientifically
- Inclusion plans and plans for design and analysis are acceptable

Vertebrate Animals:

Not Applicable (No Vertebrate Animals)

Biohazards:

Not Applicable (No Biohazards)

Resubmission:

- Mostly responsive to prior reviews, although not all 250 messages have been piloted.

Resource Sharing Plans:

Acceptable

Budget and Period of Support:

Recommend as Requested

CRITIQUE 3

Significance: 4
Investigator(s): 1
Innovation: 3
Approach: 4
Environment: 2

Overall Impact: Young adults have high smoking rates and low use of evidence-based smoking cessation services. Effective smoking cessation treatments are directly needed for young adult smokers and smartphone interventions are a promising strategy. The aim of this proposal is to test smartphone-delivered messages for smoking cessation and urge reduction among young adults that target specific high-risk situations for smoking and have the potential to curb smoking in this underserved age group. The research team comprehensively addressed reviewers' comments throughout the proposal providing clarity to the study aims. The proposed study can improve in the follow areas: 1) provide more supportive evidence (i.e., stronger argument) for the need to compare CBT vs Mindfulness/ACT and the utility of Aim 3, 2) Additional description of the message refinement process and the corresponding pictures is needed, 3) The proposed EMA is demanding on participants so there are concerns about attrition and whether the current retention plan will work, 4) Information was sparse regarding how messages are based on two theories of relapse. Yet, the strengths outweigh the study weaknesses making the study very competitive for funding. The research team and environment are excellent and the study is supplemented with expert consultants. Research team has a strong, solid history for work with young adult smoker and developing tobacco cessation interventions. The intervention app went through feasibility testing. The EMA control group is a positive and important component of the study design which ensures outcomes are due to intervention and not testing. Overall, this is a promising study.

1. Significance:

Strengths

- Research study targets young adult tobacco users, a difficult to reach and retain population that is in need of tobacco cessation interventions that work.

- Background literature review is adequate for proposed study.

Weaknesses

- Background literature could benefit by adding information on utility of mHealth apps that target young adults, specifically young adults' willingness to use mHealth apps, do mHealth apps changed health behaviors of young adults?
- More balanced description of ACT and CBT. Might be helpful to have a table providing example messages for each Mindfulness/ACT and CBT.
- Aim 3 is dedicated to whether co-use of other substances moderates message effectiveness, while this is important, a stronger argument to support the value of this aim would be helpful.

2. Investigator(s):

Strengths

- PI has appropriate expertise and adequate experience with delivering technology based intervention to young adults.
- Solid study team with experience implementing RCTs, developing health communication, and expertise in biostatistics.
- Consultants bring additional expertise to the project.

Weaknesses

- None noted.

3. Innovation:

Strengths

- The GPS tracking and proactive outreach to smoker via push notifications is a good use of new technology and innovative.
- Immediate feedback on messages is unique.
- Testing efficacy of messages during high risk situation is novel.

Weaknesses

- Tailored tobacco messaging and mHealth apps are not particularly novel.

4. Approach:

Strengths

- Strong research history for working with young adult tobacco users and developing tobacco cessation interventions.
- A particularly interesting EMA study predicting smoking patterns based on GPS location.
- Feasibility testing indicates app is easy to use and effective at monitoring smoking behavior. Further pilot participants indicate messages curbed their desire to smoke.
- EMA control group is important to determine the intervention itself did not change the behavior.
- Outcome measure, smoking urge ratings, smoking behavior, and participant cognitive and message ratings are appropriate for study design.

- Other important measures collected via this study are # cigarettes per day and product switching.

Weaknesses

- Initial pilot test would have benefited from more participants (i.e., 10-12 participants).
- Message refinement (i.e., Focus groups) is separate from the study proposal aims but needs to occur prior to Aim 1. How might current database messages change via focus groups and will the messages be cognitively tested at any point?
- The proposal mentions message will have picture and there is no further discussion about how picture will be implemented or tested.
- EMA is demanding for participants with 3 EMAs per day for 30 days. If your retention efforts, participant feedback, incentives, and outreach fail, consider increasing recruitment numbers to buffer unexpected attrition.
- Consider a graphic representation for how using Marlatt's Model of Relapse Prevention and the Vulnerability-Stress Model of Relapse frame the messages used.
- Given the current outcome measures of smoking reduction, is biochemical testing necessary.
- Is there pilot data confirming the biochemical validation method planned is reliable?

5. Environment:

Strengths

- Johns Hopkins is an excellent University with a comprehensive and high quality research environment.
- Glad the project will benefit from outside consulting given project personnel is solely from Johns Hopkins.

Weaknesses

- None noted.

Study Timeline:

Strengths

- Study aims can be accomplished in this time frame.

Weaknesses

- Breaking the timeline into quarter (i.e., 4 quarter per year) allows for a better understanding of the workload and tasks flow.

Protections for Human Subjects:

Acceptable Risks and/or Adequate Protections

- Human subject information is comprehensive and address all IRB concerns

Data and Safety Monitoring Plan (Applicable for Clinical Trials Only):

Acceptable

- DSMP is describe and satisfactory given study aims and sample size

Inclusion Plans:

- Sex/Gender: Distribution justified scientifically
- Race/Ethnicity: Distribution justified scientifically
- For NIH-Defined Phase III trials, Plans for valid design and analysis: Not applicable
- Inclusion/Exclusion Based on Age:

Vertebrate Animals:

Not Applicable (No Vertebrate Animals)

Biohazards:

Not Applicable (No Biohazards)

Resubmission:

- The research team addressed prior reviewer comments comprehensively.

Resource Sharing Plans:
Acceptable

- Reasonable plan for sharing data

Budget and Period of Support:

Recommended budget modifications or possible overlap identified:

- The project team FTEs seem low and suggest the project personnel time is not enough to complete all study aims.

THE FOLLOWING SECTIONS WERE PREPARED BY THE SCIENTIFIC REVIEW OFFICER TO SUMMARIZE THE OUTCOME OF DISCUSSIONS OF THE REVIEW COMMITTEE, OR REVIEWERS' WRITTEN CRITIQUES, ON THE FOLLOWING ISSUES:

PROTECTION OF HUMAN SUBJECTS: ACCEPTABLE

INCLUSION OF WOMEN PLAN: ACCEPTABLE

INCLUSION OF MINORITIES PLAN: ACCEPTABLE

INCLUSION ACROSS THE LIFESPAN: ACCEPTABLE

COMMITTEE BUDGET RECOMMENDATIONS:
A concern was noted that the project personnel did not budget adequate time to complete the project.

Footnotes for 1 R01 CA246590-01A1; PI Name: Thrul, Johannes

Ad hoc or special section application percentiled against "Total CSR" base.

NIH has modified its policy regarding the receipt of resubmissions (amended applications).See Guide Notice NOT-OD-18-197 at https://grants.nih.gov/grants/guide/notice-files/NOT-OD-18-197.html. The impact/priority score is calculated after discussion of an application by averaging the overall scores (1-9) given by all voting reviewers on the committee and multiplying by 10. The criterion scores are submitted prior to the meeting by the individual reviewers assigned to an application, and are not discussed specifically at the review meeting or calculated into the overall impact score. Some applications also receive a percentile ranking. For details on the review process, see http://grants.nih.gov/grants/peer_review_process.htm#scoring.
